# Using field evaluation and systematic iteration to rationalize the accumulation of omega‐3 long‐chain polyunsaturated fatty acids in transgenic *Camelina sativa*

**DOI:** 10.1111/pbi.13867

**Published:** 2022-06-27

**Authors:** Lihua Han, Susana Silvestre, Olga Sayanova, Richard P. Haslam, Johnathan A. Napier

**Affiliations:** ^1^ Plant Sciences Rothamsted Research Harpenden, Herts UK

**Keywords:** Field trials, GM crops, omega‐3, metabolic engineering

## Abstract

The Brassicaceae *Camelina sativa* (gold of pleasure) is now an established niche crop and being used as a transgenic host for a range of novel seed traits. Most notable of these is the accumulation of omega‐3 long‐chain polyunsaturates such as eicosapentaenoic acid (EPA) and docosahexaenoic acid (DHA), fatty acids normally only found in marine organisms. As part of continued efforts to optimize the accumulation of these non‐native fatty acids via seed‐specific expression of algal genes, a new series of iterative constructs was built and introduced into Camelina. Seed fatty acid composition was determined, and the presence of EPA and DHA was confirmed. To provide an additional level of evaluation, full environmental release was carried out on selected events, providing a real‐world gauntlet against which to assess the performance of these novel lines. Composition of the seed oil triacylglycerol was determined by mass spectrometry, allowing for conclusions as to the contribution of different activities to the final accumulation of EPA and DHA. Since these data were derived from field‐grown material, they also represent a robust demonstration of the stability of the omega‐3 LC‐PUFA trait in Camelina. We propose that field trialling should be routinely incorporated in the plant synthetic biology ‘design–build–test–learn’ cycle.

## Graphical abstract

GM Camelina in the field in Hertfordshire, England.

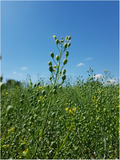

## Introduction

**Figure 1:**
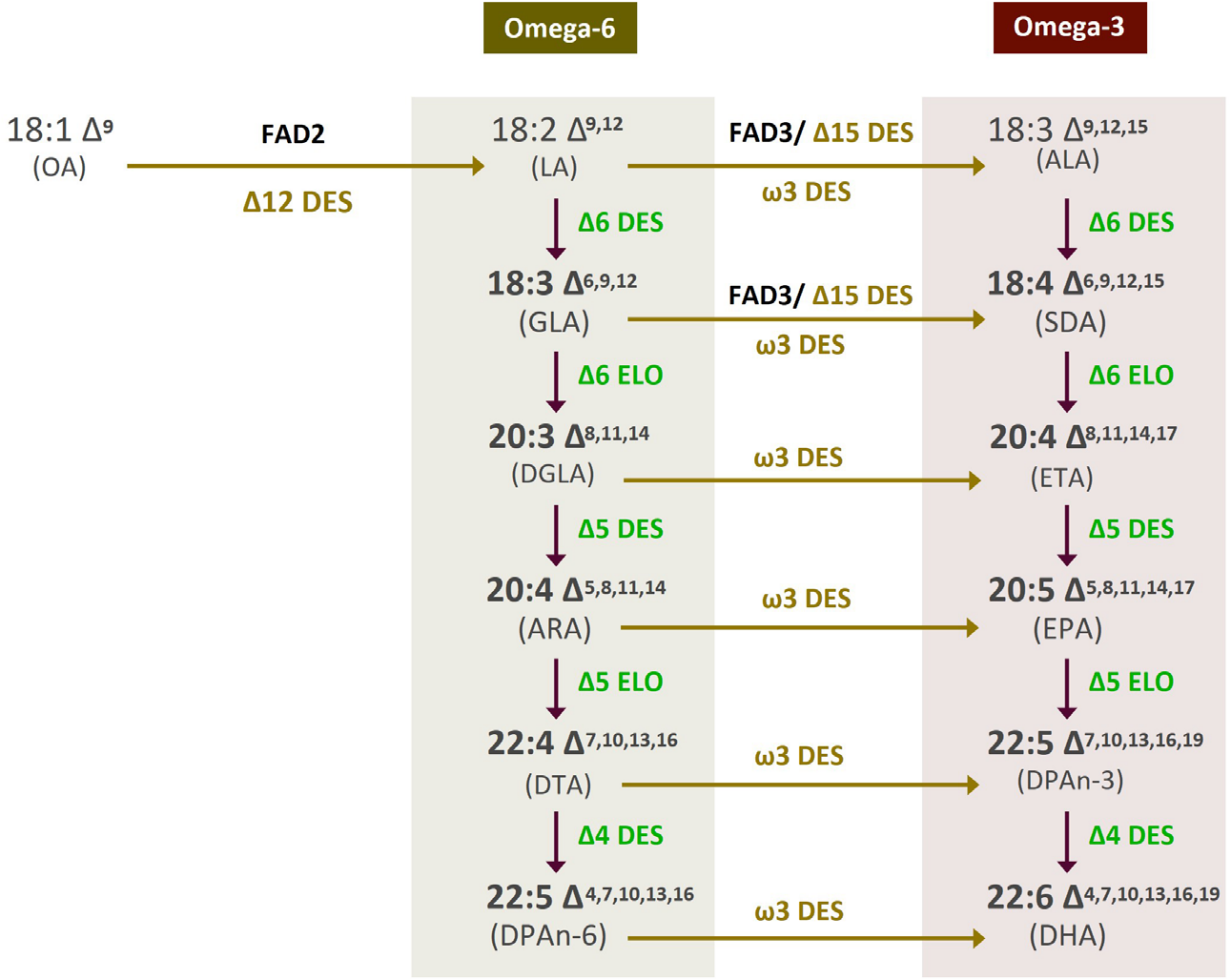
Schematic representation of omega‐3 long‐chain polyunsaturated fatty acid biosynthetic pathway. Schematic representation of omega‐3 long‐chain polyunsaturated fatty acid EPA and DHA biosynthetic pathway. The desaturase and elongase enzymatic conversion of fatty acids and the various routes for substrate flux are indicated. Endogenous activities (FAD2, FAD3) are shown in black. Transgenic activities are as follows: Δ6 DES = Δ6‐desaturase; Δ6 ELO = Δ6‐elongase; Δ5 DES = Δ5‐desaturase; Δ12 DES = Δ12‐desaturase; w3 DES = w3‐desaturase; Δ15 DES = Δ15‐desaturase; Δ5 ELO = Δ5‐elongase; and Δ4 DES = Δ4‐desaturase.

Camelina is an excellent chassis for the metabolic engineering of high value and useful seed oil traits (reviewed by Yuan and Li, 2020) including omega‐3 long‐chain polyunsaturated fatty acids (omega‐3 LC‐PUFAs, also known as omega‐3 fish oils), accumulating significant levels of target fatty acids such as eicosapentaenoic acid (EPA; 20: 5n‐3) and docosahexaenoic acid (DHA; 22:6n‐3) (Napier *et al.,* 2015; Ruiz‐Lopez *et al.,* 2014). Studies have demonstrated the stability of this trait in both the glasshouse and the field under environmental release (Han *et al.,* 2020; Usher *et al.,* 2015, 2017) and also the suitability of such plant‐derived oils as total replacements for oceanically derived fish oil in the diets of farmed fish such as salmon, sea bass and sea bream (Betancor *et al.,* 2021; reviewed by Tocher *et al.,* 2019). Similarly, these modified Camelina oils have recently been shown to be equivalent to fish oils in human dietary studies (West *et al.,* 2021). Importantly, the combined levels of EPA and DHA in transgenic Camelina were significantly higher in comparison with those recently reported in parallel efforts to engineer similar accumulation in the more established commodity crop canola, indicating Camelina is a superior boutique platform for the production of these oils (Napier *et al.,* 2019; Petrie *et al.,* 2020). For both species (Camelina and canola), the accumulation of EPA and DHA was achieved by the seed‐specific expression of the non‐native omega‐3 LC‐PUFA biosynthetic pathway, a suite of genes predominantly derived from marine microorganisms, which in the most minimal form requires the presence of five distinct and sequential enzyme activities to convert the C18 fatty acids ubiquitous to higher plants into the non‐native C20+ PUFA forms (Figure 1) (Petrie *et al.,* 2014; Ruiz‐Lopez *et al.,* 2014). Progress has been made in demonstrating the successful metabolic engineering of this pathway in Arabidopsis, soybean, canola and Camelina, although this has predominantly been based on an ad hoc approach to construct design (Park *et al.,* 2017; Petrie *et al.,* 2020; Qi *et al.,* 2004; Usher *et al.,* 2017). Irrespective of the chassis used to host the accumulation of non‐native omega‐3 LC‐PUFAs, these advances represent some of the most complex examples of plant metabolic engineering and applied synthetic biology to date and demonstration of the potential of ‘green factories’, as witnessed by the recent full regulatory approval of this trait in canola (Napier *et al.,* 2019, Napier and Sayavova, 2020; Petrie *et al.,* 2020).

**Figure 2:**
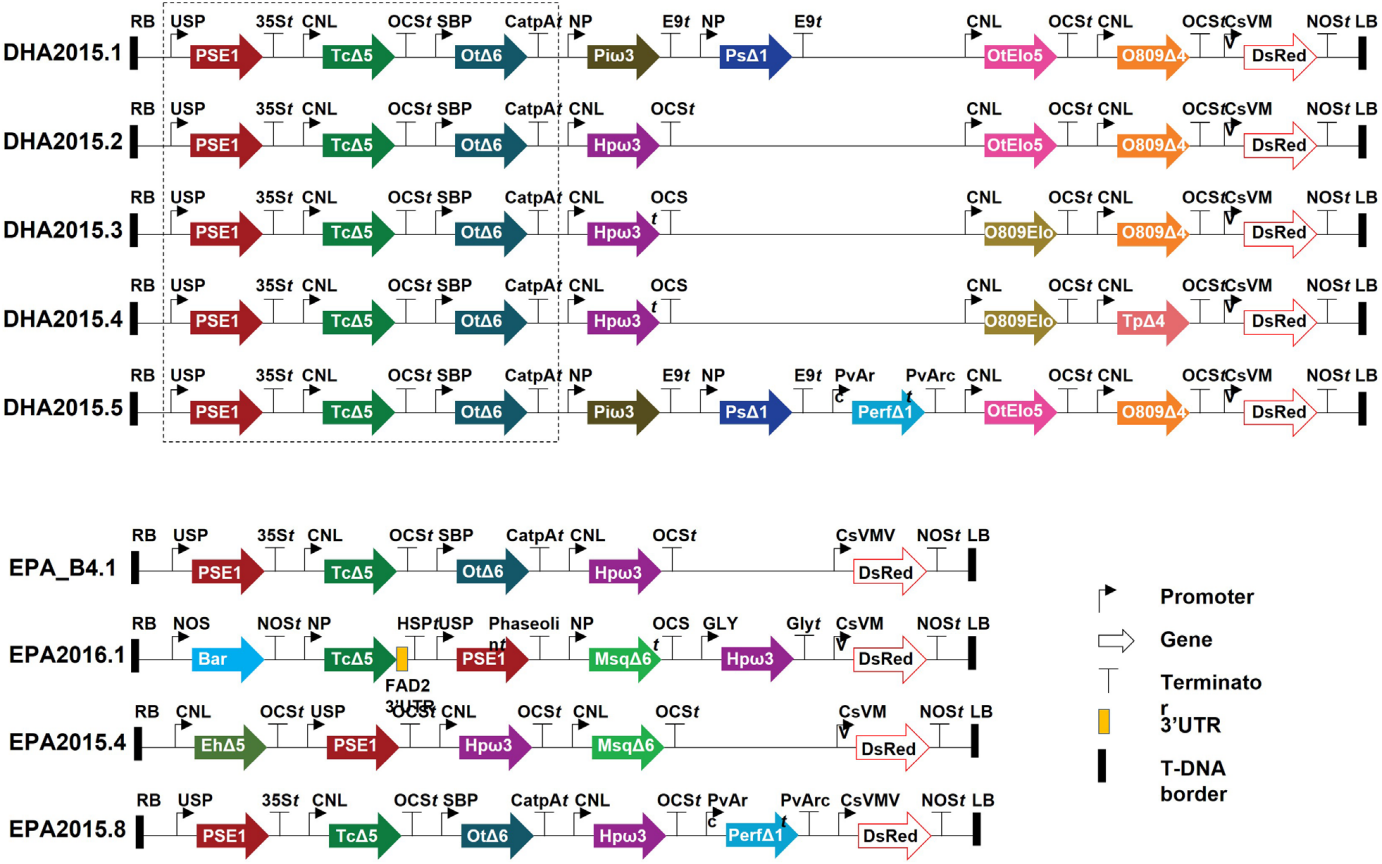
Schematic representation of nine different omega‐3 constructs used to direct the synthesis of EPA and DHA. The different DNA parts promoters, genes, terminators, 3′UTR, and T‐DNA borders are illustrated by symbols. Different enzyme activities in Figure 1 encoded by synthetic genes under the control of seed‐specific promoters were assembled into binary vectors as described and introduced into *C. sativa* cv. Celine. For DHA2015.1–5, the shared Δ6 DES, Δ6 ELO and Δ5 DES gene cassettes are squared by the dashed line. Abbreviations: CNL, conlinin 1 promoter for the gene encoding the *L. usitatissimum* 2S storage protein conlinin; USP, promoter region of the unknown seed protein of *V. faba*; SBP, sucrose binding protein 1800 promoter from *V. faba*; NP, napin seed‐specific promoter from *Brassica napus*; PvArc, Arcelin‐5 seed storage protein promoter from *Phaseolus vulgaris*; GLY, 11S seed storage protein glycinin promoter from *Glycine max*; OtΔ6, Δ6‐elongase from *O. tauri*; MsqΔ6, Δ6‐desaturase from *Mantoniella squamata;* PSE1, Δ6‐elongase from *P. patens*; TcΔ5, Δ5‐desaturase from *Thraustochytrium sp*.; EhΔ5, Δ5‐desaturase from *E. huxleyi*; PsΔ12, Δ12‐desaturase from *P. sojae*; Piw3, w3‐desaturase from *P. infestans*; Hpω3, w3‐desaturase from *H. parasitica*; PerfΔ15, Δ15‐desaturase from *P. frutescens*; OtElo5, Δ5‐elongase from *O. tauri*; O809Elo5, Δ5‐elongase from *Ostreococcus RCC809*; O809D4, Δ4‐desaturase from *Ostreococcus RCC809*; TpD4, Δ4‐desaturase from *T. pseudonana*; FAD2 3′UTR, 3′UTR from *C. sativa* FAD2 gene; and 35S*t*, OCS*t*, CatpA*t*, E9*t*, PvArc*t*, NOS*t*, HSP*t*, Phaseolin*t* and Gly*t* are terminators.

To obtain a better understanding of the contribution of different transgene products to the flow of substrates through this heterologous biosynthetic pathway, and also to further refine the fatty acid profile of our transgenic Camelina, we set out to adopt a systematic approach to construct design and validation similar to the ‘design–build–test–learn’ (DBTL) rationale used in synthetic biology (Haslam *et al.,* 2016), with the important addition of environmental release. We therefore took our previous construct DHA2015.1 (Han *et al.,* 2020) as the prototype for improvement, building several variant forms in which individual changes to genetic elements are sequentially accumulated (Figure 2). DHA2015.1 comprises seven genes encoding activities for the synthesis of EPA and DHA, including the five primary biosynthetic enzymes (Δ6‐, Δ5‐ and Δ4‐desaturases; Δ6‐ and Δ5‐elongases) and two additional activities (Δ12‐desaturase and ω3‐desaturase) that are proposed to be involved in maximizing substrate flux (Napier *et al.,* 2015; Figures 1 and 2); the expression of each of these genes is directed by a seed‐specific promoter; see Han *et al*. (2020) for details of both the molecular composition and performance of DHA2015.1. Similarly, we took our construct EPA_B4.1, which directs the synthesis of EPA (Ruiz‐Lopez *et al.,* 2014; Usher *et al.,* 2017), but lacks two activities (Δ5‐elongase and Δ4‐desaturase) to convert EPA to DHA (Figures 1 and 2), and also generated iterations from that particular prototype. All the resulting variations were evaluated both in the glasshouse and in the field, providing real‐world data on the performance of these transgenic lines. As discussed below, we now advocate for the routine incorporation of environmental release data into the DBTL cycle for plant synthetic biology and engineering biology.

## Results and discussion

Previous studies by us and others (Petrie *et al.,* 2014; Usher *et al.,* 2015, 2017) have provided insights into the optimal configuration of the heterologous omega‐3 LC‐PUFA biosynthetic pathway in Camelina. In particular, the requirement for an acyl‐CoA‐dependent Δ6‐desaturase has been found to be crucial, avoiding the accumulation of Δ6‐desaturated C18 PUFAs in phospholipids and circumventing the so‐called ‘substrate‐dichotomy’ bottleneck associated with lipid‐dependent Δ6‐desaturases and resulting in inefficient elongation and subsequent accumulation of C20+ PUFAs (Abbadi *et al.,* 2004; Napier *et al.,* 2015). However, beyond this requirement, relatively little is known in terms of the individual contributions of primary and secondary biosynthetic activities in different heterologous systems. In an attempt to address this deficiency, we carried out post hoc analyses of the directed accumulation of EPA and DHA in the seeds of transgenic plants and concluded that the Δ12‐desaturase from *Phytophthora sojae* (PsΔ12) present in DHA2015.1 and other iterations might be dispensable, since this activity not only depleted the oleic acid (18:1n‐9) pool but also generated excessive omega‐6 fatty acids via the synthesis of linoleic acid (18:2n‐6). Therefore, this activity was omitted from three new derivatives (named DHA2015.2–4); these three constructs contained one less expression cassette than DHA2015.1, (i.e. lacking the PsΔ12 activity) (Figure 2). In addition, in DHA2015.2 the *Phytophthora infestans* ω3‐desaturase (Piw3) present in DHA2015.1 was replaced with a similar activity from *Hyaloperonospora parasitica* (Hpw3), on the basis that this new activity provided a broader substrate preference towards C18+ n‐6 PUFAs (Senger *et al.,* 2015). Similarly, in DHA2015.3, the *Ostreococcus tauri* ELO5 Δ5‐elongase activity was substituted with an ortholog from *Ostreococcus* RCC809 (O809Elo5), to hypothetically work in tandem with the *Ostreococcus* RCC809 Δ4‐desaturase from the same species, as well as the Hpw3/Piw3 exchange, whereas in DHA2015.4, the *Ostreococcus* RCC809 Δ4‐desaturase was replaced with a similar Δ4‐desaturase activity from the taxonomically distinct diatom *Thalassiosira pseudonana,* in conjunction with the Hpw3 and O809Elo5 substitutions (Figure 2). Finally, we also made a new variant of DHA2015.1 in which all the original activities were retained, along with an additional FAD3 Δ15‐desaturase from *Perilla frutescens* (PerfΔ15) (Chung *et al.,* 1999) – this construct was named DHA2015.5 (Figure 2). This last iteration was designed to increase the levels of α‐linolenic acid (18:3n‐3), which serves as the main substrate for the heterologous LC‐PUFA biosynthetic pathway when expressed in Camelina. By this systematic approach, we hoped to better define critical steps in the biosynthetic pathway and optimal combinations of transgene‐derived activities.

As well as aiming to optimize the performance of the DHA2015.1 construct, we adopted a similar approach to the systematic improvement of construct EPA_B4.1 (Figure 2), used to direct the synthesis of EPA and was previously shown to accumulate useful levels (~15% of total seed fatty acids) of this C20 omega‐3 PUFA in transgenic camelina (Usher *et al.,* 2017). EPA_B4.1 contained exactly the same four genes present in DHA2015.2, encoding the four biosynthetic activities (Δ6‐desaturase, Δ6‐elongase, Δ5‐desaturase and ω3‐desaturase), which comprise the so‐called EPA module, but lacking the Δ5‐elongase and Δ4‐desaturase activities, which comprise the DHA module, thus directing the synthesis of EPA but not DHA. In the new iteration named EPA2016.1 the *O. tauri* Δ6‐desaturase was replaced with a similar activity from *Mantoniella squamata* (MsqΔ6). Equally, in the EPA2015.4 iteration, the Δ5‐desaturase from *Thraustochytrium* spp. was replaced with a similar activity from *Emiliania huxleyi*, in addition to the substitution of the *O. tauri* Δ6‐desaturase with the MsqΔ6 activity. In EPA2015.8, the EPA_B4.1 prototype was amended by the sole addition of the PerfΔ15 FAD3 Δ15‐desaturase activity, analogous to DHA2015.5. All constructs, both EPA and DHA iterations, were assembled into a binary vector as previously described using a Gateway‐based system and introduced into Camelina via *Agrobacterium*‐mediated transformation (Ruiz‐Lopez *et al.,* 2014). The seed‐specific presence of non‐native omega‐3 long‐chain polyunsaturated fatty acids EPA and DHA in glasshouse‐grown T2and T3 material clearly indicates the efficient accumulation of these important fatty acids (as determined by GC‐FID), as well as potentially interesting differences between the constructs (Figures S1, S2 and S6). Most notably, in the case of DHA2015.1–5 series, the mean ratio of EPA:DHA varied markedly (ranging from 0.9, 0.7, 4.6, 7.4, 1.6, listed sequentially). Since all constructs generated high levels of EPA + DHA at T2, it was decided to proceed with the evaluation of T3 seeds under ‘real‐world’ (i.e. non‐contained) field conditions.

**Figure 3:**
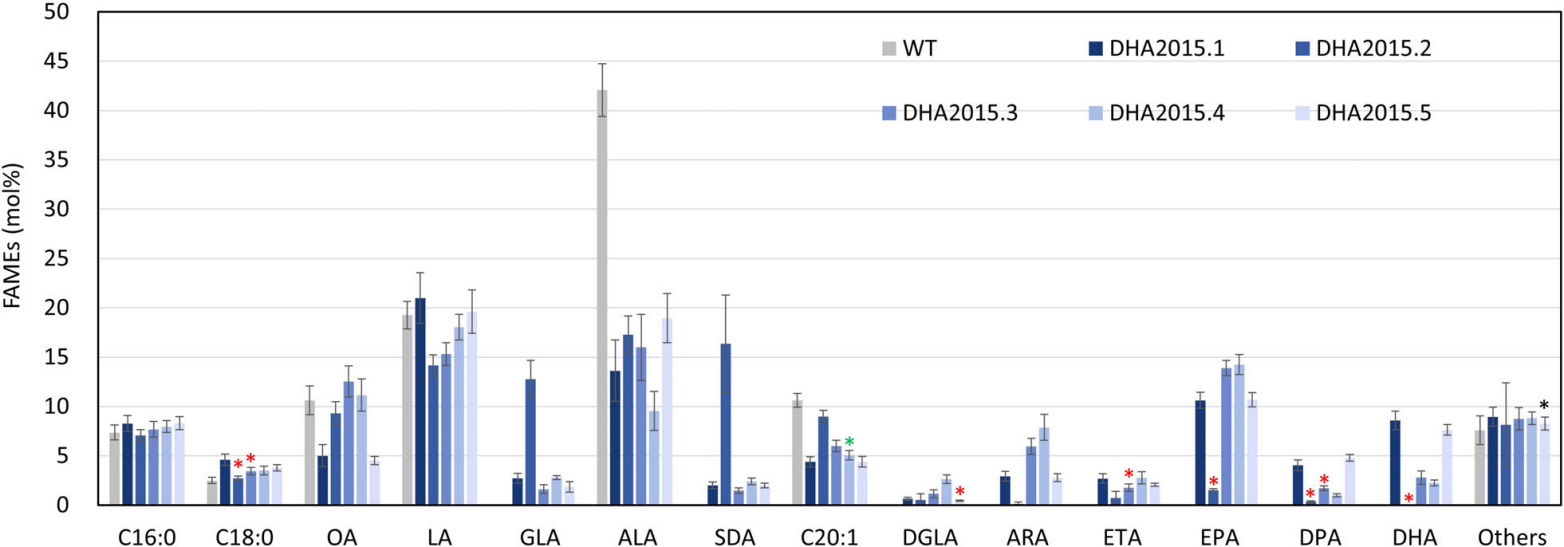
Seed fatty acid profiles for the DHA series, compared collectively with DHA2015.1 prototype. FAMEs prepared from seed lots (samples) from the bulked harvests of individual field trial plots were analysed by GC‐FID, with identification of fatty acids confirmed by GC–MS and comigration with authentic standards. Values are mean ± SE, *n* = 15.

**Figure 4:**
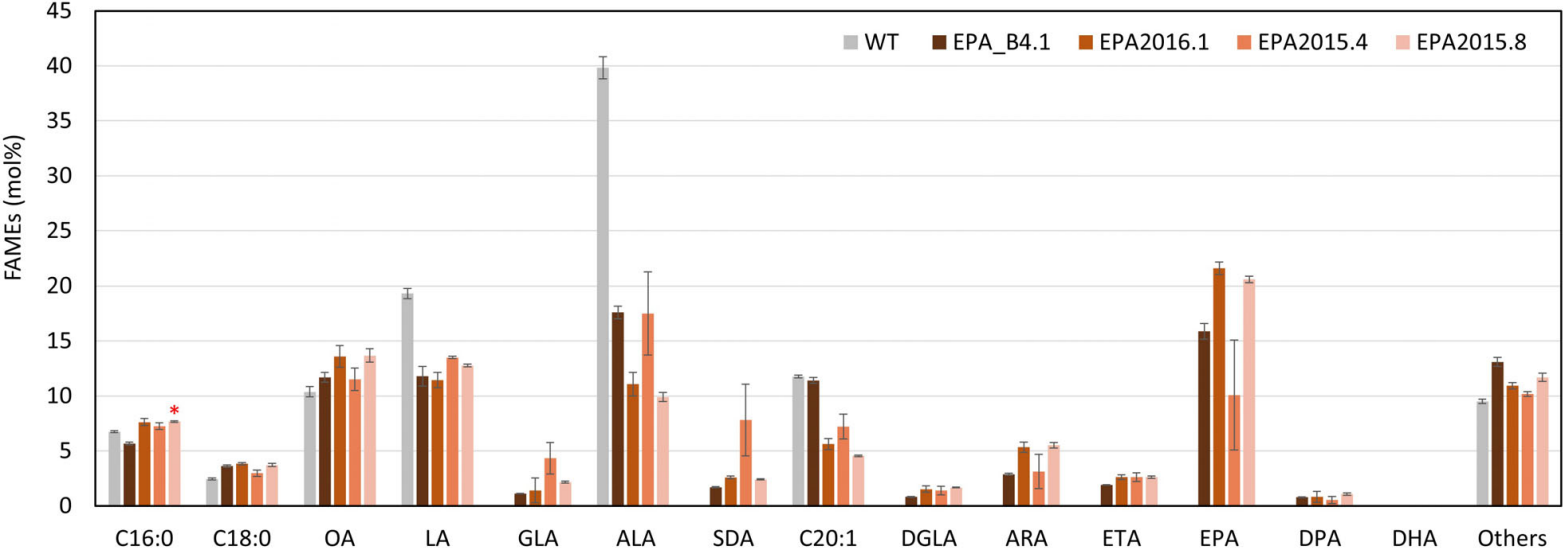
Seed fatty acid profiles for EPA series, compared collectively with EPA_B4.1 prototype. FAMEs prepared from seed lots (samples) from the bulked harvests of individual field trial plots were analysed by GC‐FID, with identification of fatty acids confirmed by GC–MS and comigration with authentic standards. Values are mean ± SE, *n* = 3.

A field evaluation of these new GM iterations was undertaken and benchmarked against the DHA2015.1 and EPA_B4.1 prototypes. Approval to carry out an environmental release at the Rothamsted GM trial site in Harpenden was obtained from DEFRA (Consent 18/R8/01) and carried out as previously described (Usher *et al.,* 2017). Plants were sown in replicated blocks (see Figure S3 for plot layout) with plants grown to maturity and their seeds harvested using a plot combine. The constituent fatty acids of threshed and cleaned seeds were analysed by the chromatographic resolution of fatty acid methyl esters (FAMEs) by GC‐FID (Usher *et al.,* 2015), compared with DHA2015.1 grown in the same trial, allowing for group and pairwise comparisons (Figures 3 and S4). In the case of the EPA lines, these were benchmarked against field release data for EPA‐B4.1 obtained in 2017 (Figures 4 and S5). Details of the environmental conditions during the course of the field trial are presented here (Table S1). As an additional reference, the same seed batches were used for both field trial and glasshouse experiments (Figure S6).

In terms of the accumulation of non‐native fatty acids, several observations are clear from this systematic analysis of field‐grown material, which has the advantage of representing the sum of all the variation and challenges encountered in the natural environment (as distinct from the artificially stable conditions found in glasshouses or controlled environment cabinets). Firstly, in the case of DHA2015.2 (the simplest iteration) resulted in the unexpected accumulation of C18 Δ6‐desaturated fatty acids such as GLA and SDA and an absence of EPA and DHA (Figure 3; Figure S1; see also Figure S4 for side‐by‐side comparison of the different iterations). However, we believe that this phenomenon is an event‐specific example of transgene‐silencing (reviewed by El‐Sappah *et al.,* 2021 and citations therein), most likely because of the (random) site of insertion of the T‐DNA in the host genome, as opposed to the replacement of the Piw3 ω3‐desaturase with the Hpw3 activity. Evidence for this specific fatty acid profile being as a consequence of post‐transcriptional gene silencing (PTGS) is given further weight by comparison of the field data for T4 and from the T3 GH‐grown seeds with the initial T2 seeds (Figure S6), in which the latter clearly shows high levels of EPA + DHA. Strikingly, this has all but disappeared in the subsequent T3 generation of DHA2015.2. Importantly, the same transgenes and associated regulatory elements are present in other DHA2015 series constructs without the overaccumulation of GLA and SDA in DHA2015.2 (Figures 2 and 3; cf. within Figure S4), indicating that this is not likely to be a *cis*‐mediated effect.

In the case of DHA2015.3 (Figure 3; Figure S4), the switching of the Δ5‐elongase activity to one derived from *Ostreococcus* RCC809 had a negative impact on the levels of omega‐3 LC‐PUFAs, reducing the accumulation of C22 DPA (22:5n‐3) and DHA with a concomitant build‐up of EPA. This is likely due to reduced elongation of EPA, as opposed to increased synthesis of that fatty acid, since DHA2015.3 also contains the substitute Hpw3 activity, which, based on the elevated levels of arachidonic acid (ARA; 20:4n‐6), may be slightly less efficient in the current configuration at the conversion of omega‐6 PUFAs to their omega‐3 form, compared with the Piw3 activity present in DHA2015.1 (and DHA2015.5). Although it had been hoped that some benefits might be observed using sequences from the same species within the DHA module, this was not the case.

In the case of DHA2015.4, the exchange of the *Ostreococcus* RCC809 Δ4‐desturase (the last reaction on the biosynthetic pathway for DHA – Figure 1) with a functionally equivalent activity from *T. pseudonana* had a further deleterious impact on the accumulation of DHA, indicating that this new activity was inferior to the O809Δ4 present in DHA2015.1. Collectively, these data indicate that the DHA2015.1 prototype is highly efficient at directing the synthesis of EPA and DHA, with minimal accumulation of undesired intermediates. However, it is worth noting that constructs DHA2015.2, DHA2015.3 and DHA2105.4 all lacked the PsΔ12 desaturase, responsible for the conversion of oleic acid to linoleic acid, but the absence of this activity was negligible in terms of the levels of EPA + DHA observed in the field. Similarly, there was a small decline in the levels of linoleic acid, compared to DHA2015.1, and a concomitant doubling of levels of oleic acid to restore these to that found in WT. As discussed below, such a profile is desirable for a number of applications, including aquafeed diets, and also confirms our prediction that the Δ12‐desaturase is dispensable.

**Figure 5:**
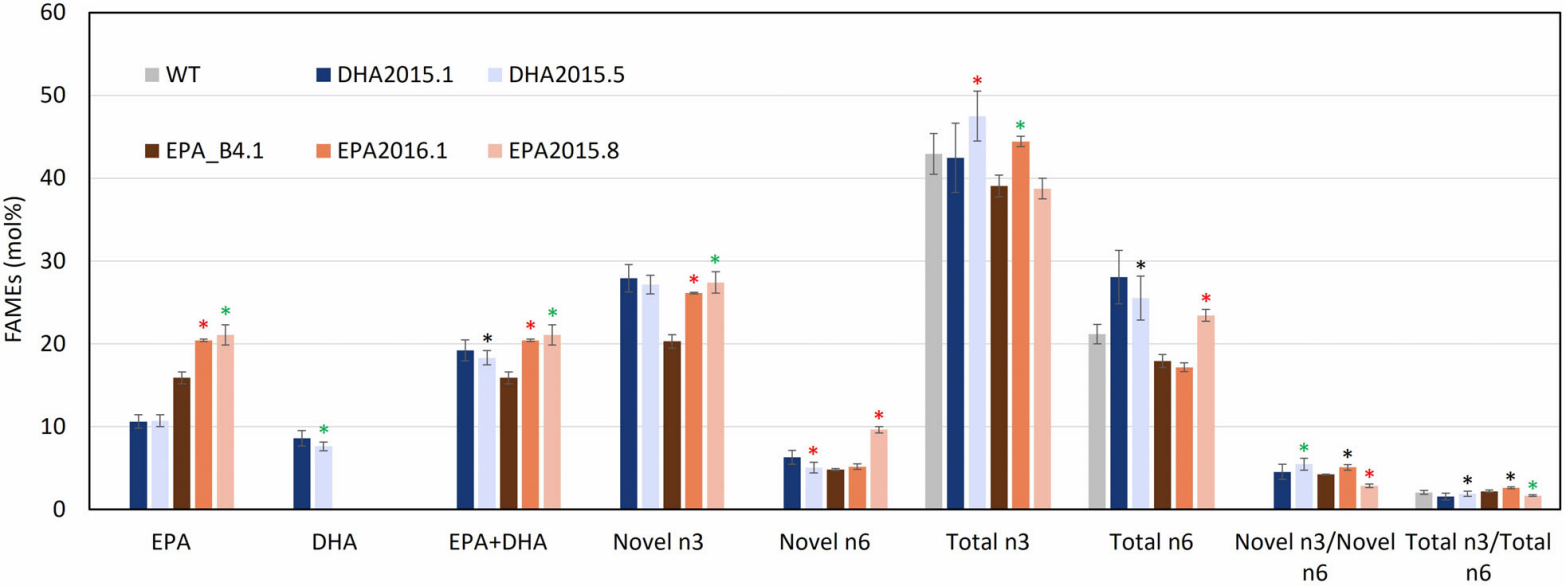
Oil quality parameters of WT, DHA2015.1, DHA2015.5, EPA2015.8 and EPA2016.1 lines. Novel n3 includes SDA + ETA+EPA + DPA + DHA; novel n6 includes GLA + DGLA+ARA; total n3 includes SDA + ETA+EPA + DPA + DHA + ALA+C20:3n3; and total n6 includes GLA + DGLA+ARA + LA + C20:2n6. Plot was further derived from Figures 3 and 4 data.

The final iteration in the DHA series (DHA2015.5) included an additional FAD3 Δ15‐desaturase (responsible for the conversion of linoleic acid to α‐linolenic acid), resulting in the only new construct, which matched the DHA2015.1 prototype over a number of generations, in terms of the accumulation of non‐native n‐3 C20+ LC‐PUFAs (Figures 5 and S6). DHA2015.5 produced increased levels of DPA, as well as C18 ALA, increasing the overall n‐3 levels of this seed oil (Figure 5). However, it may require further investigations to confirm this enhancement and to determine the respective genotype and environment (G × E) contributions. Irrespective of that, the fact that these improvements were observed in the field is indicative of a robust and useful phenotype in terms of novel seed oil traits.

In the case of the systematic improvements to the EPA construct EPA_B4.1, three iterations were evaluated (Figure 2; Figure 4 – see also Figure S5 for side‐by‐side comparisons). The first iteration (EPA2016.1) was the replacement of the OtΔ6 desaturase with the similar activity from *Mantoniella squamata* (MsqΔ6) – this resulted in higher levels of EPA and a concomitant decrease in ALA, indicating that the MsqΔ6 was not only superior to OtΔ6, but also (unlike the latter) preferred omega‐3 substrates (ALA) compared to omega‐6 (LA) (Sayanova *et al.,* 2012). The second iteration, EPA2015.4, additionally replaced the *Thraustochytrium* Δ5‐desaturase with the similar activity from *Emiliania huxleyi* (EhΔ5) (Ruiz‐Lopez *et al.,* 2015) though this resulted in the slight accumulation of eicosatetraenoic acid (ETA; 20:4n‐3) indicating perhaps less efficient Δ5‐desaturation of C20 elongation products. Overall, the performance of EPA2015.4 was inferior to the previous iteration and benchmark line EPA_B4.1. The final variant, EPA2015.8, was analogous to DHA2015.5, with the addition of the FAD3 Δ15‐desaturase PerfΔ15 activity to the EPA_B4.1 prototype – this gave a fatty acid profile with increased EPA, but unexpectedly, a reduction in ALA. However, this reduction in ALA could not be assigned to the presence of the PerfΔ15 activity, since a similar reduction was also observed in EPA2016.1, which lacks this FAD3 transgene (Figure 2). Interestingly, although all of the EPA series contain the Hpw3 activity instead of Piw3, there is some variation in the perceived impact of this activity and the conversion of ARA to EPA (see also comments below) – most noticeably more effective in EPA_B4.1 and EPA2015.4 than in EPA2015.8 and EPA2016.1; there is no clear correlation as to the reason for this although minor difference in regulatory elements (seed‐specific promoters and terminators) may partially explain this.

Based on the sum of all these data, several conclusions can be drawn. Firstly, the individual activities that comprise DHA2015.1 were well chosen and in terms of directing the synthesis of EPA and DHA perform to a level significantly higher than that observed previously in Arabidopsis, canola or camelina (Napier *et al.,* 2019; Petrie *et al.,* 2020; Tocher *et al.,* 2019) – these performances are also stable over a number of generations (Figure S6) and demonstrate a range of desirable metrics in terms of the omega‐3 content present in the seeds (Figure 3). Secondly, based on the systematic replacement of all the activities other than ELO6 present in DHA2015.1 and EPA_B4.1, the most impactful positive change is obtained from the *M. squamosa* Δ6‐desaturase, whereas substitutions with O809Elo5, EhΔ5, and TpΔ4 all showed no obvious improvement on the seed fatty acid profile compared to DHA2015.1. In the case of the MsqΔ6 desaturase, this high activity was unexpected, since previous characterization by others of the identical protein sequence had revealed a very low activity in Arabidopsis (<0.1% of EPA in seeds; Hoffmann *et al.,* 2008). Whilst it is hard to reconcile these two differing outcomes, they could arise from the use of different promoters and/or the absence of codon optimization in the 2008 study. Irrespective of that, our data demonstrate (contrary to the prior art) that the *M. squamosa* Δ6‐desaturase is a very active enzyme suitable for metabolic engineering of omega‐3 LC‐PUFAs in transgenic Camelina.

Another unexpected change was a consequence of using the Hpw3 ω3‐desaturase activity instead of Piw3 in the DHA2015 series – not only did this result in reduced DHA but also somewhat elevated C20 arachidonic acid (ARA; 20:4n‐6). Pathway schema for the synthesis of EPA and DHA usually envisages the linear flow of substrates through both omega‐6 and omega‐3 ‘tracks’ prior to ω3‐desaturation as a final step at C22 but our data would indicate that omega‐6 biosynthetic intermediates such as C20 ARA may also significantly contribute to the final levels of both EPA and DHA, with ω3‐desaturation potentially contributing at multiple points in the pathway.

As a proxy for estimating the maximum potential of any given transgenic construct, as well as indicating any dynamic interactions of the new metabolic pathway, we profiled the fatty acids in individual seeds selected from the pooled harvest for each construct, via GC‐FID of FAMEs. 30 seeds for each construct/plot were analysed individually, and the resulting fatty acid profiles rank‐ordered on the basis of DHA accumulation. By this approach, it is possible to observe the amount of variation within a genetically fixed line, indicating the maxima and minima of the construct under study. In addition, these plots allow for a simple visual summary of any apparent concomitant changes in other fatty acids present in the sample. For example, in the case of the single‐seed analysis for DHA2015.1, rank‐ordering of the samples for DHA unsurprisingly also shows an almost identical trend for EPA (i.e. seeds that are high in DHA are also high in EPA; Figure S7). Interestingly, there is a marked inverse correlation between LA and DHA/EPA, with the highest EPA + DHA seeds having the lowest LA (within the sample cohort). Unexpectedly, in the case of DHA2015.1, there was no obvious correlation between ALA and DHA or EPA levels, given that the former is the substrate for the synthesis of the latter (Figure S7) although this likely also has a developmental component given the asynchronous synthesis of LA and ALA (Pollard *et al.,* 2015). Further evidence for both omega‐3 and omega‐6 C18 fatty acids contributing to the ultimate accumulation of C20+ omega‐3 PUFAs was also provided by examination of similar single‐seed rank–order plots from other lines. In the case of the DHA2015.2 construct, the particular transgenic event taking into the field displayed strong silencing associated with the specific loss of the ELO6 Δ6‐elongating activity PSE1 from *Physcomitrella patens*, the second reaction in the biosynthesis of EPA (Figure 2). As discussed above, the precise basis for the silencing of this transgene activity is unclear, but it provides a serendipitous insight into the flow of substrate fatty acids into the heterologous pathway. Notably, the initiating step (Δ6‐desaturation, which is apparently unaffected by silencing) occurs with almost equal prevalence on omega‐3 and omega‐6 C18 fatty acids, as indicated by the accumulation of GLA (omega‐6) and SDA (omega‐3) Δ6‐desaturated fatty acids (Figure S7, starred). Thus, the first committed step on the LC‐PUFA biosynthetic pathway consumes both omega‐6 and omega‐3 substrates, although ultimately the bulk of these intermediates is converted to the omega‐3 form. In that respect, the observation by Pollard *et al*. (2015) that in Camelina the endogenous FAD3 Δ15‐desaturase is expressed later in seed development than the FAD2 Δ12‐desaturase may also indicate a temporal disconnect, which could be enhanced by appropriately expressed transgenic activities.

Further evidence for the contribution of both omega‐3 and omega‐6 substrates to the ultimate accumulation of EPA + DHA is also provided by the single‐seed analysis for the DHA2015.3 and DHA2015.4 constructs (Figure S7). In both these constructs, synthesis of DHA is reduced compared to the DHA2015.1 benchmark by the substitution of the *O. tauri* ELO5 activity with a similar one from Ostreococcus RCC809. Although these two activities show very similar performance in yeast, the suboptimal performance of O809Elo5 Δ5‐elongase in transgenic Camelina is witnessed by a reduction in C22 PUFAs in both DHA2015.3 and DHA2015.4 iterations, due to the poor elongation of C20 Δ5‐desaturated fatty acids such as EPA. Notably, in both these iterations, the C20 omega‐6 fatty acid arachidonic acid (ARA) is seen to accumulate, most obviously in DHA2015.4. Thus, although the C18 ALA (omega‐3) provides a substantial substrate to EPA and DHA, as witnessed by the inverse relationship between ALA and EPA/DHA in the single‐seed analysis, metabolites of the omega‐6 pathway contribute too. In the case of the accumulation of ARA in DHA2015.3/4, presumably in a more optimal configuration (such as DHA2015.1), this C20 PUFA would undergo elongation to 22:4n‐6, followed by ω3‐desaturation to DPAn‐3 and thence to DHA. Therefore, although maximizing flux through both pathways (omega‐3, omega‐6) is critical for the successful reconstitution of LC‐PUFA biosynthesis, it is equally apparent that the capacity to convert omega‐6 to omega‐3 forms at the different steps (C18, C20, C22) in the pathway plays an important role too.

In addition to these observations, the single‐seed analysis confirmed the impact of the Δ12‐desaturase on reducing levels of OA, clearly apparent in DHA2015.1 and DHA2015.5 (Figure S7). The product of the Δ12‐desaturase, LA (omega‐6), is only modestly increased, and in general, LA seems to be the fatty acid, which is under the strongest homeostatic control (as previously noted by us; Usher *et al.,* 2017). Equally, the presence of a FAD3 activity (converting LA to ALA) in DHA2015.5 resulted in an ALA level increased by ~5% compared with DHA2015.1, but the higher ALA content did not result in higher EPA and DHA in the seed oil.

Additional insights into the synthesis and deposition of target fatty acids such as EPA and DHA can be obtained from the analysis of individual triacylglycerol (TAG) species in seed oil by electrospray ionization mass spectrometry (ESI‐MS/MS). Previously, we have used this approach to identify up to 98 different TAG species in our engineered lines, including tri‐DHA. Compositional characterization of TAG species containing EPA or DHA (identified by the ESI‐MS/MS neutral loss of 20:5 or 22:6 fatty acids during analysis) allows additional insights into the flux of fatty acids into this dominant form of storage lipid. In view of the very large data sets that are generated by these experiments and the complex nature of the resulting outputs, we only focussed our characterizations on comparing the benchmark DHA2015.1 with the best iteration, DHA2015.5. Similarly, we compared the EPA2016.1 construct with the EPA2015.8. Since both these pairs of constructs generated very similar total seed fatty acid profiles, we hoped that the increased resolution provided by specific identification of individual TAGs might provide an extra level of insight. In addition, the use of this profiling method will also reflect variation resulting from environmental factors, and, as such, is well suited to the analysis of field‐grown material subject to the vagaries of real‐world weather conditions (see Table S1 for details).

**Figure 6:**
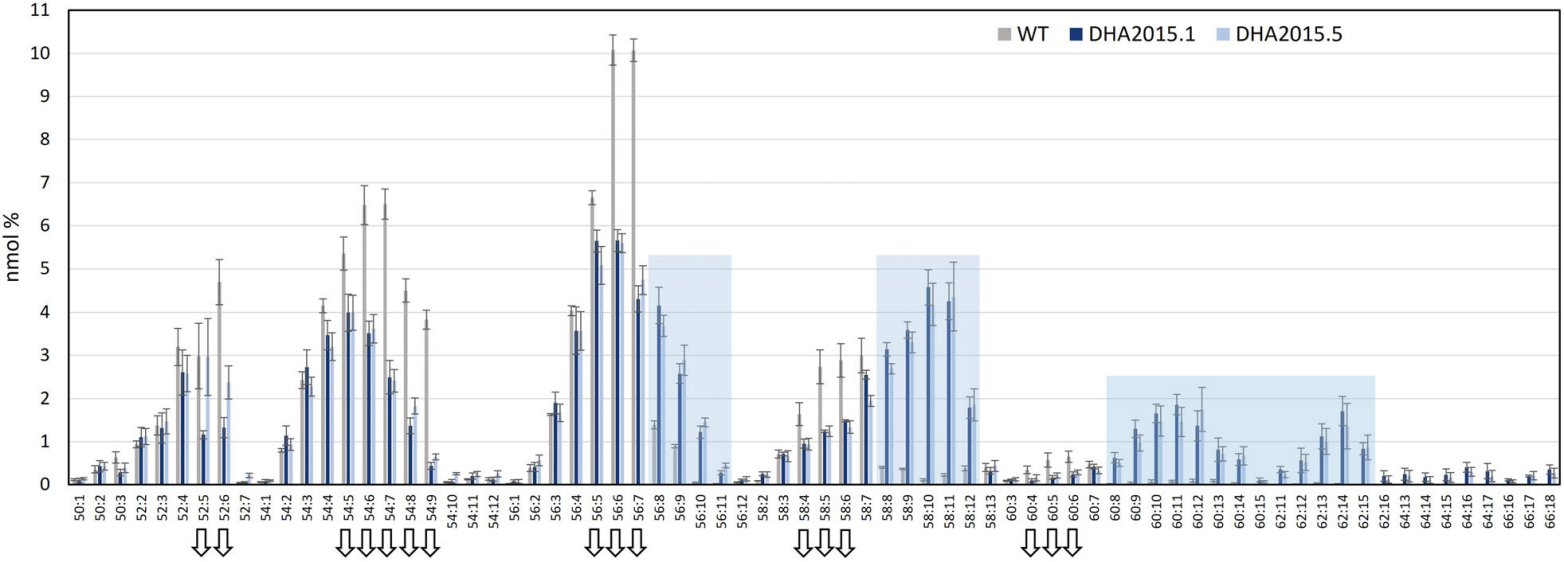
Analysis of triacylglycerols from WT, DHA2015.1 and DHA2015.5 seeds of *C. sativa*. Individual TAG molecular species from transgenic or control lines were identified by ESI‐MS/MS neutral loss survey scan with each TAG species represented by the total number of fatty acid carbon atoms: desaturations, as previously described (Usher *et al.,* 2017). Values are mean ± SE, *n* = 3.

A three‐way comparison of the TAGs of WT, DHA2015.1 and DHA2015.5 from the seed oil of plants grown in the field at Rothamsted confirmed the previously observed shift in the TAG profile associated with the accumulation of EPA and DHA (Han *et al.,* 2020), with a marked increase in the number of species containing 8+ double bonds and a simultaneous enrichment in the C58+ TAGs in both GM lines (Figure 6). This is entirely consistent with the modified fatty acid profile of DHA2015.1 and DHA2015.5 and reflects the accumulation of C20+ LC‐PUFAs such as EPA and DHA in the triacylglycerols. For example, WT Camelina seed oil is effectively devoid of any TAG species of 60 carbons or more and containing at least 8 double bonds, whereas both DHA2015.1 and DHA2015.5 contain a suite of novel TAGs (C60‐66, 8–18 double bonds) (Figure 6). These include what can only be tri‐DHA (66:18), albeit at low abundance, as well as many other non‐native TAGs. Further elucidation of the composition of these TAGs can be derived from the mass spectrum data, determining whether particular fatty acids are present in any given TAG. Using this approach, it was confirmed that the C58 PUFA species contained EPA but not DHA, whereas the C62 PUFAs predominantly contain DHA rather than EPA (Figure S9). It should be noted that this analysis does not determine the structural position of individual fatty acids (sn‐1, 2 or 3) on the glycerol backbone. However, in many cases, plausible assignments can be made, based on the possible permutations. For example, 58:10 and 58:11 are two abundant novel TAGs present in both DHA2015.1 and DHA2015.5, and it is likely these both comprise di‐EPA (20:5_20:5) and either 18:0 or 18:1. Although requiring further structural confirmation, these inferred TAG compositions would indicate a diverse and accommodating pathway for the incorporation of these non‐native fatty acids into neutral lipids and TAG, since occupancy of more than a single position on the glycerol backbone would indicate a biosynthetic route that is derived from either DAG or PC intermediates. Interestingly, side‐by‐side comparison of the TAG profiles for DHA2015.1 and DHA2015.5 revealed only very minor differences, predominantly in the levels of endogenous TAGs (notably 52:5 and 52:6) (Figure S10).

**Figure 7:**
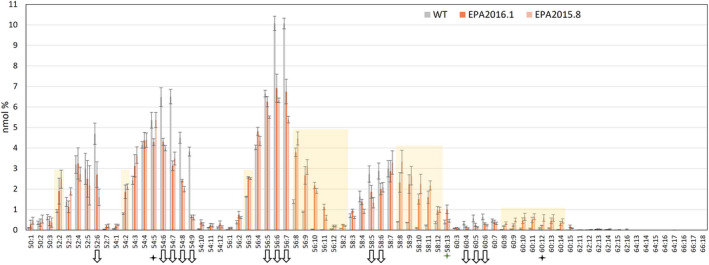
Analysis of triacylglycerols from WT, EPA2016.1 and EPA2015.8 seeds of *C. sativa*. Individual TAG molecular species from transgenic or control lines were identified by ESI‐MS/MS neutral loss survey scan with each TAG species represented by the total number of fatty acid carbon atoms: desaturations, as previously described (Usher *et al.,* 2017). Values are mean ± SE, *n* = 3.

Similar analysis of the seed TAG composition of EPA2016.1 and EPA2015.8 compared with WT revealed an analogous profile for the two transgenic lines, with a range of C58‐60 novel species being generated, as well as the accumulation of C56 species (Figure 7). In the case of both the DHA and EPA lines, two endogenous TAG species (54:6 and 54:7) are significantly reduced in all transgenic lines, presumably since the constituent fatty acids are metabolized to LC‐PUFAs. Given that ALA but not LA is much less abundant in these lines, it is likely that these TAG species are di‐ALA (18:3_18:3) and containing 18:0 or 18:1. However, it is possible that these TAGs comprise other acyl‐compositions (such as 18:3_18:2_18:1) or that they are a mixture of multiple different configurations. As observed for the DHA iterations, a side‐by‐side comparison of TAGs from EPA2016.1 and EPA2015.8 revealed no qualitative differences between the two iterations, despite the presence of an additional activity (FAD3 Δ15‐desaturase) in both EPA2015.8 and DHA2015.5 (Figure S11). At face value, the presence of this enzyme, nominally to enhance the conversion of LA to ALA, brings little additional benefit, presumably on account of the already significant contribution of the endogenous Camelina FAD3 genes (although it is conceivable that expression under a different seed‐specific promoter might yet prove beneficial). Equally, it is possible that contributions from the transgene‐derived FAD3 are masked, most obviously with respect to the synthesis of SDA (18:4n‐3), which can be generated by Δ6‐desaturation of ALA or Δ15‐desaturation of GLA.

When comparing the analysis of TAG species from DHA2015.1 and DHA2015.5 lines with distributions in WT, it is clear that endogenous TAG assembly pathways accommodate non‐native fatty acids. However, identifying the specific entry points of these novel fatty acids into TAG remains elusive. Oil seeds have both acyltransferase and transacylase mechanisms (acyl‐CoA‐dependent and acyl‐CoA‐independent) for the acylation of DAG to TAG, and research effort has continued to understand the network of enzymes and pools that define TAG assembly (Regmi *et al.,* 2020). Recently, Pollard and Shachar‐Hill (2022) have used [^14^C/^13^C] glycerol labelling to describe TAG synthesis in maturing Camelina embryos, demonstrating a clear precursor/production relationship between PC and DAG and TAG. Pathways of EPA and DHA synthesis supply novel fatty acids to both these pools and onward assembly into TAG. However, opportunities for entry of novel fatty acids into TAG could include the (inter)conversion of PC to DAG (for TAG synthesis) via CPT and PDCT. Alternatively, PC acyl editing mechanisms could increase the EPA and DHA content of the acyl‐CoA pool. Indeed, editing of TAG itself (Bhandari and Bates, 2021) might provide opportunities for the remodelling of TAG and incorporation of EPA and DHA. In their interpretation, Pollard and Shachar‐Hill (2022) suggest two distinct pathways of embryo TAG synthesis. A major system, representing about 75 to 80% of the total TAG biosynthetic flux and a second, minor pathway, could represent TAG synthesis in the epidermal layer. Although enrichment of EPA and DHA in TAG likely proceeds via both routes, the second pathway may contain novel lipid biosynthesis routes (e.g. bifunctional GPATs; Yang *et al.,* 2012) and editing activities that support the accumulation of novel fatty acids in TAG. Understanding how this interpretation of TAG synthesis in Camelina impacts efforts to successfully engineer novel fatty acid synthesis in seed remains uncertain, although data from MALDI‐MS imaging studies indicate a spatial asymmetry in the distribution of both endogenous and non‐native TAGs (Marmon *et al.,* 2017; Usher *et al.,* 2017). Future experiments are required to determine the flux of novel fatty acids, for example EPA + DHA, into TAG.

## Conclusions

Here, we present data from a systematic analysis of the individual activities, which collectively contribute to the efficient production of omega‐3 LC‐PUFAs in transgenic camelina. Importantly, our data are derived from both contained and field‐grown transgenic plants, providing increased confidence of both the robust nature of the omega‐3 trait and the results described in this study. A number of key observations have been arrived at. Firstly, it is interesting to note that the transgenic activity of heterologous genes derived from the same source organism does not necessarily all perform to the same standard, exemplified by the highly active O809Δ4 compared with the less effective O809Elo5, despite having presumptively co‐evolved in their original host. The reason for this lack of benefit is unclear but is likely a reflection of individual activities having a disproportionate impact on the total flux through this biosynthetic pathway (Pollard and Shachar‐Hill, 2022). This also provides supports our ‘pick‐and‐mix’ approach to combining the best activities from different organisms rather than attempting to reconstitute a homologous interactome. Whilst the latter approach has some precedents from other systems, too little is currently known about the flux of acyl intermediates through the omega‐3 LC‐PUFA biosynthetic pathway to enable a knowledge‐driven approach here (Xu *et al.,* 2019; Zhang and Fernie, 2020).

Secondly, we surprisingly observed that the omega‐6 pathway can, in the presence of an efficient ω3‐desaturase, contribute significant substrates to the ultimate synthesis of EPA and DHA. However, in the absence of such a ‘sweeper’ activity, the lack of substrate preference for omega‐3 vs omega‐6 fatty acids shown by the initiating *O. tauri* Δ6‐desaturase can generate the unwanted accumulation of omega‐6 fatty acids such as ARA and GLA. In that respect, the substitution of that activity with an orthologue from *Mantoniella* proved to be beneficial, albeit unexpected. It remains to be determined whether MsqΔ6 is effective at directing the accumulation in Camelina of DHA as it is EPA, but there is no reason to expect otherwise. Our studies using lipidomic approaches also allowed us to empirically determine the contribution of ancillary activities such as the Δ12 and Δ15 desaturases to the ultimate accumulation of LC‐PUFAs in seed oil. Based on MS profiling of individual TAG species from field‐grown material of all our constructs, the contribution of the Δ12‐desaturase is revealed as predominantly reducing levels of monounsaturated fatty acids. Intriguingly, there is not a direct proportional correlation between the decline in those fatty acids and the enhancement of any other fatty acids, for example LA or omega‐6 fatty acids, implying that this product pool is actively turned over and metabolized. At the same time, of all the fatty acids present in the camelina seed, LA is the one that is the most unchanged, whatever the transgene configuration (including +/− Δ12 desaturase or FAD3/Δ15 desaturase). The transgene‐derived Δ12‐desaturase clearly modulates the ratio of oleic acid to linolenic acid, and whilst this does not (for reasons that are not obvious) result in significant flux into downstream fatty acids, being able to alter these levels is potentially useful. In particular, oils that are incorporated into aquafeed diets can benefit from having high monounsaturated fatty acids, in terms of solubility, and also ease of catabolism by the animals ingesting these lipids. In that respect, the presence of a Δ12‐desaturase, which is reducing the monounsaturated fatty acid pool (such as oleic acid) without actively increasing the levels of EPA and DHA, is ultimately undesirable and represents an additional transgene to be considered during regulatory approval. For that reason, we consider the Δ12‐desaturase to be an unnecessary element when used in the absence of a suitable transgene‐derived FAD3 activity.

Similarly, field evaluation of the contribution of the FAD3/Δ15‐desaturase to the enhanced accumulation of EPA and DHA pointed towards a tentatively positive role for this activity, but MS TAG profiling reveals only very minor differences resulting from the additional presence of this activity. Based on the minimal impact of this activity in our EPA constructs (cf. EPA2016.1 vs EPA2015.8) as opposed to the modest positive contributions observed in DHA2015.5, it is likely that the *P. sojae* Δ12‐desaturase present only in the latter construct also contributes to generating available substrate for the Δ15‐desaturase. This fits well with our understanding of the substrate preferences of these enzymes for phospholipid‐linked fatty acids (Lindberg‐Yilmaz *et al.,* 2017). This may be beneficial for avoiding the premature incorporation of intermediate fatty acids into TAG. Finally, the benefit of carrying out field‐based studies means our prototypes have already undergone initial validation as being ‘real‐world‐ready’ and suitable lines can now be considered for further evaluation and scale‐up. In that respect, we would strongly advocate for the more routine testing of transgenic plants under field conditions since this is an essential step in confirming the efficacy of the trait under investigation. But perhaps more importantly, plants have evolved to respond to the natural gauntlet of biotic and abiotic stresses, and such variables should be actively incorporated in the DBTL cycle as an expanded component of the ‘test’ phase. Collectively, such approaches will allow for a more robust and meaningful approach to translating fundamental research discoveries into potential solutions for the major challenges (food security, climate emergency) facing our populations. And whilst studies such as these may not reveal novel processes, they demonstrate the critical first steps on the journey from discovery to commercial product.

## Experimental procedures

### Plant material and growth conditions

*Camelina sativa* (cv. Celine) was used in all experiments. Plants grown in the glasshouse were maintained in controlled conditions at 23 °C day/18 °C night and 50%–60% humidity, and kept under a 16‐h photoperiod (long day), with supplemental light provided when ambient levels fell below 400 μmol/m^2^/s. Harvest usually occurred 100 days after sowing.

### Vector construction

For DHA2015.1 and EPA_B4.1 construct assembly, the method has described in papers (Han *et al.,* 2020; Usher *et al.,* 2017). Based on DHA2015.1, the Δ12‐desaturase gene from *Phytophthora sojae* (PsΔ12; Senger *et al.,* 2015) and ω3‐desaturase from *Phytophthora infestans* (Piw3; Wu *et al.,* 2005) were substituted by an ω3‐desaturase *Hyaloperonospora parasitica* (Hpω3; Senger *et al.,* 2015) to produce DHA2015.2. Based on DHA2015.2, the *Ostreococcus tauri* Δ5‐elongase (OtElo5; Meyer *et al.,* 2004) was substituted by an *Ostreococcus* RCC809 Δ5‐elongase (O809Elo5) to produce DHA2015.3. Based on DHA2015.3, the *Ostreococcus* RCC809 Δ4‐desaturase (O809Δ4; Vaezi *et al.,* 2013) was replaced by the *T. pseudonana* Δ4‐desaturase (TpΔ4; Tonon *et al.,* 2005) to produce DHA2015.4. Based on DHA2015.1, an additional Δ15‐desaturase from *Perilla frutescens* (PerfΔ15; Chung *et al.,* 1999) was cloned into DHA2015.1 T‐DNA to produce DHA2015.5. For EPA series constructs, the EPA_B4.1 *Ostreococcus tauri* Δ6‐desaturase (OtΔ6; Domergue *et al.,* 2005) was replaced by a *Mantoniella squamata* Δ6‐desaturase (MsqΔ6; Hoffmann *et al.,* 2008), and the *Thraustochytrium* sp. Δ5‐desaturase (TcΔ5; Bauer *et al.,* 2012) was replaced by an *Emiliania huxleyi* Δ5‐desaturase (EhΔ5; Ruiz‐Lopez *et al.,* 2015) to produce EPA2015.4. PerfΔ15 was added to EPA_B4.1 to produce EPA8. EPA2016.1 cloning method is different. We first gene‐synthesized a large DNA fragment from GenScript (GenScript Corporation, NJ, www.genscript.com), then used restriction enzyme cloning to change the individual gene(s). All open reading frames for desaturases and elongases were resynthesized by GenScript and codon‐optimized for expression in Arabidopsis. All the construct T‐DNA contains a DsRed selection marker. For details of promoters, terminators and other DNA part information, see Figure 2.

### Generation of transgenic plants

Transgenic *C. sativa* lines were generated as previously described (Ruiz‐Lopez *et al.,* 2014). The designed vectors were transferred into *Agrobacterium tumefaciens* strain AGL1. *C. sativa* inflorescences were immersed in the Agrobacterium suspension for 1 min without applying any vacuum. Transgenic seeds expressing the EPA and DHA pathway were identified by visual screening for DsRed activity. Seeds harvested from transformed plants were illuminated using a green LED light. Fluorescent seeds were visualized using a red lens filter.

### Field trials

Field experiments conducted at Rothamsted Research in 2018 (Harpenden, Hertfordshire, U.K.; grid reference TL120130) were carried out as previously described (Usher *et al.,* 2015, 2017), under DEFRA consent 18/R8/01. The detailed GM field locations, weather and sowing date were described in the Supplementary data (Table S1).

### Fatty acid analysis

Total fatty acids in seed batches were extracted and transmethylated according to previous methods (Ruiz‐Lopez *et al.,* 2014). We randomly chose one replicate plot for each GM line for GC‐FID FAME analysis. For the DHA2015.1–5, we used 30 single DsRed seeds with WT control. For EPA2016.1, EPA2015.4 and EPA2015.8, we sampled 3 biological replicates, 10 DsRed seeds/replicate, with WT control. For the greenhouse data, we analysed 4 single DsRed seeds from each line for T2 generation, 3 replicates; 10 DsRed seeds/replicate for T3 generation. Methyl ester derivatives of total fatty acids extracted were analysed by gas chromatography–FID (flame ionization detection), and the results were confirmed by GC–MS and comigration with authentic standards. Unless stated otherwise, for all the experimental data analysis, the values of each Camelina line were given as mean value ± standard error, where n refers to the number of biological replicates.

### Quantitative analysis (ESI‐MS/MS) of seed triacylglycerol

Lipids were extracted from seed by placing the ground samples (20 seeds) in 1 mL of hot (85 °C) isopropanol for 10 min. The homogenate was centrifuged at 300 *g* for 15 min at room temperature, the supernatant was collected, and the pellet was re‐extracted with isopropanol/chloroform (1:1 v/v) and washed with 1 mL of 1 M KCl and then 2 mL water. The solvent was evaporated under nitrogen, and the dry lipid extract was dissolved in 1 mL of chloroform. TAG molecular species were then infused into the mass spectrometer (QTRAP 4000, SCIEX) and were defined by the presence of one acyl fragment and the mass/charge of the ion formed from the intact lipid (neutral loss profiling). TAGs were detected as [M + NH4+] ions by a series of different neutral loss scans, targeting losses of fatty acids. This approach allows identification of one TAG acyl species and the total acyl carbons and total number of acyl double bonds in the other two chains (Ruiz‐Lopez *et al.,* 2014). The procedure does not allow identification of the other two fatty acids individually nor the positions (sn‐1, sn‐2, or sn‐3) that individual acyl chains occupy on the glycerol backbone. TAGs were quantified following background subtraction, smoothing, integration, isotope deconvolution and comparison of sample peaks with those of the internal standard (using Lipid‐View™ software, SCIEX). The data were normalized to the internal standards tri15:0 and tri19:0 (Nu‐Chek‐Prep, Elysian, MN). The profiling samples were prepared by combing 25 uL of the total filtered seed lipid extract with 975 uL of isopropanol/methanol/50 mm ammonium acetate/dichloromethane (4:3:2:1). Samples were infused at 15 uL/min with an autosampler (CTC‐PAL, CTC Analytics). The scan speed was 100 u/s. The collision energy, with nitrogen in the collision cell, was +25 V; declustering potential was +100 V; entrance potential was 14 V; and exit potential was +14 V, respectively. Sixty continuum scans were averaged in the multiple channel analyser mode. For product ion analysis, the first quadrupole mass spectrometer (Q1) was set to select the TAG mass and Q3 for the detection of fragments fragmented by collision‐induced dissociation. The mass spectral responses of various TAG species are variable, owing to differential ionization of individual molecular TAG species. For all analyses, gas pressure was set on ‘low’, and the mass analysers were adjusted to a resolution of 0.7 L full‐width height. The source temperature was 100 °C; the interface heater was on, and +5.5 kV was applied to the electrospray capillary; the curtain gas was set at 20 (arbitrary units); and the two ion source gases were set at 45 (arbitrary units). No response corrections were applied to the data. The data were normalized to the internal standards tri15:0 and tri19:0 (Nu‐Chek Prep, Elysian, MN).

## Conflict of interest

JAN acts as a scientific advisor for Yield10 Biosciences and has previously provided ad hoc consultancy for BASF Plant Sciences. The authors are listed as inventors on patent applications filed by Rothamsted Research.

## Author contributions

LH, SS and RH collected the experimental data. OS and JN designed the constructs. All authors contributed to the writing of this manuscript.

## Supporting information


**Table S1** Details of field trial (location, weather, harvest date).
**Figure S1** T3 greenhouse grown seeds fames data for WT and DHAs lines DHA2015.1‐5. Values are mean ± SE, *n* = 3.
**Figure S2** T3 greenhouse grown seeds fames data for WT and EPAs lines EPA2015.4, EPA2015.8 and EPA2016.1. Values are mean ± SE, *n* = 3.
**Figure S3** 2018 field trial plot map on the Rothamsted experimental farm Appletree location.
**Figure S4** 2018 field trial different DHAs lines DHA2015.2‐4 comparing with DHA2015.1 FAMEs. Values are mean ± SE, *n* = 3
**Figure S5** 2018 field trial different EPAs lines EPA2016.1, EPA2015.4 and EPA2015.8 comparing with EPA_B4.1 FAMEs. Values are mean ± SE, *n* = 3.
**Figure S6** Omega‐3 LC‐PUFA content in DHAs and EPAs lines of different generations.
**Figure S7** Single seed fatty acid composition analysis from the 2018 field trial DHA2015.1‐5 lines.
**Figure S8** Single seed fatty acid composition analysis from the 2018 field trial EPA2016.1, EPA2015.4 and EPA2015.8 lines.
**Figure S9** Analysis of triacylglycerols from DHA2015.1, DHA2015.5, EPA2016.1 and EPA2015.8 seeds of *C. sativa*.
**Figure S10** Analysis of triacylglycerols from DHA2015.1 and DHA2015.5 seeds of *C. sativa*.
**Figure S11** Analysis of triacylglycerols from EPA2016.1 and EPA2015.8 seeds of *C. sativa*.
