# Supplementary material for: Using field evaluation and systematic iteration to rationalize the accumulation of omega‐3 long‐chain polyunsaturated fatty acids in transgenic Camelina sativa
Source: Plant Biotechnol J. 2022 Jun 27;20(9):1833–52. doi: 10.1111/pbi.13867 (PMC9398312; doi:10.1111/pbi.13867)
Supplement: Supplementary file 1 — Table S1 Details of field trial (location, weather, harvest date). Figure S1 T3 greenhouse grown seeds fames data for WT and DHAs lines DHA2015.1‐5. Values are mean ± SE, n = 3. Figure S2 T3 greenhouse grown seeds fames data for WT and EPAs lines EPA2015.4, EPA2015.8 and EPA2016.1. Values are mean ± SE, n = 3. Figure S3 2018 field trial plot map on the Rothamsted experimental farm Appletree location. Figure S4 2018 field trial different DHAs lines DHA2015.2‐4 comparing with DHA2015.1 FAMEs. Values are mean ± SE, n = 3 Figure S5 2018 field trial different EPAs lines EPA2016.1, EPA2015.4 and EPA2015.8 comparing with EPA_B4.1 FAMEs. Values are mean ± SE, n = 3. Figure S6 Omega‐3 LC‐PUFA content in DHAs and EPAs lines of different generations. Figure S7 Single seed fatty acid composition analysis from the 2018 field trial DHA2015.1‐5 lines. Figure S8 Single seed fatty acid composition analysis from the 2018 field trial EPA2016.1, EPA2015.4 and EPA2015.8 lines. Figure S9 Analysis of triacylglycerols from DHA2015.1, DHA2015.5, EPA2016.1 and EPA2015.8 seeds of C. sativa. Figure S10 Analysis of triacylglycerols from DHA2015.1 and DHA2015.5 seeds of C. sativa. Figure S11 Analysis of triacylglycerols from EPA2016.1 and EPA2015.8 seeds of C. sativa. [file PBI-20-1833-s001.pptx]

## Slide 1
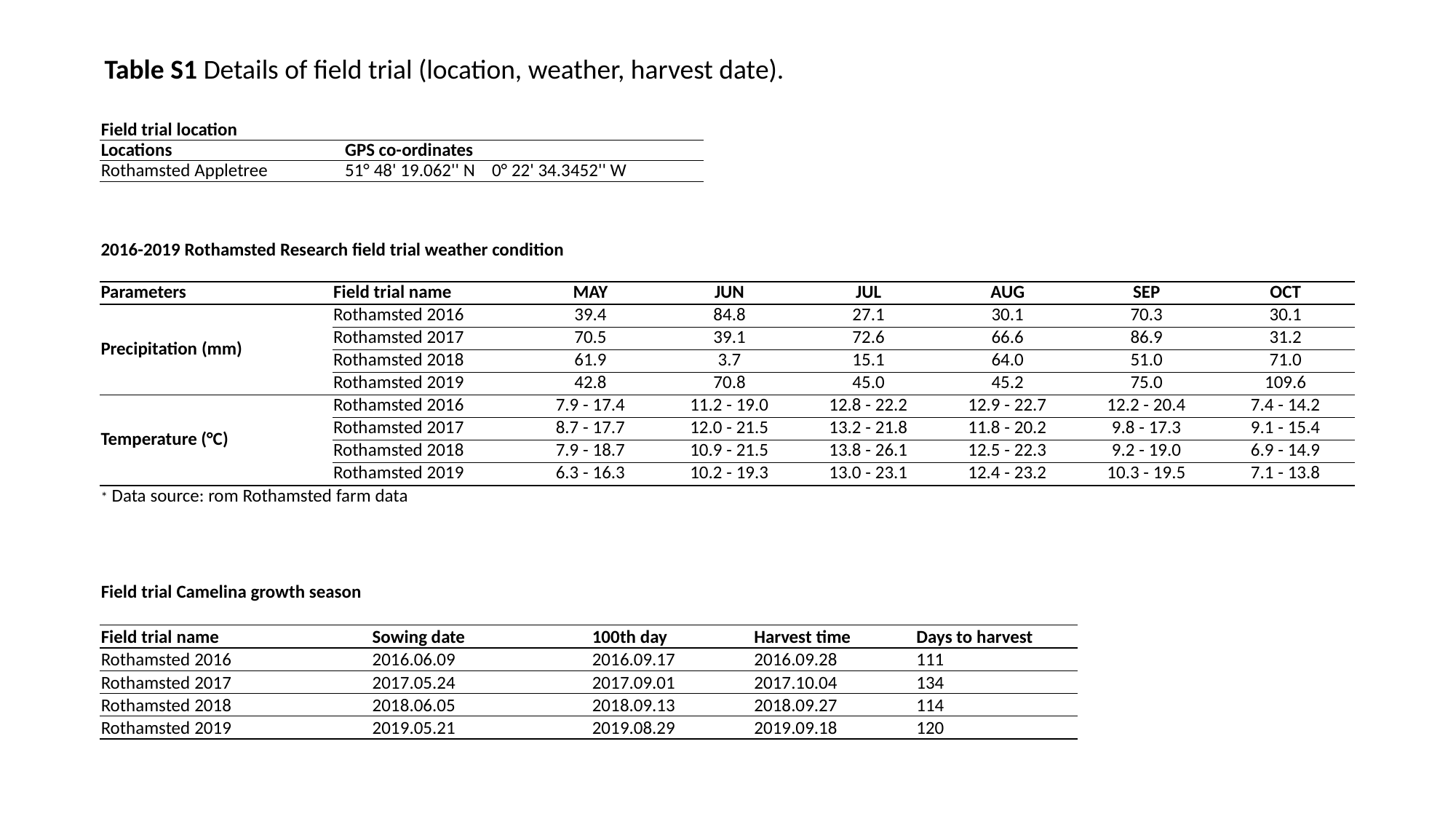

Table S1 Details of field trial (location, weather, harvest date).
| Field trial location | |
| --- | --- |
| Locations | GPS co-ordinates |
| Rothamsted Appletree | 51° 48' 19.062'' N 0° 22' 34.3452'' W |
| 2016-2019 Rothamsted Research field trial weather condition | | | | | | | |
| --- | --- | --- | --- | --- | --- | --- | --- |
| | | | | | | | |
| Parameters | Field trial name | MAY | JUN | JUL | AUG | SEP | OCT |
| Precipitation (mm) | Rothamsted 2016 | 39.4 | 84.8 | 27.1 | 30.1 | 70.3 | 30.1 |
| | Rothamsted 2017 | 70.5 | 39.1 | 72.6 | 66.6 | 86.9 | 31.2 |
| | Rothamsted 2018 | 61.9 | 3.7 | 15.1 | 64.0 | 51.0 | 71.0 |
| | Rothamsted 2019 | 42.8 | 70.8 | 45.0 | 45.2 | 75.0 | 109.6 |
| Temperature (°C) | Rothamsted 2016 | 7.9 - 17.4 | 11.2 - 19.0 | 12.8 - 22.2 | 12.9 - 22.7 | 12.2 - 20.4 | 7.4 - 14.2 |
| | Rothamsted 2017 | 8.7 - 17.7 | 12.0 - 21.5 | 13.2 - 21.8 | 11.8 - 20.2 | 9.8 - 17.3 | 9.1 - 15.4 |
| | Rothamsted 2018 | 7.9 - 18.7 | 10.9 - 21.5 | 13.8 - 26.1 | 12.5 - 22.3 | 9.2 - 19.0 | 6.9 - 14.9 |
| | Rothamsted 2019 | 6.3 - 16.3 | 10.2 - 19.3 | 13.0 - 23.1 | 12.4 - 23.2 | 10.3 - 19.5 | 7.1 - 13.8 |
| \* Data source: rom Rothamsted farm data | | | | | | | |
| Field trial Camelina growth season | | | | |
| --- | --- | --- | --- | --- |
| | | | | |
| Field trial name | Sowing date | 100th day | Harvest time | Days to harvest |
| Rothamsted 2016 | 2016.06.09 | 2016.09.17 | 2016.09.28 | 111 |
| Rothamsted 2017 | 2017.05.24 | 2017.09.01 | 2017.10.04 | 134 |
| Rothamsted 2018 | 2018.06.05 | 2018.09.13 | 2018.09.27 | 114 |
| Rothamsted 2019 | 2019.05.21 | 2019.08.29 | 2019.09.18 | 120 |

## Slide 2
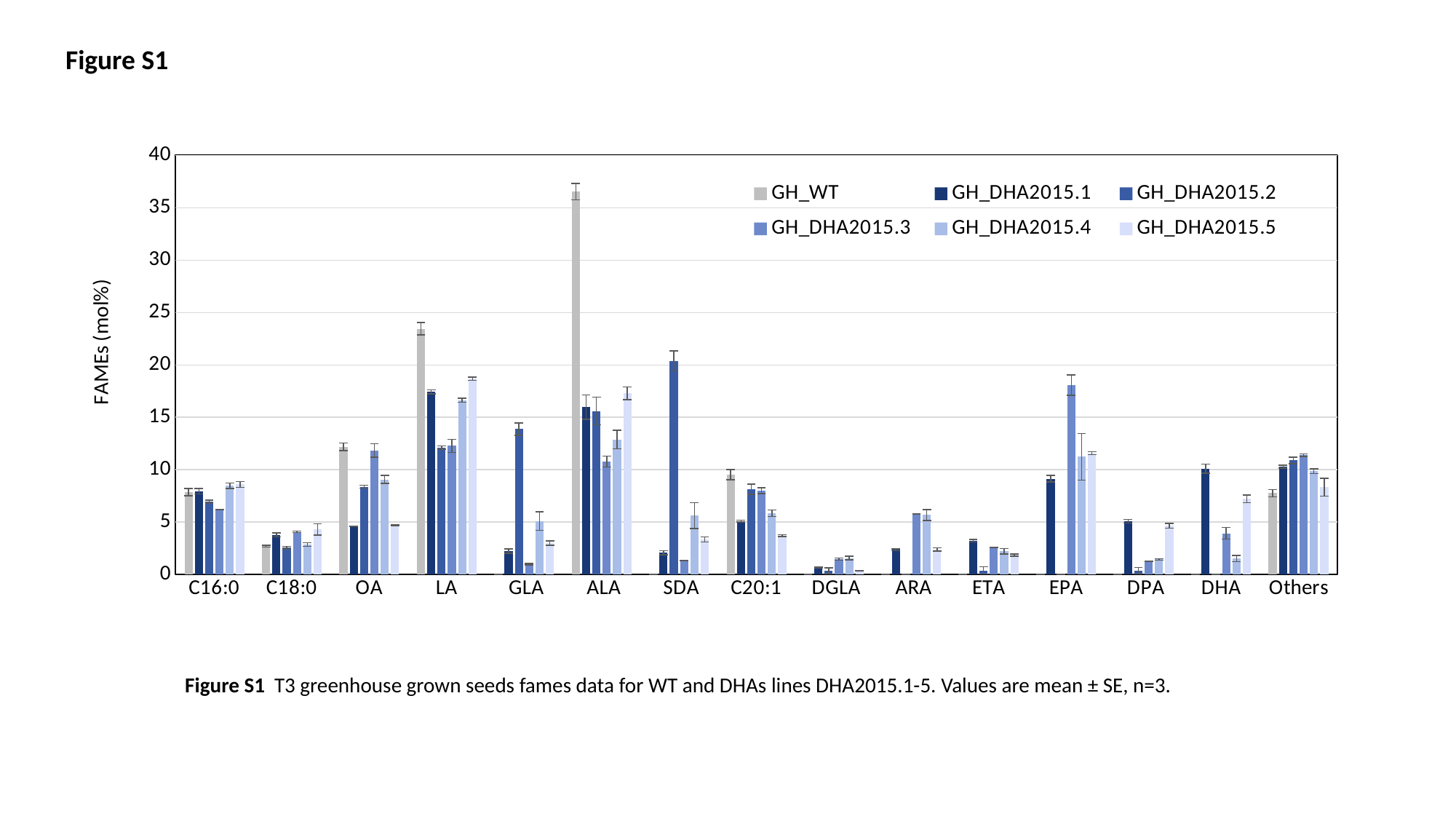

Figure S1
### Chart
| Category | GH_WT | GH_DHA2015.1 | GH_DHA2015.2 | GH_DHA2015.3 | GH_DHA2015.4 | GH_DHA2015.5 |
|---|---|---|---|---|---|---|
| C16:0 | 7.860291674646274 | 7.941095269000872 | 6.952569571066943 | 6.2025540978468 | 8.472182261798332 | 8.602366402462279 |
| C18:0 | 2.6956111089633947 | 3.7813781879781465 | 2.573021777607375 | 4.070154083383924 | 2.860524269406825 | 4.294790038951059 |
| OA | 12.17714359989237 | 4.598639162918983 | 8.38303627344381 | 11.822107536012084 | 9.069529721187157 | 4.69633496240612 |
| LA | 23.455585641736963 | 17.423539673569138 | 12.117332687350682 | 12.27400467704011 | 16.628648759582116 | 18.687372849318603 |
| GLA | 0.0 | 2.22877695705427 | 13.871223998685585 | 0.9910765843775026 | 5.090655321600358 | 3.001877772692721 |
| ALA | 36.528729484179024 | 15.974853007439096 | 15.599867200296936 | 10.790330944517777 | 12.873013821535489 | 17.304383860288798 |
| SDA | 0.0 | 2.0768219735041833 | 20.388327698174503 | 1.336257968019606 | 5.627714450583642 | 3.3461605934396133 |
| C20:1 | 9.519787123409571 | 5.097115609877194 | 8.137747710073969 | 7.9953808878908275 | 5.854120927887462 | 3.702016136040527 |
| DGLA | 0.0 | 0.666600016781592 | 0.34248556161879606 | 1.4640385845456567 | 1.574834880997206 | 0.35298055993978616 |
| ARA | 0.0 | 2.347909444039635 | 0.0 | 5.798132378378319 | 5.683159479320515 | 2.3880758328565417 |
| ETA | 0.0 | 3.2230719675339716 | 0.39322351840569986 | 2.607159058384852 | 2.209070503601691 | 1.8613335195691691 |
| EPA | 0.0 | 9.137822553428103 | 0.0 | 18.071860280161964 | 11.238361064387187 | 11.566506367353048 |
| DPA | 0.0 | 5.090719995487174 | 0.35581994876359085 | 1.264903786345759 | 1.4441509025783208 | 4.647611033028704 |
| DHA | 0.0 | 10.106394813168421 | 0.0 | 3.9266284626642904 | 1.5288929277476662 | 7.216087987430099 |
| Others | 7.762851367172405 | 10.305261368219215 | 10.885344054512109 | 11.385410670430531 | 9.84514070778604 | 8.332102084222944 |Figure S1 T3 greenhouse grown seeds fames data for WT and DHAs lines DHA2015.1-5. Values are mean ± SE, n=3.

## Slide 3
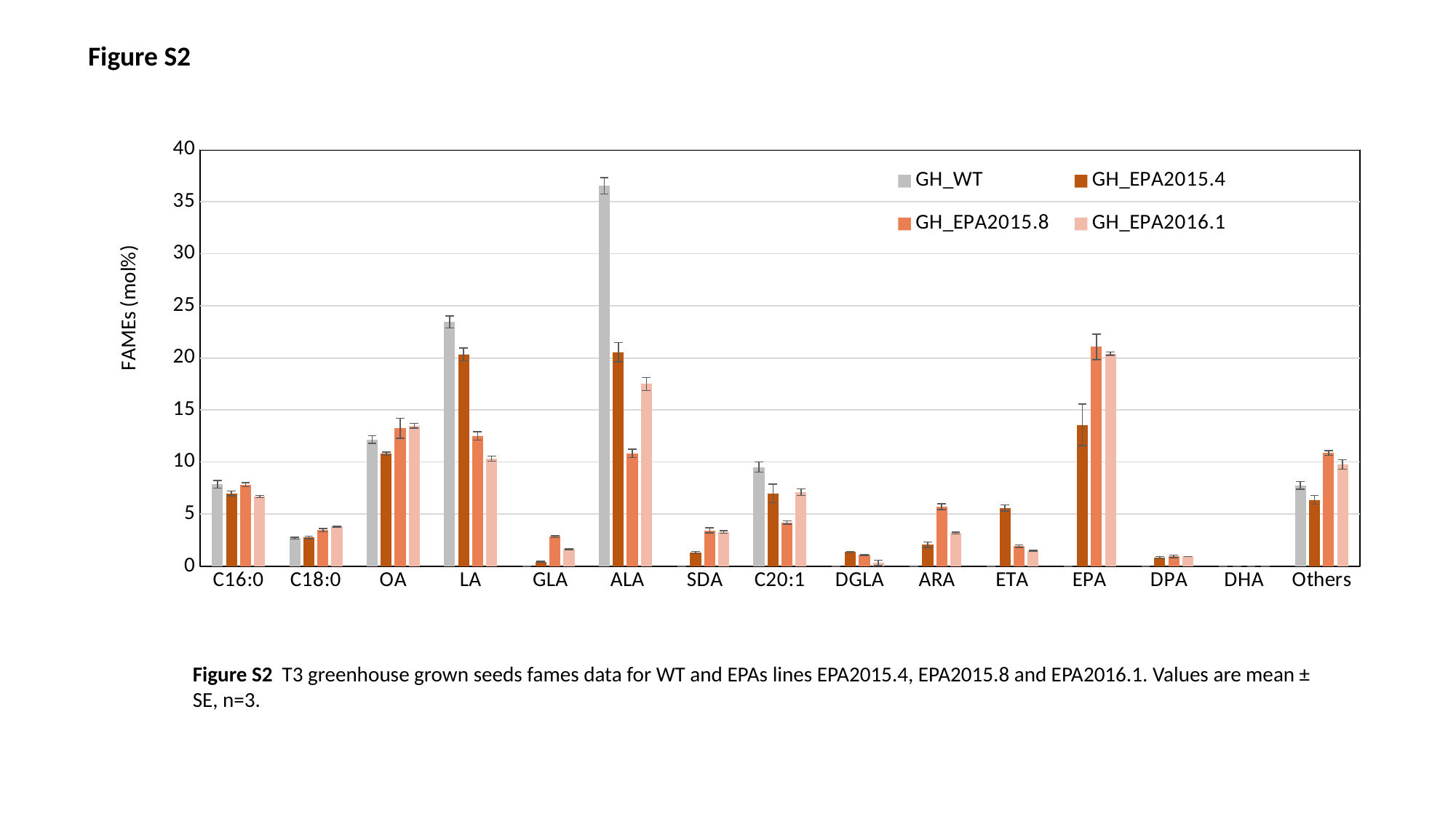

Figure S2
### Chart
| Category | GH_WT | GH_EPA2015.4 | GH_EPA2015.8 | GH_EPA2016.1 |
|---|---|---|---|---|
| C16:0 | 7.860291674646274 | 6.996239450304752 | 7.829528211310332 | 6.695394133039028 |
| C18:0 | 2.6956111089633947 | 2.7627148560629826 | 3.46898715356406 | 3.8042504920071907 |
| OA | 12.17714359989237 | 10.80911428562415 | 13.255169977500751 | 13.499096273707137 |
| LA | 23.455585641736963 | 20.339031004941845 | 12.512474174821884 | 10.341469386630465 |
| GLA | 0.0 | 0.4260761494713709 | 2.8379526137548132 | 1.6553597104471123 |
| ALA | 36.528729484179024 | 20.54530652480074 | 10.84604212259746 | 17.49863602071141 |
| SDA | 0.0 | 1.3264123847279496 | 3.445763603910665 | 3.2903283686307923 |
| C20:1 | 9.519787123409571 | 7.001651837044737 | 4.202349828129672 | 7.116396899640637 |
| DGLA | 0.0 | 1.3627073989960554 | 1.0724928671871623 | 0.3029907785781133 |
| ARA | 0.0 | 2.0668959534519638 | 5.7121632222831344 | 3.195660611337813 |
| ETA | 0.0 | 5.580082420010893 | 1.9157588429341514 | 1.4772948588564498 |
| EPA | 0.0 | 13.577693853951823 | 21.07631682178209 | 20.41721011580441 |
| DPA | 0.0 | 0.8304335260382305 | 0.9437081312698513 | 0.9327853610949085 |
| DHA | 0.0 | 0.0 | 0.0 | 0.0 |
| Others | 7.762851367172405 | 6.375640354572496 | 10.881292428953984 | 9.773126989514532 |Figure S2 T3 greenhouse grown seeds fames data for WT and EPAs lines EPA2015.4, EPA2015.8 and EPA2016.1. Values are mean ± SE, n=3.

## Slide 4
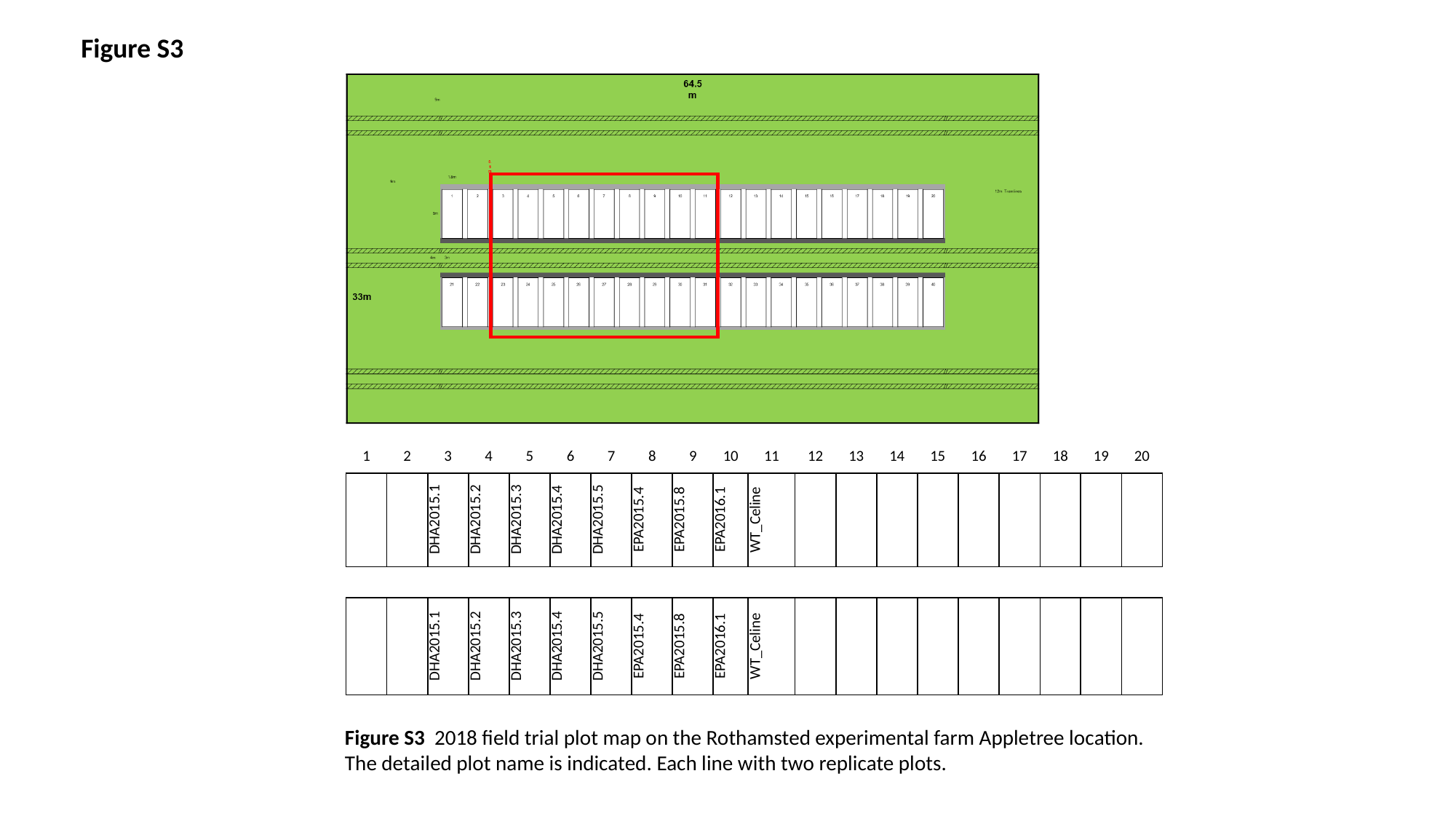

Figure S3
| 1 | 2 | 3 | 4 | 5 | 6 | 7 | 8 | 9 | 10 | 11 | 12 | 13 | 14 | 15 | 16 | 17 | 18 | 19 | 20 |
| --- | --- | --- | --- | --- | --- | --- | --- | --- | --- | --- | --- | --- | --- | --- | --- | --- | --- | --- | --- |
| | | DHA2015.1 | DHA2015.2 | DHA2015.3 | DHA2015.4 | DHA2015.5 | EPA2015.4 | EPA2015.8 | EPA2016.1 | WT\_Celine | | | | | | | | | |
| | | | | | | | | | | | | | | | | | | | |
| | | DHA2015.1 | DHA2015.2 | DHA2015.3 | DHA2015.4 | DHA2015.5 | EPA2015.4 | EPA2015.8 | EPA2016.1 | WT\_Celine | | | | | | | | | |
Figure S3 2018 field trial plot map on the Rothamsted experimental farm Appletree location. The detailed plot name is indicated. Each line with two replicate plots.

## Slide 5
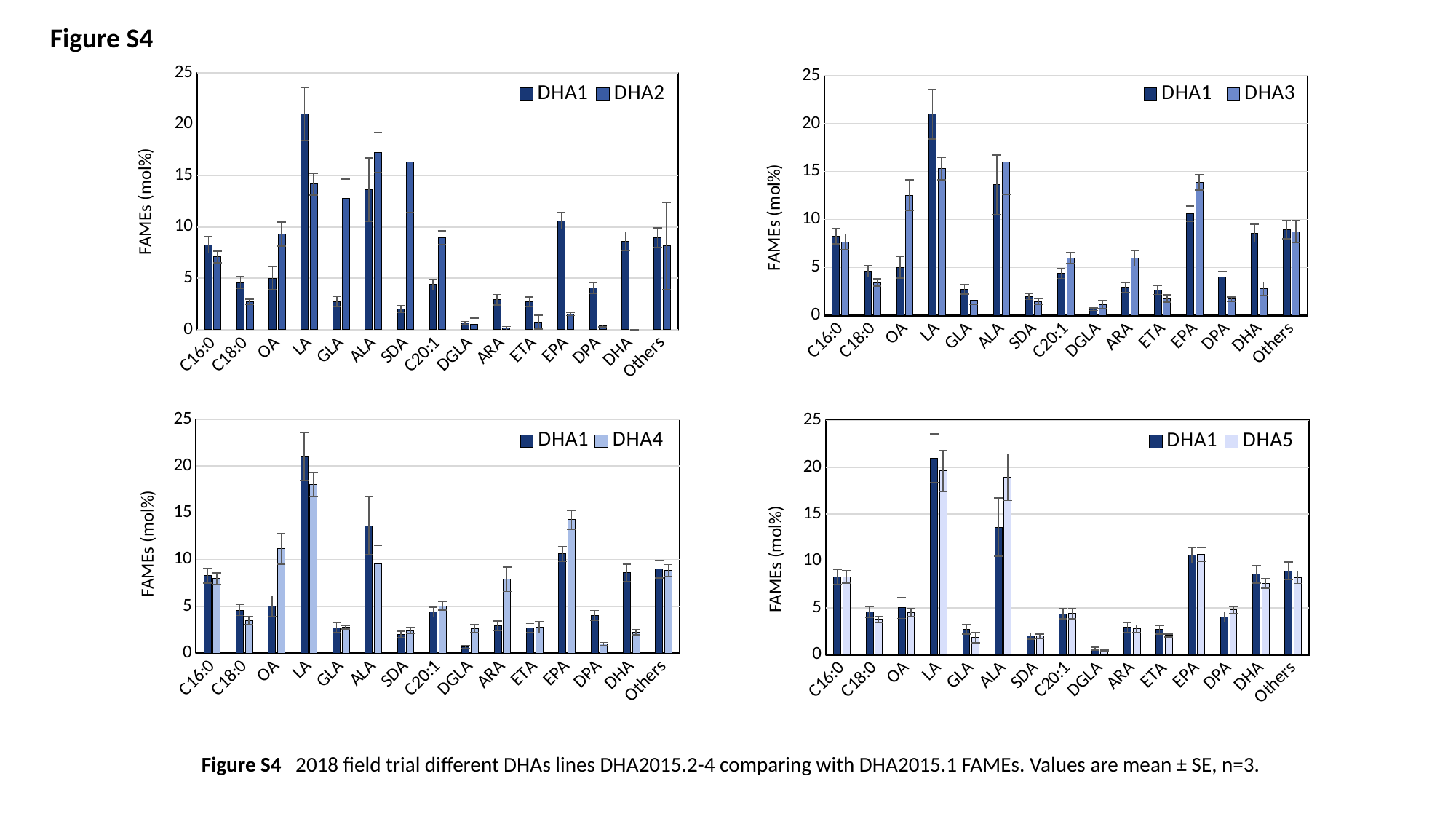

Figure S4
### Chart
| Category | DHA1 | DHA2 |
|---|---|---|
| C16:0 | 8.27101443979725 | 7.0793085696414755 |
| C18:0 | 4.5881471674217345 | 2.706589419747264 |
| OA | 5.0036967655752855 | 9.309691078558865 |
| LA | 20.986278449836853 | 14.172030366225865 |
| GLA | 2.722528481403675 | 12.75744320945615 |
| ALA | 13.615698491926382 | 17.256575193789605 |
| SDA | 1.990290051169313 | 16.35528761427963 |
| C20:1 | 4.3843471499260485 | 8.962517503353808 |
| DGLA | 0.6526794568911062 | 0.5239815440814423 |
| ARA | 2.9245015805479837 | 0.13693390487182938 |
| ETA | 2.6839962456606936 | 0.7200021452786131 |
| EPA | 10.60540622320818 | 1.516827104403281 |
| DPA | 4.035413337237228 | 0.36503088649721427 |
| DHA | 8.580345482551547 | 0.0 |
| Others | 8.955656676846717 | 8.137781459814954 |
### Chart
| Category | DHA1 | DHA3 |
|---|---|---|
| C16:0 | 8.27101443979725 | 7.679132910114306 |
| C18:0 | 4.5881471674217345 | 3.437965863325861 |
| OA | 5.0036967655752855 | 12.53408160871093 |
| LA | 20.986278449836853 | 15.308952071256133 |
| GLA | 2.722528481403675 | 1.6132857078796552 |
| ALA | 13.615698491926382 | 15.979143159326266 |
| SDA | 1.990290051169313 | 1.4681994892529926 |
| C20:1 | 4.3843471499260485 | 5.97518644778651 |
| DGLA | 0.6526794568911062 | 1.1588089810478919 |
| ARA | 2.9245015805479837 | 5.960847274838872 |
| ETA | 2.6839962456606936 | 1.7765229410831975 |
| EPA | 10.60540622320818 | 13.873845782221844 |
| DPA | 4.035413337237228 | 1.7039042795034607 |
| DHA | 8.580345482551547 | 2.788395890828082 |
| Others | 8.955656676846717 | 8.741727592824015 |
### Chart
| Category | DHA1 | DHA4 |
|---|---|---|
| C16:0 | 8.27101443979725 | 7.957295863267556 |
| C18:0 | 4.5881471674217345 | 3.5038174849052846 |
| OA | 5.0036967655752855 | 11.140024585788801 |
| LA | 20.986278449836853 | 18.023400382104764 |
| GLA | 2.722528481403675 | 2.780813719256257 |
| ALA | 13.615698491926382 | 9.552249509309945 |
| SDA | 1.990290051169313 | 2.4071747318186096 |
| C20:1 | 4.3843471499260485 | 5.0527089837695645 |
| DGLA | 0.6526794568911062 | 2.6346693178920932 |
| ARA | 2.9245015805479837 | 7.8803213819172555 |
| ETA | 2.6839962456606936 | 2.756886161767213 |
| EPA | 10.60540622320818 | 14.260406264357133 |
| DPA | 4.035413337237228 | 0.9866335427108445 |
| DHA | 8.580345482551547 | 2.25142781791977 |
| Others | 8.955656676846717 | 8.812170253214905 |
### Chart
| Category | DHA1 | DHA5 |
|---|---|---|
| C16:0 | 8.27101443979725 | 8.301491579129161 |
| C18:0 | 4.5881471674217345 | 3.7755643589203682 |
| OA | 5.0036967655752855 | 4.521178005658003 |
| LA | 20.986278449836853 | 19.600421391331505 |
| GLA | 2.722528481403675 | 1.8378755057045604 |
| ALA | 13.615698491926382 | 18.939242099638463 |
| SDA | 1.990290051169313 | 1.9806754801512119 |
| C20:1 | 4.3843471499260485 | 4.399777745967867 |
| DGLA | 0.6526794568911062 | 0.4500658347404415 |
| ARA | 2.9245015805479837 | 2.767686670343907 |
| ETA | 2.6839962456606936 | 2.0693977267758723 |
| EPA | 10.60540622320818 | 10.688852504645523 |
| DPA | 4.035413337237228 | 4.788279288052416 |
| DHA | 8.580345482551547 | 7.617961910966196 |
| Others | 8.955656676846717 | 8.261529897974508 |Figure S4 2018 field trial different DHAs lines DHA2015.2-4 comparing with DHA2015.1 FAMEs. Values are mean ± SE, n=3.

## Slide 6
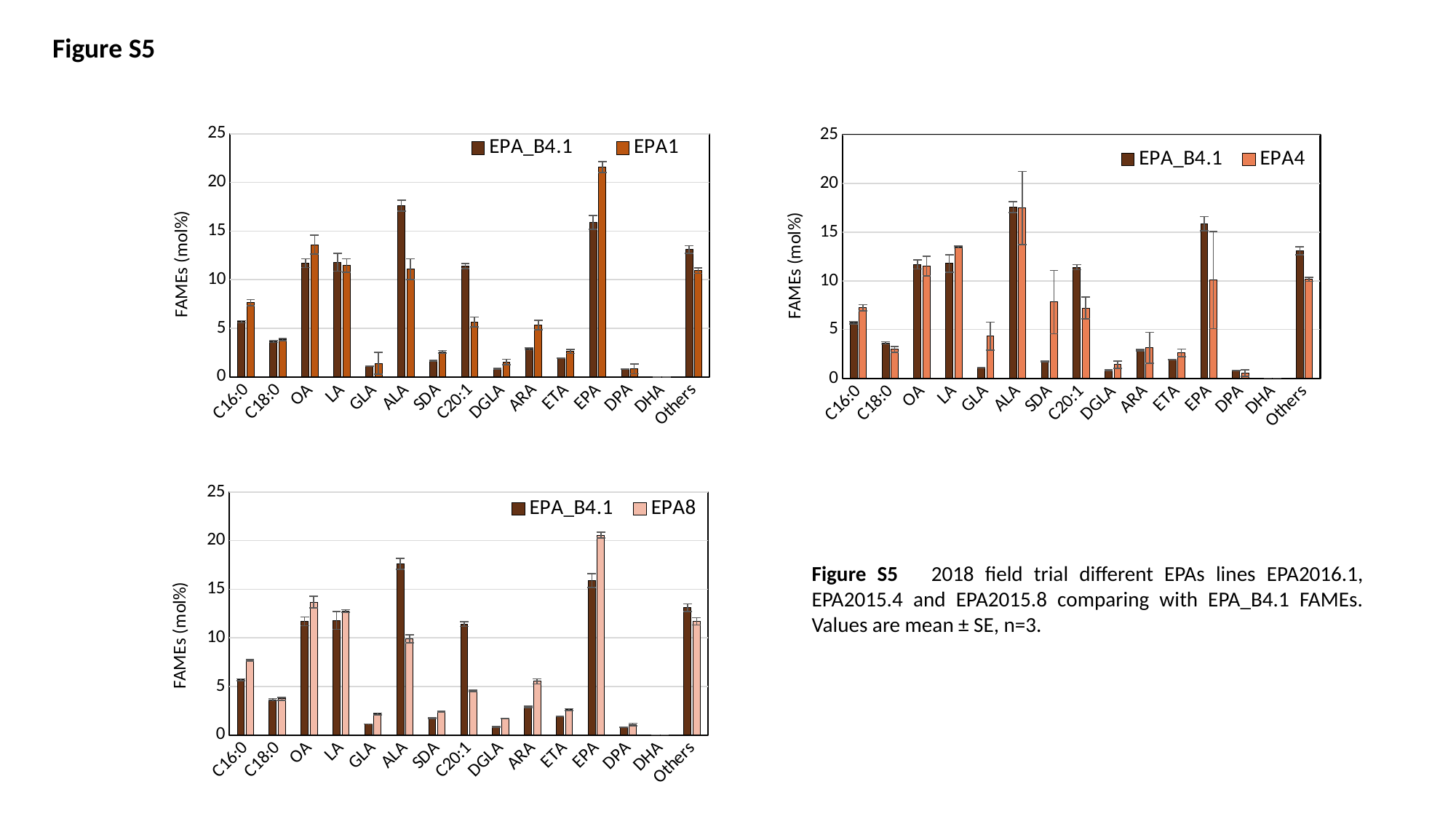

Figure S5
### Chart
| Category | EPA_B4.1 | EPA1 |
|---|---|---|
| C16:0 | 5.674012986698443 | 7.618716919097213 |
| C18:0 | 3.626323386359251 | 3.8294484570249456 |
| OA | 11.69869897371593 | 13.598146248373373 |
| LA | 11.790007256574512 | 11.45462168132083 |
| GLA | 1.1170779874656427 | 1.3946619579847177 |
| ALA | 17.59776349896926 | 11.07599092876257 |
| SDA | 1.6969078571079723 | 2.585032562083869 |
| C20:1 | 11.407326916344738 | 5.6372099165591925 |
| DGLA | 0.8279362366603403 | 1.5260505403269147 |
| ARA | 2.8770976231695897 | 5.336096924366786 |
| ETA | 1.9110822176934665 | 2.609684383063934 |
| EPA | 15.883233796640171 | 21.58133769203808 |
| DPA | 0.7941050693711973 | 0.8207416080670799 |
| DHA | 0.0 | 0.0 |
| Others | 13.098426193229486 | 10.932260180930525 |
### Chart
| Category | EPA_B4.1 | EPA4 |
|---|---|---|
| C16:0 | 5.674012986698443 | 7.242704183530466 |
| C18:0 | 3.626323386359251 | 2.9709323004223975 |
| OA | 11.69869897371593 | 11.511948663508567 |
| LA | 11.790007256574512 | 13.494231282928547 |
| GLA | 1.1170779874656427 | 4.336613297553739 |
| ALA | 17.59776349896926 | 17.49242438167322 |
| SDA | 1.6969078571079723 | 7.820765803979604 |
| C20:1 | 11.407326916344738 | 7.209384529667704 |
| DGLA | 0.8279362366603403 | 1.3923234765550603 |
| ARA | 2.8770976231695897 | 3.1282259596234243 |
| ETA | 1.9110822176934665 | 2.614036975602248 |
| EPA | 15.883233796640171 | 10.084583930695883 |
| DPA | 0.7941050693711973 | 0.5278475800810681 |
| DHA | 0.0 | 0.0 |
| Others | 13.098426193229486 | 10.173977634178078 |
### Chart
| Category | EPA_B4.1 | EPA8 |
|---|---|---|
| C16:0 | 5.674012986698443 | 7.672231425956203 |
| C18:0 | 3.626323386359251 | 3.7173536791101047 |
| OA | 11.69869897371593 | 13.673868873574937 |
| LA | 11.790007256574512 | 12.761716290515594 |
| GLA | 1.1170779874656427 | 2.164008302605737 |
| ALA | 17.59776349896926 | 9.907773207236971 |
| SDA | 1.6969078571079723 | 2.4153998593679114 |
| C20:1 | 11.407326916344738 | 4.543593177640921 |
| DGLA | 0.8279362366603403 | 1.670151221065475 |
| ARA | 2.8770976231695897 | 5.519626934025898 |
| ETA | 1.9110822176934665 | 2.6172246694103016 |
| EPA | 15.883233796640171 | 20.57151512611533 |
| DPA | 0.7941050693711973 | 1.068212608304248 |
| DHA | 0.0 | 0.0 |
| Others | 13.098426193229486 | 11.697324625070365 |Figure S5 2018 field trial different EPAs lines EPA2016.1, EPA2015.4 and EPA2015.8 comparing with EPA_B4.1 FAMEs. Values are mean ± SE, n=3.

## Slide 7
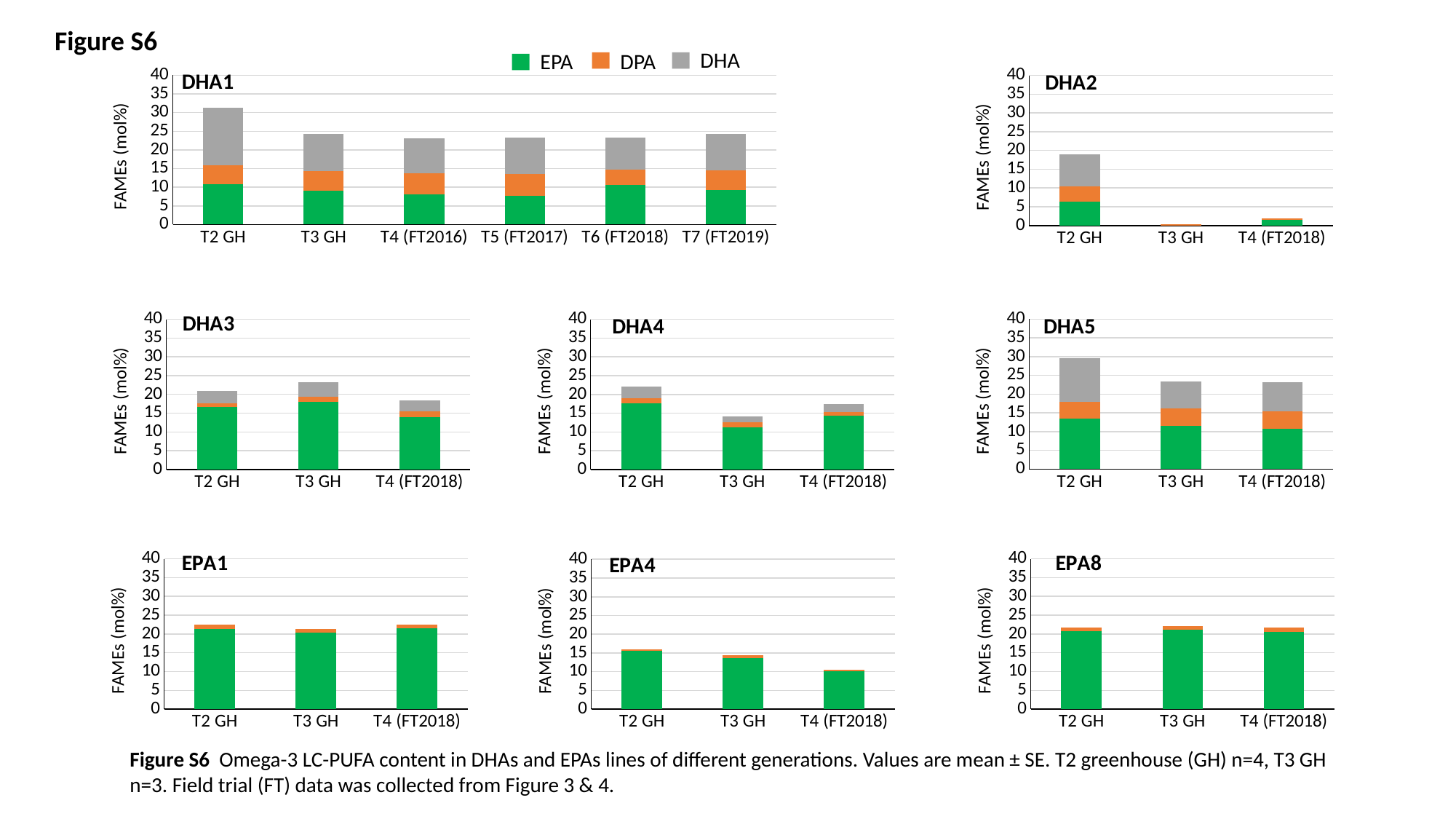

### Chart: DHA1
| Category | EPA | DPA | DHA |
|---|---|---|---|
| T2 GH | 10.76544163164687 | 5.190894298939237 | 15.383350210809988 |
| T3 GH | 9.137822553428103 | 5.090719995487174 | 10.106394813168421 |
| T4 (FT2016) | 7.9843069712488255 | 5.730486737011482 | 9.34665818458698 |
| T5 (FT2017) | 7.6948014278559915 | 5.846313316300204 | 9.752672838525356 |
| T6 (FT2018) | 10.60540622320818 | 4.035413337237228 | 8.580345482551547 |
| T7 (FT2019) | 9.31331440284454 | 5.207422388347583 | 9.703887045563725 |
### Chart: DHA2
| Category | EPA | DPA | DHA |
|---|---|---|---|
| T2 GH | 6.377822404130394 | 4.101447098233317 | 8.565784218119745 |
| T3 GH | 0.0 | 0.35581994876359085 | 0.0 |
| T4 (FT2018) | 1.516827104403281 | 0.36503088649721427 | 0.0 |Figure S6
DHA
EPA
DPA
### Chart: DHA3
| Category | EPA | DPA | DHA |
|---|---|---|---|
| T2 GH | 16.695066053101236 | 0.8351609530951716 | 3.4500398822543374 |
| T3 GH | 18.071860280161964 | 1.264903786345759 | 3.9266284626642904 |
| T4 (FT2018) | 13.873845782221844 | 1.7039042795034607 | 2.788395890828082 |
### Chart: DHA4
| Category | EPA | DPA | DHA |
|---|---|---|---|
| T2 GH | 17.56753432418976 | 1.3546211914853816 | 3.135747980811587 |
| T3 GH | 11.238361064387187 | 1.4441509025783208 | 1.5288929277476662 |
| T4 (FT2018) | 14.260406264357133 | 0.9866335427108445 | 2.25142781791977 |
### Chart: DHA5
| Category | EPA | DPA | DHA |
|---|---|---|---|
| T2 GH | 13.479210034933493 | 4.412209494944363 | 11.695850023359222 |
| T3 GH | 11.566506367353048 | 4.647611033028704 | 7.216087987430099 |
| T4 (FT2018) | 10.688852504645523 | 4.788279288052416 | 7.617961910966196 |
### Chart: EPA1
| Category | EPA | DPA | DHA |
|---|---|---|---|
| T2 GH | 21.311163860961603 | 1.1237649825734066 | 0.0 |
| T3 GH | 20.41721011580441 | 0.9327853610949085 | 0.0 |
| T4 (FT2018) | 21.58133769203808 | 0.8207416080670799 | 0.0 |
### Chart: EPA8
| Category | EPA | DPA | DHA |
|---|---|---|---|
| T2 GH | 20.82737177063776 | 0.8611759694232415 | 0.0 |
| T3 GH | 21.07631682178209 | 0.9437081312698513 | 0.0 |
| T4 (FT2018) | 20.57151512611533 | 1.068212608304248 | 0.0 |
### Chart: EPA4
| Category | EPA | DPA | DHA |
|---|---|---|---|
| T2 GH | 15.526640894843739 | 0.490408233938489 | 0.0 |
| T3 GH | 13.577693853951823 | 0.8304335260382305 | 0.0 |
| T4 (FT2018) | 10.084583930695883 | 0.5278475800810681 | 0.0 |Figure S6 Omega-3 LC-PUFA content in DHAs and EPAs lines of different generations. Values are mean ± SE. T2 greenhouse (GH) n=4, T3 GH n=3. Field trial (FT) data was collected from Figure 3 & 4.

## Slide 8
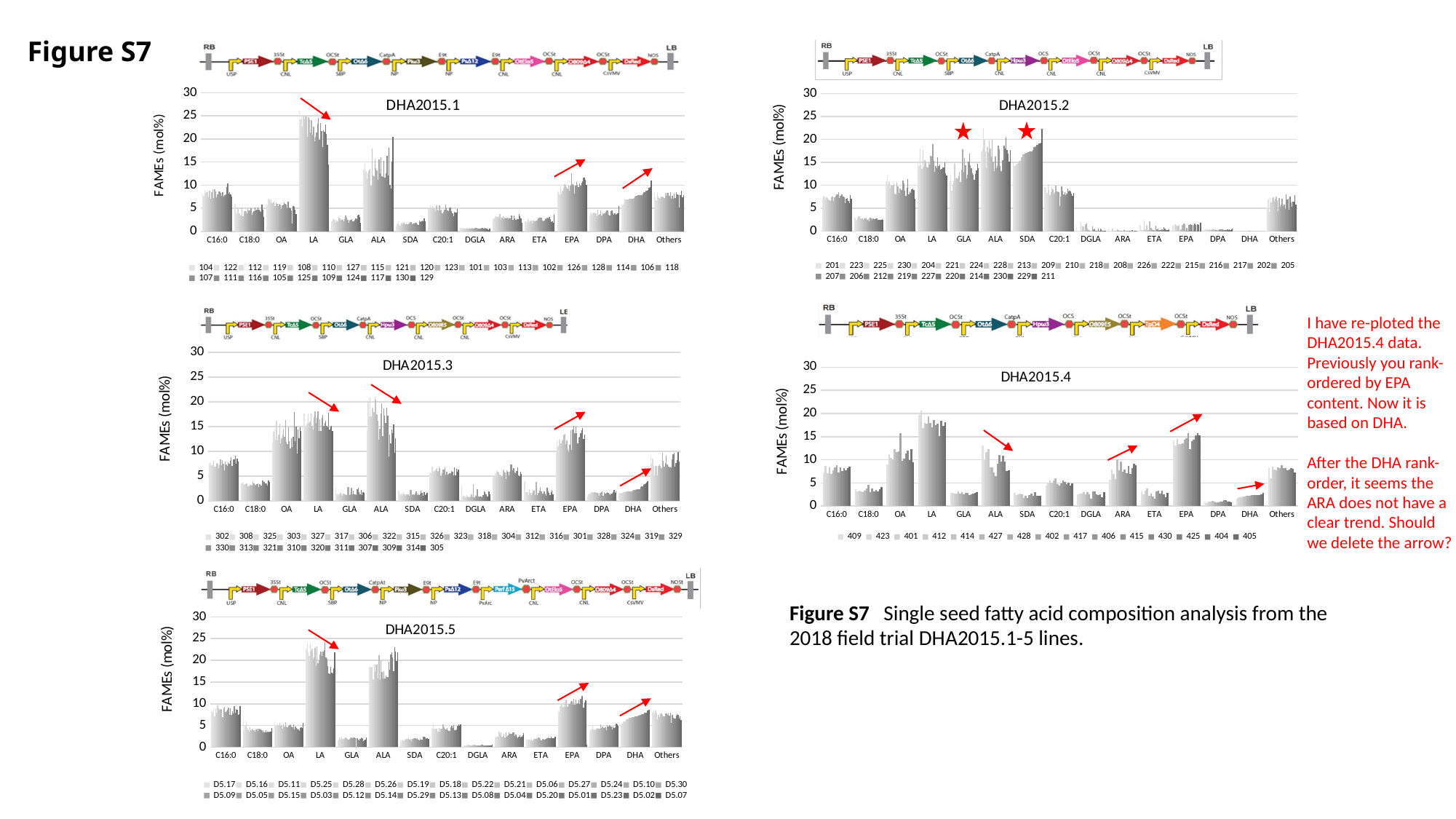

Figure S7
### Chart: DHA2015.1
| Category | 104 | 122 | 112 | 119 | 108 | 110 | 127 | 115 | 121 | 120 | 123 | 101 | 103 | 113 | 102 | 126 | 128 | 114 | 106 | 118 | 107 | 111 | 116 | 105 | 125 | 109 | 124 | 117 | 130 | 129 |
|---|---|---|---|---|---|---|---|---|---|---|---|---|---|---|---|---|---|---|---|---|---|---|---|---|---|---|---|---|---|---|
| C16:0 | 8.112585320697207 | 7.677888686629922 | 8.72711750651899 | 8.407010760783276 | 8.563407106455331 | 8.668198816135195 | 7.5936495608155585 | 8.752727480124223 | 7.020231413732527 | 8.47169294622444 | 9.169981361106718 | 7.248649892894703 | 9.085712888289262 | 8.671863070777999 | 7.351728632776736 | 8.018589328546136 | 8.57218712464741 | 8.602361473670632 | 8.271705723127114 | 7.747748457997415 | 8.539111549698404 | 7.578811348554666 | 7.839779104104651 | 7.9629996340111635 | 9.529200692047212 | 10.393881094282685 | 8.16710687099127 | 8.51461661833394 | 8.067829587324871 | 7.509920830515072 |
| C18:0 | 6.024981948698278 | 4.268210179964067 | 3.8641811376214994 | 4.853654775814871 | 3.9712956336905267 | 3.9650701674988986 | 3.4814189066659456 | 5.128613342947397 | 3.342707868266341 | 4.441378288555522 | 4.40775388041261 | 4.3471241066994555 | 3.9700833421993966 | 4.922638730385468 | 4.491146003282812 | 4.565464831609198 | 5.015678538673452 | 3.646768719747161 | 4.091006941008004 | 4.353138304760928 | 5.198730896026994 | 4.526452516658985 | 4.622969799570729 | 5.1607744749890765 | 4.636114667079933 | 4.393313627507753 | 4.151850829077761 | 5.740163473879504 | 4.705361102443759 | 3.170041504757132 |
| OA | 5.599860778965251 | 7.013546543924242 | 6.970135991274973 | 6.460555205322291 | 6.964957765705411 | 6.0549537073662165 | 6.306606837950744 | 6.4937613752815695 | 5.52535529045311 | 6.301918322705995 | 5.924940538697396 | 5.454367086732262 | 5.914445218466368 | 5.846648923089108 | 4.851235502346759 | 5.647141305264967 | 5.74515595617307 | 6.313689184308394 | 6.017671104746471 | 5.8243875185737375 | 4.96317773644476 | 6.463212913026674 | 5.136847035473987 | 4.955272790472276 | 4.513707839064815 | 1.7349983153653719 | 5.418902424473258 | 5.2607203730811385 | 4.758533418519805 | 3.7644872506022056 |
| LA | 26.075171021842415 | 24.178725167939973 | 25.255769530680585 | 22.798891512900518 | 25.133600524500814 | 24.332119273236632 | 25.072534196433388 | 24.867702206248634 | 20.392065796017352 | 24.709740543883058 | 24.271163075163297 | 21.45995294805997 | 24.01953475941288 | 20.928268656512305 | 22.74514073719882 | 19.57754087743586 | 20.499888742605588 | 21.427065062016535 | 23.24290861561354 | 24.585033026380728 | 19.755576914286046 | 23.530003320746232 | 21.779934455734093 | 18.330703100174375 | 21.904784721633458 | 21.520387930824104 | 23.1030895900528 | 21.055351138060754 | 18.758576415601514 | 14.405257161204876 |
| GLA | 2.2315813003487532 | 2.621929410821174 | 2.7671513474587486 | 2.556612118084895 | 1.8476368517001283 | 2.530714919420912 | 2.156538442278286 | 3.147227065540414 | 2.794577239688725 | 2.314981931612568 | 2.6062304144896538 | 2.5250765270185767 | 2.2466376899324003 | 2.6809757193548975 | 3.507483276999697 | 2.898065874416817 | 2.495805268556616 | 1.807454849025598 | 2.359813460067885 | 2.552451434285676 | 2.5785696062630445 | 2.243692002970155 | 2.1278066880064124 | 2.476410680947433 | 2.8372006651496284 | 2.7035044149976253 | 3.451186557373987 | 3.525576878866849 | 3.155273710777136 | 1.9250867013761663 |
| ALA | 13.353351009885678 | 14.854232857498442 | 12.98711503437301 | 11.507476505983682 | 12.63246896191879 | 13.173346393566657 | 13.52589544162097 | 9.961314201856412 | 17.884302552798093 | 12.137245274397841 | 12.021148781196542 | 15.563290823873135 | 13.656444435996598 | 13.231151093969046 | 11.145604764411566 | 15.644154989778908 | 12.660426166018958 | 15.981494650921206 | 11.899230636534325 | 11.850021806727728 | 15.302807262966926 | 11.67713379611122 | 12.654137300941287 | 16.3302295413174 | 12.04882700789882 | 18.08903522011234 | 10.05242331188715 | 9.337089612059955 | 15.04369662609541 | 20.50065933603376 |
| SDA | 1.224399755776041 | 1.69899371915298 | 2.148874878425709 | 1.5093616410004183 | 1.2707119311770134 | 1.8752770692162977 | 1.665456325431957 | 1.8737936189139606 | 2.0822259740456777 | 1.4972206709768539 | 1.8197226013067138 | 1.5975779016535745 | 1.595274590584134 | 1.681392514083984 | 2.0498410198075265 | 2.0212260225716205 | 1.617162179172386 | 1.6508076710481736 | 1.6472946553488776 | 1.8692498102179669 | 1.907636106704556 | 1.5820338440660626 | 1.431761591166549 | 2.260647009632141 | 1.9501280652331143 | 2.2286256763691688 | 2.270133470715153 | 2.1441041926360866 | 2.7386013178345587 | 2.135905806063925 |
| C20:1 | 4.789173836578494 | 4.908779126111369 | 5.639712931406072 | 4.954671568736694 | 5.501456582490487 | 4.92600015002688 | 4.930624558861716 | 4.446196839127885 | 5.598833434305503 | 4.743375920262822 | 4.474815240186507 | 5.587820929758943 | 4.581763987283456 | 4.62806641903436 | 3.9416250035352256 | 4.163188561911256 | 4.751412073360836 | 5.872281116405557 | 5.0675782449061675 | 4.4890910020942965 | 4.353480739031335 | 5.0612595425038105 | 5.1995613960633325 | 4.395705643566181 | 3.8960002976215535 | 3.312999614572648 | 4.240028087790639 | 3.9905989802751316 | 4.047156664758334 | 4.855521396899982 |
| DGLA | 0.6806162971176065 | 0.6850545789680886 | 0.626966296079221 | 0.793590611425087 | 0.6674200929196296 | 0.6067696642961445 | 0.6849588178183322 | 0.5251589982118358 | 0.6403868492798497 | 0.6800910247120088 | 0.6192329808672005 | 0.7039605663438584 | 0.6783306019600868 | 0.6755058266112485 | 0.8115174209227201 | 0.735542751992982 | 0.7368136458404264 | 0.585175321910526 | 0.6486931719964912 | 0.6616038999616123 | 0.6572313967196538 | 0.8090353900015471 | 0.8355986388089369 | 0.588355812741882 | 0.8282694211518987 | 0.5994991130658833 | 0.522089102192192 | 0.4815051258585576 | 0.34563744723206685 | 0.5287995148797439 |
| ARA | 2.7104338894003672 | 2.8835771564862562 | 3.298406362821896 | 3.29842414227141 | 3.1615111879381033 | 3.1575555814045657 | 3.121264694150084 | 3.688362460714163 | 2.529052292956566 | 3.0403650253789816 | 3.2745642079871 | 2.640460129386582 | 2.9227082921841507 | 2.7466558967612436 | 2.9641096695436184 | 2.656952923544861 | 2.9012049128951087 | 2.813127237648782 | 3.4787070249065892 | 3.498263637540853 | 2.445977769511519 | 3.4658055342200367 | 2.811273879639205 | 2.4382785481358322 | 2.7037549149275466 | 2.6922246921652615 | 3.815540216912657 | 3.2687479046498766 | 2.877423979965404 | 1.8492580996613832 |
| ETA | 2.194722733783896 | 2.101415691425122 | 1.783503279808208 | 2.5904339194478743 | 2.052612079666627 | 2.142919186534861 | 2.2827591918098493 | 1.7213973028765062 | 2.343880035540212 | 2.224645490275601 | 2.1762163403023633 | 2.291670276871883 | 2.4794476734810753 | 2.7297799875230595 | 3.0025613748950986 | 2.8946821634256166 | 2.925514788125689 | 2.313055661326186 | 2.238414082554632 | 2.3664795091681246 | 2.826995917304917 | 2.6532432303908458 | 2.9778328950109487 | 2.866202496486788 | 3.163447641609038 | 2.6050432085078046 | 2.017915577823453 | 2.2076458661135465 | 1.8643607620525429 | 3.6496041714413603 |
| EPA | 8.4429417929841 | 8.584487842518216 | 7.9446427212638175 | 9.760189026475713 | 8.110808710792318 | 8.770196978844297 | 9.481803155310109 | 10.226495966063105 | 9.886226156968574 | 9.601663239775432 | 9.343307570146985 | 9.831386753832644 | 8.843529245715366 | 9.844665513637842 | 12.541038601833028 | 10.226238513290003 | 9.944864931303828 | 8.230516119353767 | 9.860756003540793 | 10.670068258566273 | 10.13609321618196 | 9.626741584946092 | 10.365008620868544 | 9.91346736884049 | 10.602476307403144 | 10.786509229078826 | 11.568234815736673 | 11.631384608587158 | 11.125554979877002 | 10.0826563080689 |
| DPA | 3.5125849724734595 | 4.079406278022351 | 3.569015062113646 | 3.9581768030702356 | 4.111637225248947 | 3.8564511636582934 | 3.9509278212696755 | 3.2433826393840084 | 4.679921570330677 | 3.5877203285039774 | 3.749538841679713 | 4.511909762057254 | 3.3857925910113993 | 3.940942786257601 | 3.7347252520304934 | 3.930658431157831 | 4.269069902270684 | 4.529358763308495 | 4.592085812478898 | 3.6186829654384534 | 3.472327742252053 | 4.430662039333425 | 4.486592988805261 | 3.844019115820476 | 3.54484083196226 | 3.837984928086309 | 3.6354695923485103 | 3.6788701375125217 | 3.960756648298774 | 5.494453670762456 |
| DHA | 5.731420554146632 | 5.735649024096134 | 6.162930735671221 | 6.904511453552473 | 6.940326561802849 | 6.96269362049695 | 6.980131817894509 | 7.0218049377160945 | 7.065077710496012 | 7.066596794437357 | 7.121082914151384 | 7.137693108400722 | 7.242897061297185 | 7.539869482327959 | 7.624013064698972 | 7.771317342452148 | 7.785052004985094 | 7.8073733750583685 | 7.887289760390275 | 7.898154496489637 | 7.905826648847334 | 8.161645965026304 | 8.534683688426476 | 8.548702388313295 | 8.754725770891413 | 8.80688626652972 | 8.982213705484172 | 9.454203309659166 | 9.517263907563459 | 11.07320391851574 |
| Others | 8.552177823887103 | 7.404180800745322 | 6.678109454526906 | 8.311504511725682 | 7.504089154995933 | 7.16244142044992 | 7.132369868645021 | 7.547339851985612 | 7.443939651406691 | 7.612044551693705 | 7.249766773109008 | 8.26132934558335 | 7.691221331000406 | 8.369413592909092 | 8.326639027673526 | 7.6352144585399095 | 8.480689678109254 | 7.035128744651808 | 7.676010131809245 | 7.115076957257476 | 8.157668033900059 | 7.287079062420675 | 8.467006109856367 | 7.9574223721893995 | 7.8807907878042895 | 5.1607552128200815 | 7.831089464361179 | 8.764712270334023 | 7.341115067192027 | 7.896104281963925 |
### Chart: DHA2015.2
| Category | 201 | 223 | 225 | 230 | 204 | 221 | 224 | 228 | 213 | 209 | 210 | 218 | 208 | 226 | 222 | 215 | 216 | 217 | 202 | 205 | 207 | 206 | 212 | 219 | 227 | 220 | 214 | 230 | 229 | 211 |
|---|---|---|---|---|---|---|---|---|---|---|---|---|---|---|---|---|---|---|---|---|---|---|---|---|---|---|---|---|---|---|
| C16:0 | 7.142352434105468 | 7.681296417269372 | 7.594007419446023 | 6.958157863340566 | 7.416662296116183 | 7.288887338434336 | 6.906388941984903 | 6.7802167656510015 | 6.549467934057799 | 7.46839208426174 | 7.520079797958525 | 6.54430980719121 | 7.325568084770691 | 7.685670526310968 | 7.953418065406682 | 7.98130904940899 | 8.441711098019029 | 7.193890730613993 | 7.715812939416613 | 8.092547476203713 | 8.10693662351266 | 7.556310636478173 | 7.388764925803646 | 6.077669686363882 | 7.044785842423983 | 7.235425161396606 | 6.61405072328157 | 6.190048676474147 | 7.912940172665487 | 7.030157328505102 |
| C18:0 | 2.9603798220356117 | 2.878267458249358 | 2.471556897163517 | 2.8865173849879957 | 3.1976178202204 | 3.2163475718853065 | 2.789746197821655 | 2.752465682724595 | 2.7189463286006665 | 2.474376816269586 | 2.785903613555739 | 2.790337585686871 | 3.001569835202599 | 2.5410442092137036 | 2.701457400712976 | 2.2066047596014116 | 2.8840870255078377 | 2.7888876992075735 | 2.501991257964499 | 2.8740565901542277 | 2.595757637542197 | 2.602686461108413 | 2.745138065751761 | 2.7523119976053123 | 2.4494568095591065 | 2.493260108533934 | 2.4095065895212664 | 2.462000321111596 | 2.6442910947112805 | 2.4051738994934655 |
| OA | 10.85142100498246 | 11.77016978524816 | 10.308630967519223 | 10.927251127673529 | 9.421604923374419 | 10.17745086784578 | 9.871704057146568 | 9.980470737454718 | 10.430918079603394 | 8.233983560191097 | 7.953304914490119 | 10.532993363596736 | 9.82264186016998 | 8.416226764130997 | 9.229634930982224 | 8.993456017253894 | 8.979931963249133 | 11.058883863457885 | 10.496521148434159 | 8.78998113330923 | 7.675700443949646 | 9.420336033396573 | 11.288178392849222 | 8.112143948436632 | 8.709650740261234 | 8.379841452110812 | 9.140436089621165 | 9.25773353614506 | 8.949931060890481 | 7.025522027827123 |
| LA | 15.461763512560292 | 14.154073069926643 | 18.102972539602604 | 14.505718950702748 | 13.53252858351991 | 17.73506633920093 | 14.099646525123038 | 15.508213584708512 | 15.238193098222746 | 14.054708735385631 | 13.839622944452444 | 14.944343275715237 | 14.608201114004585 | 16.50354475040547 | 16.250068116197752 | 18.90471250776705 | 15.374622386740631 | 13.015352747338612 | 14.298523092070845 | 13.743880170191865 | 16.093922135443087 | 14.294616010309237 | 14.52630370991379 | 13.492569968925975 | 13.568258637292699 | 13.849330536523146 | 13.923447757046267 | 14.964236195458787 | 12.636951731676662 | 12.132013721554035 |
| GLA | 10.946568546926322 | 12.458851651816449 | 8.836998228921612 | 10.29168053790109 | 10.972231115829109 | 14.699822691507746 | 11.595393788739786 | 11.69356866582592 | 11.456102673055018 | 12.221640655292049 | 12.900770453119835 | 10.661833969093674 | 13.500875888369091 | 17.8250600957443 | 12.955501673266282 | 16.00600632978986 | 14.401952700195544 | 11.567480589524207 | 12.25279298342131 | 15.21556124429965 | 16.900552327774115 | 14.40538249064596 | 13.786500132642823 | 12.634249615643428 | 12.363413251203063 | 11.282518955004555 | 12.490426836359761 | 13.28830859975168 | 14.702046578118292 | 13.703394295756116 |
| ALA | 17.39447112461731 | 18.144802255716957 | 22.410094413766902 | 19.984027608581666 | 17.326995897229757 | 13.96436412171219 | 18.404279123050564 | 17.213922484130528 | 19.806873247938466 | 18.10703689380586 | 16.36022631285553 | 19.939701696186635 | 15.136502886380311 | 13.070539661163407 | 16.249341365644025 | 13.785796170217102 | 14.298476604689577 | 18.615529305521914 | 17.836851222728068 | 14.213637567165001 | 13.128446816608228 | 15.507873068345397 | 15.561196397499957 | 18.622082874625253 | 18.060515916746276 | 20.42777283814606 | 17.724743302916785 | 16.953885214574907 | 15.161838254575011 | 17.789465956038054 |
| SDA | 14.830513775862743 | 14.16 | 14.351222718089863 | 14.600893667019674 | 14.760949634820381 | 15.034250949789003 | 15.286725101403157 | 15.331760264016493 | 16.174274423192575 | 16.198527909468144 | 16.727653835297303 | 16.860019225834602 | 16.978266472597326 | 17.02 | 17.11152711867652 | 17.263957430501772 | 17.325647625918727 | 17.325790980348977 | 17.41613413133258 | 17.431923075517275 | 17.728558148885817 | 18.349048589690227 | 18.40238251155231 | 18.482214741241794 | 18.78181779545112 | 18.832674874305585 | 19.167318955986854 | 19.19150218277715 | 19.348539848965153 | 22.273069251952908 |
| C20:1 | 9.562311846375778 | 9.412242793754826 | 8.415255153100913 | 10.21739606821361 | 9.05788887767007 | 7.819068636607031 | 8.476070529922016 | 9.418444503431964 | 9.085536779635236 | 8.155525404899423 | 8.440513938004507 | 9.991976294012902 | 8.74736303821305 | 8.673557288134562 | 8.586040459223641 | 5.45237764014403 | 7.47863836068111 | 9.704568902135605 | 8.816229623598765 | 8.114695036592488 | 8.475294561513142 | 7.846594759566428 | 8.192599461842137 | 9.356165475590956 | 8.633975201138702 | 8.774025901721691 | 8.689529112833345 | 8.211210502380338 | 7.754553436161851 | 8.364248688356257 |
| DGLA | 1.0617143477984279 | 0.11914253405624368 | 0.06534856581395199 | 0.0 | 1.8773445615343065 | 0.32007235452905525 | 1.0780468653557687 | 0.9988510162705846 | 0.1054613270973865 | 1.564508733788889 | 1.8567351438886972 | 0.5262584738091232 | 0.22913623227290936 | 0.15205153906628177 | 0.13425061297932175 | 0.0 | 1.002009227819847 | 0.33829561358234184 | 0.6046558263874676 | 0.1779320165981049 | 0.12883434784794578 | 0.1350117009014734 | 0.5182635055296682 | 0.13596574574846546 | 0.5459574789813347 | 0.48822934364824644 | 0.136086299795232 | 0.1394609670322321 | 0.15492788095510354 | 0.14099224625455345 |
| ARA | 0.5578756892168405 | 0.061565626324408276 | 0.11491002417006653 | 0.0 | 0.6237648297676585 | 0.08581559469263957 | 0.16641730338578295 | 0.15499356828419614 | 0.07512991281875576 | 0.4159897894312981 | 0.2330693269641168 | 0.1138107648261045 | 0.099799322045354 | 0.060556299989871415 | 0.15443018507958375 | 0.0 | 0.181568502351171 | 0.0 | 0.16392287832442987 | 0.1630239350602749 | 0.0 | 0.11139923422730998 | 0.12991817373556747 | 0.0 | 0.22586160138899383 | 0.0 | 0.11062238092571937 | 0.10458366550072147 | 0.0737048688189661 | 0.0 |
| ETA | 1.3516086039918782 | 0.2348965435146654 | 0.23776774782235605 | 0.26429469558471347 | 2.2778202285158806 | 0.45672251503850003 | 1.4155047077802398 | 1.290808474228419 | 0.28036921152309613 | 2.08998651900026 | 2.1983161354666287 | 0.7737929275350804 | 0.39231934609855745 | 0.21377021755377099 | 0.28260298291438335 | 0.0 | 1.1809706604062362 | 0.3984093686020997 | 0.6529509767665519 | 0.31658775919099286 | 0.20607334176652617 | 0.24980958604268497 | 0.4763104453872975 | 0.2899645856405284 | 0.8229489622882179 | 0.5677841032070139 | 0.2965938105790184 | 0.26748261484199515 | 0.26578338541409935 | 0.3439720762867425 |
| EPA | 0.13338393931540576 | 1.2846008135772446 | 0.0 | 1.602344147605676 | 1.4495192182048269 | 1.008470529144164 | 1.175478029857119 | 1.3805946441014985 | 0.1283517348326641 | 0.36017854503134067 | 1.3670787047239483 | 1.4027626115947713 | 1.7458704615395302 | 1.6021122238313685 | 0.16404926756429294 | 0.7269384075921419 | 0.09679891088014914 | 1.3566483101334692 | 1.4799558342108539 | 1.4946775862870483 | 1.3412068314940162 | 0.0 | 1.305247880406961 | 1.6416696804310824 | 1.4433535177794616 | 0.0 | 1.5796806830195225 | 1.4122412603466057 | 0.0 | 1.793897682239552 |
| DPA | 0.35623984751665577 | 0.30020387923302216 | 0.35363314550488967 | 0.3247257527107413 | 0.4685548618951348 | 0.30185643380096927 | 0.3454033182346006 | 0.3192041356481983 | 0.3534228175742854 | 0.36314519760652625 | 0.34194372728188843 | 0.24657873557050983 | 0.3921961638431377 | 0.25823248874545784 | 0.051802634299532524 | 0.23055331021268669 | 0.3821130462900846 | 0.3586833955408606 | 0.36057684885383046 | 0.3934666315120764 | 0.26961388843853284 | 0.3212224960413154 | 0.23700098582019913 | 0.33428210067839914 | 0.36048510727950295 | 0.31632623178503333 | 0.42771680308195753 | 0.321248740490785 | 0.34106620346138283 | 0.5675678043257325 |
| DHA | 0.0 | 0.0 | 0.0 | 0.0 | 0.0 | 0.0 | 0.0 | 0.0 | 0.07599776124600349 | 0.0 | 0.0 | 0.0 | 0.0 | 0.0 | 0.06891434242162568 | 0.0 | 0.0 | 0.0 | 0.0 | 0.0 | 0.0 | 0.0 | 0.0 | 0.0 | 0.0 | 0.0 | 0.0 | 0.0 | 0.0 | 0.0 |
| Others | 5.201788324316848 | 6.850752253781246 | 4.223612606120071 | 7.170765668378037 | 6.248049442485623 | 7.236189461375054 | 7.318880847185172 | 6.596965768415373 | 6.9982695642011805 | 7.5283033323248825 | 6.773720491859706 | 4.312663869044954 | 7.27882364320504 | 5.344188347181864 | 5.858656150057918 | 7.141367049122855 | 5.540572836234613 | 5.807270162732798 | 4.796697917261793 | 8.052726884603642 | 6.853315382088425 | 7.1389351413257085 | 4.729387330979645 | 7.590580726399045 | 6.229344560589772 | 5.260337399694565 | 6.510671056357966 | 6.394942864885133 | 7.886120974422177 | 5.856509545641202 |
### Chart: DHA2015.3
| Category | 302 | 308 | 325 | 303 | 327 | 317 | 306 | 322 | 315 | 326 | 323 | 318 | 304 | 312 | 316 | 301 | 328 | 324 | 319 | 329 | 330 | 313 | 321 | 310 | 320 | 311 | 307 | 309 | 314 | 305 |
|---|---|---|---|---|---|---|---|---|---|---|---|---|---|---|---|---|---|---|---|---|---|---|---|---|---|---|---|---|---|---|
| C16:0 | 7.7122628023778885 | 7.552394774622082 | 7.2678305227590885 | 7.4414998550706555 | 8.18541963534494 | 6.914502439744125 | 6.940246483679788 | 7.664966933580045 | 7.469185859742859 | 6.616900135862079 | 7.30027151360845 | 8.423931264941855 | 7.281812368300255 | 8.200444143792684 | 7.505797360704529 | 6.146177081223546 | 7.948502802696155 | 7.333792637785179 | 7.076591333890456 | 7.75954085074593 | 7.561794436876896 | 8.101456832147793 | 8.859808947933308 | 7.0911558982418486 | 8.417594060755944 | 8.401373450540822 | 9.151130639931395 | 7.486446431502817 | 8.58958280613496 | 7.984271196332047 |
| C18:0 | 3.562648680955568 | 3.5381204074191723 | 3.756651100875261 | 3.202327629379428 | 3.3766107981299465 | 3.575464665231356 | 3.5565775690594807 | 3.0463726209924062 | 3.226960885175168 | 2.9794840389769925 | 3.248683454213545 | 3.154696956789731 | 3.903911477954144 | 3.564263684965805 | 3.294462373256291 | 3.2273294338370326 | 3.3830943031564775 | 3.6059108492640544 | 2.89723224707594 | 3.38977607816764 | 3.0559674799296848 | 3.1722812302928487 | 4.153683412618198 | 3.8802296952347852 | 3.8055641052314733 | 3.546909838349324 | 3.5697105515292273 | 3.114435077534171 | 4.186659082345363 | 3.9110373569442016 |
| OA | 11.764390266832663 | 13.959087442741184 | 14.56315655260161 | 12.371690065892166 | 16.245840317749817 | 12.168555867818093 | 13.363437574217254 | 15.585179868260331 | 11.715842883944102 | 12.611670583354856 | 13.3982708079695 | 14.573019902267282 | 12.97025511099052 | 16.381619619032804 | 11.93945277566758 | 11.50463135218973 | 14.947232520405775 | 12.09852131787275 | 10.587813776091613 | 12.700582552351095 | 10.86094228844564 | 13.000493991061285 | 18.03411342492687 | 12.022441768942484 | 15.03529932953926 | 9.663897303511636 | 14.507946068946097 | 12.718296917143332 | 14.883685360676004 | 14.099158636594046 |
| LA | 17.699778830619707 | 15.182746932227243 | 14.996858582721908 | 15.623915041887097 | 17.525813973116104 | 15.694150447021348 | 16.09870542209251 | 17.655073112979764 | 15.204444407567582 | 14.472663782230388 | 16.92596521291128 | 18.190505804109023 | 15.801649685865542 | 16.751717701429637 | 18.187817413817445 | 14.101467832203173 | 16.511496644494258 | 14.173012698770084 | 16.72419011646832 | 17.36281185535001 | 15.23699208588136 | 15.724529234459592 | 16.136688596932075 | 15.126700498467537 | 14.8298534726571 | 17.846476044083275 | 14.497261823622802 | 15.020319791030094 | 15.124113391637916 | 14.18944403441274 |
| GLA | 2.723037026609848 | 1.542736851095409 | 1.4225403522124864 | 1.4292296178090984 | 1.3332010311502482 | 1.6417250933448486 | 1.3163583375093804 | 1.086010534555525 | 1.477036051103335 | 1.309727744089764 | 1.362352729585389 | 1.2356042370202152 | 2.8456487137612827 | 2.8171597296193274 | 1.201984929820773 | 1.3319215715889745 | 2.628539035231489 | 1.401675871246748 | 2.0844192651678544 | 1.3472388558924562 | 1.3406933229270899 | 1.2426045054295183 | 2.443319764228224 | 2.731668922320577 | 1.7817465153275045 | 1.293784105887622 | 2.169225525995078 | 1.2344120859973031 | 1.737083214500119 | 1.7160480607208832 |
| ALA | 14.366144686549497 | 19.90266057054408 | 17.061210940219993 | 20.835512595477404 | 17.06955673899839 | 18.766351783934386 | 18.876983706183335 | 17.998077214878386 | 20.862360827234543 | 20.423516109480154 | 17.481702636257957 | 16.227414646021305 | 12.381477782616912 | 14.567484195685442 | 17.688072093254107 | 19.67518964910631 | 13.186125950407197 | 18.69340505116728 | 17.230709544079605 | 15.729034296806258 | 18.874580799277002 | 17.221350470286914 | 8.952143443837663 | 13.364765449991669 | 11.686117629188235 | 14.388401505666009 | 13.679233929732343 | 15.395545652081294 | 9.793378336120464 | 12.669558139675932 |
| SDA | 2.1328305829657315 | 1.4491008561540757 | 1.1478224555416936 | 1.433693906001701 | 1.0884171898785115 | 1.703473812474172 | 1.3384644614082557 | 1.0653545628484757 | 1.3266133769524193 | 1.290590474676111 | 1.2914119811923526 | 1.0454486223242438 | 2.2732627405723482 | 2.175254630314137 | 1.1317294204605957 | 1.4125153861248532 | 1.2153335599138726 | 1.4623766772897595 | 1.9680055601197746 | 1.1921304421221177 | 1.5035514038159528 | 1.16669789705022 | 1.4998097306115759 | 2.0483726324290945 | 1.5796334131896392 | 1.0120314725581887 | 1.7493629752308013 | 1.263263720189881 | 1.4115117571094513 | 1.6363351499366237 |
| C20:1 | 5.726994161739577 | 5.679134509413763 | 6.899271374638562 | 6.124390413212927 | 5.89003820409215 | 6.256413352514078 | 6.535240587884884 | 6.681482742894418 | 6.08434540065682 | 6.893757724980209 | 6.679759739944161 | 5.171189240734525 | 5.6237328634915205 | 6.300773524493492 | 6.343853020581157 | 6.813124991364057 | 5.3585951689047215 | 5.879781397321007 | 5.304660830317053 | 5.528787611463911 | 5.611149002061821 | 5.658727266648239 | 5.995261410469633 | 5.979312277214071 | 5.256219061946811 | 6.790490060267264 | 5.196850787111572 | 6.501568462004517 | 5.844716832119588 | 6.284305011320696 |
| DGLA | 3.1452478242048034 | 0.9286374014378197 | 1.095751704819493 | 0.8744095884949218 | 0.765180507180873 | 1.2419992764820758 | 0.8752195140750413 | 0.718280423163905 | 0.7944040264722316 | 0.93874780776695 | 1.309704473899879 | 0.8261342923283898 | 3.3381780665729592 | 0.8233085460477281 | 1.0552230057878285 | 1.048622521660662 | 2.391053687357855 | 0.8681015569557533 | 0.8686415965821072 | 0.9481236716038742 | 0.9453629719742979 | 0.828054762923496 | 1.2569956807661535 | 1.8928220398356914 | 1.7758204582218888 | 1.3231177837379409 | 0.9386809055452153 | 0.9190225188342654 | 1.8951937872895444 | 1.3974183063144612 |
| ARA | 5.037663486248285 | 5.236830556173524 | 5.786735242048022 | 5.197544948985122 | 6.103005897835923 | 5.879299188179825 | 5.695777576713764 | 5.342190759231362 | 5.0664007163266955 | 4.890078465761075 | 6.108295778688791 | 6.426726976015239 | 5.716817299399535 | 4.4603059352557946 | 6.2131685083390416 | 5.95966101204968 | 5.184006410943632 | 5.837510010299378 | 7.424250918134384 | 7.327014460733497 | 6.216123356897439 | 6.64532851971073 | 5.695801663390759 | 5.56239837437083 | 6.123017476934516 | 6.858247173400341 | 5.130434382911178 | 5.0478444022943165 | 5.868249089474674 | 5.456150763284333 |
| ETA | 3.945502903267899 | 1.8163414240994917 | 1.8597059912238967 | 1.8316052639429863 | 1.2477170956879662 | 2.3173334690739926 | 1.6829233078243644 | 1.2804035474700437 | 1.7200411487195622 | 2.0434012878069066 | 2.1887228189577983 | 1.2768811508562825 | 3.812234283429024 | 1.2753821616273981 | 1.6665274255542684 | 2.157032260072673 | 2.857303352453805 | 1.8887082071920778 | 1.4554635900423547 | 1.4636740632786476 | 1.9134912349827722 | 1.483483410727249 | 1.087733787199416 | 2.6473350286125954 | 1.9276172287689768 | 1.6479830954669217 | 1.1383125676161066 | 1.831957431787211 | 2.033543873543773 | 1.2957996876301334 |
| EPA | 9.684400880427685 | 12.141034039102495 | 11.075130516332486 | 12.480588719379496 | 12.17682912706919 | 11.650593690676695 | 12.372521275840134 | 12.281094517024588 | 13.444671709657458 | 13.62151333164993 | 11.528195661973353 | 12.21542828349796 | 10.474406253126567 | 9.824809303886884 | 11.332681785715783 | 14.317478354960354 | 10.353984487034072 | 14.460800485215959 | 15.165877579976277 | 13.75084085757515 | 15.046720254684562 | 13.762428500776576 | 11.5757969313586 | 12.871422521178124 | 13.005594892475868 | 13.759702382985273 | 14.245353019649171 | 14.754799065754629 | 12.527292227304518 | 13.373191549483776 |
| DPA | 0.9788009809547232 | 1.3149357508696278 | 1.6679486763197302 | 1.4446507150525802 | 1.743577747523972 | 1.6594091945302178 | 1.7249915582499318 | 1.827022624600435 | 1.7571562117302058 | 1.6129042994985463 | 1.5846046910510418 | 1.5744590522105821 | 1.062952880987308 | 1.6576611356256787 | 1.7442037169898614 | 1.927306608485142 | 1.0605117454713442 | 1.733683034018824 | 1.509276494711774 | 1.7080508726008834 | 1.7606342848148813 | 1.6969660080465752 | 1.4532565503708397 | 1.2325979703802912 | 1.4530570012828692 | 1.502233113381725 | 1.7241393205398248 | 2.2438588096319374 | 1.7090294596079876 | 1.9876707038204429 |
| DHA | 1.6440794605266853 | 1.5429894806177742 | 1.6698089170805903 | 1.6991808080311264 | 1.7880210733942101 | 1.8289719515407774 | 1.835723191711839 | 1.8733328403677978 | 1.8939655374721618 | 1.911015010139634 | 1.9524727388379544 | 1.9997591996112645 | 1.9997792094869085 | 2.009762784015407 | 2.1440436035672703 | 2.18509740163013 | 2.2078880667241343 | 2.2888251461998306 | 2.3279660424965525 | 2.373153962069092 | 2.3941075750412932 | 2.4647481790013264 | 2.857105314945766 | 2.9202746823859833 | 2.985888142601447 | 3.30431069894644 | 3.4283427287441364 | 3.5907097717142453 | 3.7014581319126014 | 4.056075352066344 |
| Others | 9.210290861493053 | 7.467984098255148 | 8.495933106374855 | 7.023700264033352 | 4.9648147168672265 | 7.128597738308476 | 7.148585813377162 | 5.210517436308442 | 7.130993556913016 | 7.384674130277662 | 7.097859134585785 | 6.593850923232646 | 9.754471207221785 | 8.13587277125109 | 7.000982585777429 | 7.622758356734417 | 9.092190829680622 | 7.378449158939213 | 6.884162896799472 | 6.880474116475636 | 6.816579829609685 | 6.842658032762462 | 8.358880504297167 | 9.42769429968098 | 9.746151007829877 | 6.928241290901394 | 7.6826922426047926 | 8.267805091084005 | 9.915374153275486 | 7.983797571063626 |I have re-ploted the DHA2015.4 data. Previously you rank-ordered by EPA content. Now it is based on DHA.
After the DHA rank-order, it seems the ARA does not have a clear trend. Should we delete the arrow?
### Chart: DHA2015.4
| Category | 409 | 423 | 401 | 412 | 414 | 427 | 428 | 402 | 417 | 406 | 415 | 430 | 425 | 404 | 405 |
|---|---|---|---|---|---|---|---|---|---|---|---|---|---|---|---|
| C16:0 | 7.2777809024212 | 8.744374879100851 | 7.086331876061818 | 8.42527443460033 | 7.000289777545785 | 7.718934403724615 | 8.337374083046175 | 8.901587949561165 | 7.324611244409632 | 8.332322275704536 | 7.576141217120907 | 8.165089913174926 | 7.754071922124766 | 8.272253833176897 | 8.442999237239746 |
| C18:0 | 3.640985887719034 | 3.144334859042259 | 3.3332763075542857 | 3.1303185242413183 | 3.070333696982288 | 3.3499786258689253 | 3.740015047308968 | 4.55293528114565 | 3.0627789741184976 | 3.797279986269522 | 3.160322889601529 | 3.549201763864186 | 3.198270382628072 | 3.639644973899565 | 4.187585073335166 |
| OA | 9.06638688999849 | 11.174558611335383 | 10.483968901691233 | 10.155796741403265 | 12.226196832633661 | 11.643775204951682 | 11.791082052930973 | 15.709241297242782 | 9.705180211727939 | 10.304009621931211 | 11.294971656775598 | 11.98885828635324 | 9.909522710989533 | 12.232634269307352 | 9.414185497559687 |
| LA | 19.67746210548041 | 20.69611961551624 | 16.916576411116086 | 18.011859732789354 | 17.87263255235234 | 19.341984944429452 | 17.9392707140433 | 17.065553920582346 | 18.498191891653317 | 17.55632976024589 | 17.76513081053887 | 15.137183651727689 | 18.474772401138065 | 17.253389767156357 | 18.144547452801795 |
| GLA | 2.8936583337003774 | 2.8281962061773807 | 2.7197357894948304 | 2.685646291203473 | 3.1703890878596748 | 2.636179691049993 | 2.981167171671581 | 2.549859868985336 | 2.8454935950663285 | 2.8149987097141196 | 2.427539318416487 | 2.593783092495753 | 2.7198196340182648 | 2.8555768459576556 | 2.990162153032596 |
| ALA | 13.153155756839015 | 9.868457727232135 | 11.718245020693448 | 12.258945945909918 | 8.399185731094407 | 8.410631631118806 | 7.194080929808555 | 6.495223843923945 | 9.111414465982405 | 11.110673116876104 | 9.662046653881372 | 10.945191881493734 | 9.67223953531826 | 7.535943963260415 | 7.74830643621665 |
| SDA | 2.8878460362089635 | 2.433211762405205 | 2.580268207231434 | 2.691741759878722 | 2.618413562819335 | 1.8310475253755463 | 2.1902930805156835 | 1.8077027120168079 | 2.3957049670327746 | 2.7124838712969153 | 2.2529957280283153 | 2.965858620650558 | 2.1657497856526513 | 2.281417847828365 | 2.29288551033788 |
| C20:1 | 4.448279980456007 | 4.894780731735148 | 5.610471557819956 | 5.137431744140038 | 5.587705161087021 | 5.942999277406378 | 4.7568109510449395 | 4.433004776129308 | 4.870329387994781 | 5.586959227588428 | 5.285080005761547 | 4.752557738052198 | 5.083247248352173 | 4.439979990486972 | 4.960996978488573 |
| DGLA | 2.525952370292888 | 2.780466048009986 | 2.7423858878927287 | 3.028372501844288 | 2.526946974021288 | 3.0249849573771526 | 2.5078509720454667 | 1.5980213148551607 | 3.1244731210480774 | 3.1486351725238437 | 2.5340914038568694 | 2.4349104792092287 | 2.506753133814424 | 1.960357165779792 | 3.0758382658102077 |
| ARA | 5.70267196940635 | 7.813248172157668 | 6.932892393576806 | 5.783827756083506 | 10.14862215802188 | 7.7480938723157395 | 9.701186774888917 | 7.486097175148899 | 7.960954543706707 | 7.029865622566338 | 8.754846630592274 | 6.924490893174503 | 8.248239216772332 | 9.179272764897611 | 8.790510785449305 |
| ETA | 3.1414262943011537 | 2.617660132655446 | 3.271753270577217 | 3.797404481366586 | 2.2993162933798197 | 2.671394202930023 | 2.095267975092189 | 1.5546558477042685 | 3.122393645385233 | 3.4065389298976614 | 2.655814370463403 | 3.3368655354124477 | 2.550483319998731 | 1.9025225768328917 | 2.9297955505111153 |
| EPA | 14.20016834863842 | 13.129884718164433 | 14.502840224969825 | 13.329139812271494 | 13.452658316246563 | 13.57015483722812 | 14.282394521550398 | 14.718585297124392 | 15.820579635386983 | 12.235023792404764 | 13.95242365539989 | 14.397589121055752 | 15.249113665768613 | 15.797773044214923 | 15.26776497493245 |
| DPA | 0.8012064005108122 | 0.7392536847640431 | 1.0574414898176445 | 1.0236130558567038 | 1.0809808441778304 | 0.9992113189065112 | 0.8352467222306869 | 0.8136960035563696 | 0.9809238061786167 | 1.0540849457023471 | 1.3000688791765636 | 1.2592935696026424 | 1.0354803742394727 | 0.9943743656183865 | 0.8246276803240374 |
| DHA | 1.680878778623233 | 1.929917769026072 | 1.9312542673670816 | 2.0749589643399444 | 2.014429755027344 | 2.1798345814477744 | 2.275543129736196 | 2.279028625826786 | 2.3589095492603005 | 2.3636787942347164 | 2.393142793403564 | 2.4434055440150075 | 2.4502011323201365 | 2.4821963039115937 | 2.914037280256813 |
| Others | 8.36760788190035 | 6.0696181439819945 | 8.476963963721277 | 7.8344852545383645 | 7.7780254000883335 | 8.286585887578397 | 8.28137830062403 | 8.891829228419377 | 8.166300189217953 | 8.127336716483697 | 7.685515529323969 | 7.880979793588706 | 8.277438626659789 | 8.124255952797231 | 7.231159199914201 |
### Chart: DHA2015.5
| Category | D5.17 | D5.16 | D5.11 | D5.25 | D5.28 | D5.26 | D5.19 | D5.18 | D5.22 | D5.21 | D5.06 | D5.27 | D5.24 | D5.10 | D5.30 | D5.09 | D5.05 | D5.15 | D5.03 | D5.12 | D5.14 | D5.29 | D5.13 | D5.08 | D5.04 | D5.20 | D5.01 | D5.23 | D5.02 | D5.07 |
|---|---|---|---|---|---|---|---|---|---|---|---|---|---|---|---|---|---|---|---|---|---|---|---|---|---|---|---|---|---|---|
| C16:0 | 8.297091623967015 | 7.921168987583989 | 8.868108881564627 | 7.0813823862 | 8.900748060870795 | 7.936492267331981 | 9.560840877096656 | 9.024858369355826 | 8.541920481396273 | 8.831076717017558 | 8.78686970071465 | 6.979158483314554 | 8.94765704421744 | 9.24808821649967 | 8.127777119648774 | 8.33673739710012 | 8.69666168884341 | 9.108958745521129 | 7.516164700231267 | 8.88656724280508 | 7.523823890309156 | 8.357609426068514 | 7.86961430250324 | 9.412929910293407 | 7.9985313338807495 | 8.82653389790505 | 8.543394298137683 | 7.410837247004371 | 7.561919931410773 | 9.547279700254863 |
| C18:0 | 6.262556419656549 | 3.9242695776546728 | 5.129229721125868 | 5.9278027208848245 | 4.424106536554847 | 4.110791241828028 | 3.521463219585203 | 4.574615749444632 | 3.9690797762664194 | 4.280933254532962 | 4.116416228971554 | 3.9845520188985373 | 3.6765300816437 | 4.094499629227045 | 4.2939082438419325 | 3.701102855305425 | 4.237725207772167 | 4.094214874872096 | 4.062491604206874 | 3.886463359282196 | 3.467754696034418 | 3.849584882777361 | 3.436758746557311 | 3.8965025245494935 | 3.4125670525902922 | 3.544316166314814 | 3.407261034777122 | 3.537626588621618 | 3.53692587098645 | 4.391610699764407 |
| OA | 5.8810049903600365 | 5.066364411041425 | 4.898215704846505 | 5.475712693914263 | 4.94971425746993 | 5.638656664420473 | 4.947376856123313 | 5.086066685119021 | 4.186468952276816 | 5.363628317000379 | 4.81033907913685 | 5.828220478510627 | 4.6958192417943225 | 4.546299525551959 | 4.884530559849927 | 5.067548021526896 | 5.222727161379843 | 4.848342838428986 | 4.5329286199680645 | 5.320374504126953 | 4.079177980348741 | 4.8425102589616404 | 4.51321308345789 | 4.196745967497042 | 4.184183751168611 | 3.7537228416354935 | 4.404730530757855 | 4.636408777390315 | 4.5136475704989 | 5.623162452923974 |
| LA | 22.74476063456606 | 23.974538932804954 | 21.88346319361111 | 21.02143049730889 | 23.84415825513117 | 22.12120366921196 | 22.707878742187187 | 20.352175979866672 | 20.626614619905634 | 22.93430772348434 | 18.909450042128064 | 23.21930038642511 | 19.28274828672053 | 20.011485742765522 | 21.397175128907932 | 22.01770445373882 | 21.04576519361047 | 22.069312064856263 | 22.362365883151497 | 24.166313756881173 | 20.6034047633474 | 20.345603755261212 | 18.646115727199547 | 17.00482710152517 | 16.902687951519802 | 18.487789725549817 | 17.232503645540156 | 17.033906241668003 | 18.139761633139234 | 21.910981128116738 |
| GLA | 1.6795787857978801 | 1.8436928173193416 | 2.241146816877258 | 1.7563492773513871 | 2.4554103202352207 | 1.8967501939416713 | 1.990722195208033 | 2.0004806056441486 | 2.2289678346523174 | 2.0613466592518135 | 1.9282205626459656 | 1.8270301576520367 | 1.9028931894241163 | 2.084888965238421 | 2.2798045615706957 | 2.086141888006183 | 2.1879172931747823 | 2.2330351097672416 | 2.048417612197585 | 0.12677731738869213 | 2.038924349509123 | 1.841146508290477 | 1.559745722624444 | 1.952584825035552 | 2.3013612685899054 | 2.0854428589097194 | 1.411595385338498 | 1.6176456469049143 | 1.8029124361432778 | 2.2589168277211655 |
| ALA | 18.46010272860465 | 18.548200177777435 | 18.326058747793503 | 18.477983405977955 | 15.652175404331647 | 19.043617828895645 | 17.349363633946258 | 19.025061159835406 | 19.20584008152419 | 15.820079701093873 | 21.1729445053274 | 15.405358227300665 | 19.928034843781386 | 17.277824548312797 | 15.839495693501922 | 17.499514751703135 | 15.78789566815641 | 16.444931267873066 | 16.088924297272097 | 16.230409641536113 | 19.620926615621524 | 17.78029497951051 | 21.309248238447715 | 21.86425580924773 | 20.607754045425207 | 17.443740070998295 | 23.081668457962724 | 22.022992245394434 | 19.92736110166521 | 21.879991872763995 |
| SDA | 1.5263409826013588 | 1.5156939799076765 | 1.8984320044434346 | 1.6187123434821662 | 1.7527240380041529 | 1.9022885666692813 | 1.8622515814142602 | 1.999497638382445 | 2.016635190387252 | 1.7691276523459438 | 1.9873551142655843 | 1.4700855234027084 | 1.9557110223160266 | 2.110387821268518 | 2.1402744354853476 | 2.081163579567153 | 1.8885991135654792 | 2.125150002597464 | 1.8452835122618403 | 1.5590335711494492 | 1.9003722212207699 | 1.7962949696779384 | 1.7292639257189955 | 2.359965196775456 | 2.351580668234851 | 2.2062295431415473 | 1.8493660342412528 | 2.054522590453451 | 1.9023234086860292 | 1.8616984556372638 |
| C20:1 | 4.479066086583074 | 5.480030794959081 | 4.165733550965683 | 4.310553963929268 | 4.217069178307988 | 4.330626118595188 | 3.5811531878919367 | 4.415535992530658 | 3.5928178904548442 | 4.269862095532972 | 4.314589419690862 | 5.258495277590206 | 4.5671981058574325 | 4.331287091575792 | 4.100745147674115 | 4.477367688417537 | 3.8946716726816244 | 3.768625479275496 | 3.8337849935787607 | 4.566259275303136 | 4.88270423759349 | 4.452824168076271 | 5.080076108874608 | 3.8787379371536437 | 3.937044012329308 | 4.154910136133565 | 4.998064199394757 | 5.328408074285384 | 4.964331244408704 | 5.230925770270727 |
| DGLA | 0.41755332517910615 | 0.5783337300566279 | 0.4376768500745749 | 0.41311868786675054 | 0.5142350489202129 | 0.503508040191484 | 0.4176045932195078 | 0.4315602683119611 | 0.47438632915090095 | 0.5032281465924953 | 0.42630159818540914 | 0.5747946830492006 | 0.47218382175697277 | 0.4715364131448409 | 0.48169494880724323 | 0.3832586566322639 | 0.4389685446917762 | 0.4949214664842762 | 0.5061289682650202 | 0.508005821935451 | 0.49787635013775167 | 0.49011553846807115 | 0.4689811142732501 | 0.3471993189653206 | 0.40176076865532995 | 0.48093533391463505 | 0.38055197565830706 | 0.36732491494751207 | 0.43552118009121027 | 0.5474987424438899 |
| ARA | 2.3234673796595064 | 2.4687014169273067 | 2.5779034626218573 | 2.4055288814147273 | 3.549676987291276 | 2.89031865000137 | 3.429074857956378 | 2.4443520735346165 | 2.638046008519345 | 3.0678347042801715 | 2.3267478771234384 | 3.513709410821319 | 2.4756618525922467 | 2.8020279802660597 | 3.3817583476588693 | 3.133703841716605 | 3.1166985131286444 | 3.0369405546847608 | 3.380992701489469 | 3.3833022885026827 | 2.609147026233816 | 2.885411940861775 | 2.4667887959501495 | 2.14901287128061 | 2.456291790990368 | 2.8144760131474498 | 2.256988483555354 | 2.4140406162034704 | 2.7603166773705428 | 3.22943054643979 |
| ETA | 1.787548883416619 | 1.977241018708633 | 1.7547024576969326 | 1.8455599407542416 | 1.5792428826099072 | 1.9484737446391598 | 1.5386921200915489 | 1.9850509968941275 | 1.9404880112800118 | 1.8856586795598171 | 2.0310960408273147 | 1.9357877830891128 | 2.20755444962375 | 2.181805347582551 | 1.8800106107960373 | 1.6378859630364038 | 1.887460060201654 | 1.968492561864167 | 1.8144018185644288 | 1.965011781735847 | 1.991730263659025 | 2.049421796292018 | 2.2726010503276766 | 2.074163409716525 | 2.1307135027331254 | 2.4102438251142053 | 2.157263061824367 | 2.1555365876880384 | 2.1301147741225726 | 2.450999073655168 |
| EPA | 8.249615285271151 | 8.179278222590506 | 9.506489583282935 | 9.829125940989927 | 9.751913768165686 | 9.35592567805577 | 10.2244419389723 | 9.315102763612977 | 10.909568825955848 | 9.268076280852148 | 9.410644589481489 | 9.975066373678235 | 9.893031792044855 | 10.456731597956068 | 10.637935782008617 | 9.820676039698458 | 11.17859564834836 | 9.803826090457017 | 11.04452213085798 | 9.915268090050553 | 10.206481645615396 | 10.972093279579207 | 9.990294083263112 | 11.22161364871513 | 11.857729603424396 | 11.763473247783882 | 9.180382034588394 | 10.25078889381492 | 10.747309057720978 | 0.5514238300091814 |
| DPA | 3.5016873193162477 | 4.084327377489222 | 4.066966748859546 | 4.88760809673262 | 4.060392504455624 | 3.8706934421679278 | 4.128650270997747 | 4.194824173222403 | 4.435361187659179 | 4.288136382774044 | 4.312995101407585 | 5.2123818001886 | 4.444947070157265 | 4.798598284537709 | 4.41530240668673 | 4.518077117212555 | 4.548304723550294 | 3.981490805243504 | 5.138069875776435 | 4.844658591510578 | 5.077985154616125 | 4.908551394880304 | 4.843863491698218 | 4.175137916178923 | 4.85804978695263 | 4.503806835029667 | 4.554395930032397 | 5.376764998485776 | 5.345338743191273 | 4.943421644038676 |
| DHA | 4.914459621266553 | 5.411407471607205 | 5.86442186974239 | 5.969532890856323 | 6.010931473848157 | 6.383273007125363 | 6.505004338973605 | 6.686798280575717 | 6.712077675305386 | 6.79129262546439 | 6.828184261028181 | 6.9302867784270905 | 6.958899117783919 | 7.0102500359991975 | 7.082215064594303 | 7.132271846814218 | 7.167404940452187 | 7.268847220907264 | 7.327982199323258 | 7.484784491355384 | 7.491761764303877 | 7.6663013876147215 | 7.693133018980174 | 7.693867671739113 | 7.880833820334314 | 7.96013793623881 | 8.010351710866075 | 8.50774249135229 | 8.580584456033849 | 8.650660427150065 |
| Others | 8.14210653172445 | 8.633640220731563 | 7.737291764614733 | 8.498644747462201 | 7.894521787471233 | 6.533883569878919 | 7.433230768671458 | 6.940756812651299 | 7.842603687410887 | 7.698316236949232 | 7.509179234636502 | 7.06520260483534 | 7.103552676658066 | 7.867922701076123 | 7.735187994374296 | 7.407197420490299 | 7.710113441103722 | 7.344691592400368 | 7.968463090732336 | 5.617129799535507 | 7.524230437387004 | 6.776758469682859 | 6.641567232445574 | 6.681349854737618 | 7.227534187880565 | 7.614566614633878 | 7.385383721660774 | 6.618558024440469 | 7.101426097589675 | 6.32105483487126 |
Figure S7 Single seed fatty acid composition analysis from the 2018 field trial DHA2015.1-5 lines.

## Slide 9
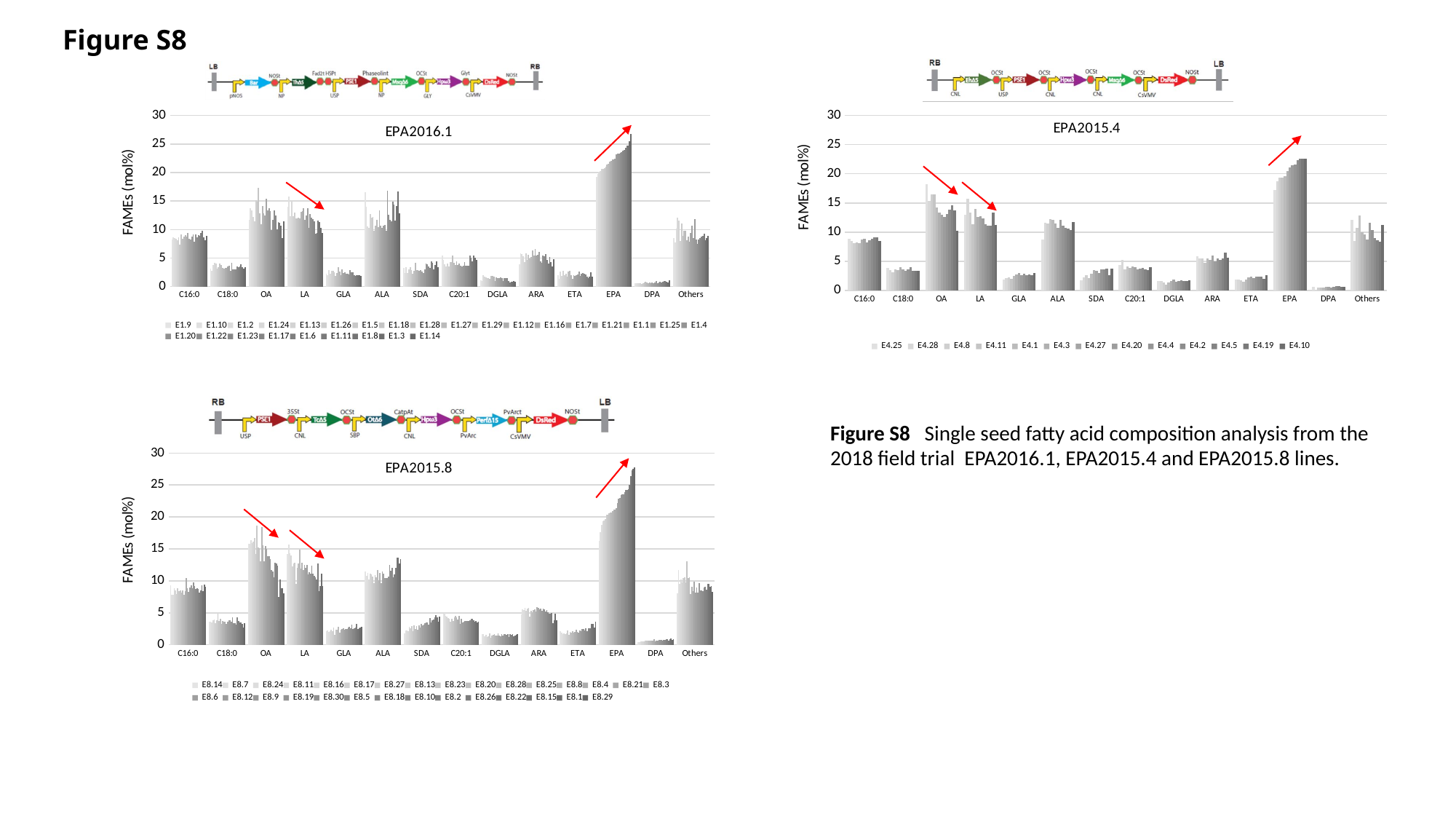

Figure S8
### Chart: EPA2015.4
| Category | E4.25 | E4.28 | E4.8 | E4.11 | E4.1 | E4.3 | E4.27 | E4.20 | E4.4 | E4.2 | E4.5 | E4.19 | E4.10 |
|---|---|---|---|---|---|---|---|---|---|---|---|---|---|
| C16:0 | 8.894651403753196 | 8.425442394229233 | 8.07371422033854 | 8.256407049008418 | 8.111284604091356 | 8.719551482641155 | 8.890391416813507 | 8.188781720301831 | 8.65312834800997 | 8.894268866430583 | 9.073594190132745 | 9.045424516542925 | 8.493461967289381 |
| C18:0 | 3.8833377367991404 | 3.4561842414912207 | 3.1162229049283985 | 3.568419963013647 | 3.4620696684299905 | 3.92516557164883 | 3.6498799538081603 | 3.328276503564587 | 3.6589820498055263 | 4.015053547919879 | 3.339513977867398 | 3.307055151063653 | 3.313296307934906 |
| OA | 18.228703376536927 | 15.282762742301951 | 16.467398384962497 | 16.493710491758133 | 14.204479112162902 | 13.327950685538122 | 12.971796807831655 | 12.652072302596638 | 13.080036180487607 | 13.832115443024069 | 14.65308318805154 | 13.671927356433004 | 10.237427799116634 |
| LA | 12.952653839231363 | 15.749018962381099 | 13.369540577182324 | 11.306401998313563 | 14.02414520061181 | 12.547651741121896 | 12.757721861371405 | 12.364808967980595 | 11.358622046615967 | 11.10427639139142 | 11.126111901047103 | 13.34713282975381 | 11.221835857127461 |
| GLA | 1.8787789625032363 | 2.084280421104056 | 2.2117693786088655 | 2.0292648361598364 | 2.425455378727527 | 2.7099402802492865 | 2.9901078427125882 | 2.6386550855635282 | 2.912453216443992 | 2.6340809768812687 | 2.713128092949439 | 2.543985922583786 | 2.941277821730639 |
| ALA | 8.780622500663892 | 11.56586743793718 | 11.515257343049512 | 12.248871653374897 | 12.146822559329552 | 11.420528134314159 | 10.714633780907374 | 12.09446479243643 | 11.132300861725305 | 10.66368586318084 | 10.551671384002551 | 10.315613836545035 | 11.719539469492654 |
| SDA | 1.7422952514727428 | 2.226121204347815 | 2.6396051626104917 | 2.0872942846809894 | 2.866987960316657 | 3.499558620992505 | 3.359097396011848 | 3.007782209602149 | 3.670554069809703 | 3.6137034670047106 | 3.6747067122265973 | 2.548529836395033 | 3.6794392929482806 |
| C20:1 | 4.301731615657982 | 5.187006883479636 | 3.631792913657282 | 4.11220009894548 | 3.8901220766593805 | 4.044881988436897 | 3.9883715981366152 | 3.6164262349244485 | 3.7047140196629003 | 3.868598161691988 | 3.5921934125914876 | 3.537576186799576 | 4.033003052433445 |
| DGLA | 1.6323938965854956 | 1.5530428661829527 | 1.3361882972193502 | 0.9495242691979644 | 1.3785602906173575 | 1.6067307497949608 | 1.9144638786097898 | 1.4641917648679383 | 1.5514714933034806 | 1.7675578794383686 | 1.5704510165549006 | 1.588022861187077 | 1.73847950454019 |
| ARA | 5.898759453729637 | 5.490271059608095 | 5.465259164007574 | 4.717167666143593 | 5.511461027368103 | 5.232971215834812 | 5.987070105868392 | 5.017748071153997 | 5.462607232352284 | 5.242342051360581 | 5.502862337967726 | 6.483609758170107 | 5.652324625833882 |
| ETA | 1.862575501582052 | 1.810308521167686 | 1.758186362742863 | 1.4586529515907491 | 1.8569392666992524 | 2.2470723528537264 | 2.4003351502124217 | 2.098479389537033 | 2.351125342158767 | 2.362655085483387 | 2.3398323308553426 | 1.9731560373665462 | 2.563904864687817 |
| EPA | 17.260081283538536 | 18.75357212313286 | 19.308814746071384 | 19.333813112451285 | 19.635598397150826 | 20.475233539140117 | 21.062720567569766 | 21.44986024659702 | 21.545913824856957 | 22.337098396636737 | 22.56904645632569 | 22.58595909143928 | 22.589029342071292 |
| DPA | 0.5522491138327746 | 0.0 | 0.4397413274848824 | 0.5423405591715693 | 0.46820549114209764 | 0.6397495751370204 | 0.6308950642853343 | 0.5131509933515446 | 0.6264942189743588 | 0.7374474373865697 | 0.6718847844999445 | 0.6480194148446673 | 0.622708307167981 |
| Others | 12.13116606411302 | 8.416121142636227 | 10.666509217136056 | 12.89593106618987 | 10.017868966693207 | 9.603014062296515 | 8.682514575861125 | 11.565301717522262 | 10.291597095793183 | 8.927116432169612 | 8.621920214927519 | 8.403987200875495 | 11.19427178762546 |
### Chart: EPA2016.1
| Category | E1.9 | E1.10 | E1.2 | E1.24 | E1.13 | E1.26 | E1.5 | E1.18 | E1.28 | E1.27 | E1.29 | E1.12 | E1.16 | E1.7 | E1.21 | E1.1 | E1.25 | E1.4 | E1.20 | E1.22 | E1.23 | E1.17 | E1.6 | E1.11 | E1.8 | E1.3 | E1.14 |
|---|---|---|---|---|---|---|---|---|---|---|---|---|---|---|---|---|---|---|---|---|---|---|---|---|---|---|---|
| C16:0 | 8.288784699552426 | 8.592728424160196 | 8.504458330428122 | 8.3960857212879 | 8.143223989999877 | 8.591675887446002 | 7.308916352899164 | 9.15252776447849 | 8.409748334205398 | 8.802876396320345 | 9.058882467686098 | 8.78310618974807 | 9.376920965657037 | 8.395366713415227 | 8.306231341904397 | 8.723230949805291 | 9.09373751453803 | 7.865128251240243 | 9.185613722679927 | 8.698162586346626 | 9.014057177682062 | 8.843756822860966 | 9.4015560974309 | 9.739903079209345 | 8.587834537102585 | 8.102374442523503 | 8.880567300125682 |
| C18:0 | 3.179061665726629 | 2.7988137578363963 | 3.738317841151208 | 4.201352648738644 | 4.047051052509254 | 3.1660082025500533 | 3.459544351915942 | 4.079775610434726 | 3.8126815041133555 | 3.2574422353153563 | 3.1508935126133677 | 3.260657190231542 | 3.2474428365783115 | 3.5892579920034295 | 3.6456221668941793 | 2.81166224186331 | 4.23170287559648 | 2.98932590541941 | 3.0360108089599853 | 2.994587078481768 | 3.488809252689082 | 3.3972194145084527 | 3.4826488210495046 | 3.9509625476959926 | 3.357722858554806 | 3.1193039723636113 | 3.4576332632536824 |
| OA | 11.693626699697813 | 13.720553811148179 | 13.314939021403385 | 12.263106745881668 | 11.497662335921516 | 15.153599186431428 | 14.845036033560959 | 17.28592931384681 | 12.894047256320462 | 10.910521582284535 | 14.190354135989772 | 12.864625710867573 | 12.492685211608034 | 15.421893708861864 | 13.36691761048767 | 13.78970004361457 | 13.176618505483177 | 9.87835268470871 | 11.7218045789346 | 13.334827306982993 | 12.510071186162396 | 10.072513330350546 | 11.288920651113775 | 11.190737917586357 | 10.740870888099087 | 8.471899985046607 | 11.41644268606801 |
| LA | 13.9429985090952 | 15.82476011251864 | 12.36593202879451 | 14.824223946391266 | 12.337174656991598 | 13.006966916133804 | 11.92173859368852 | 11.94910375686831 | 12.134057307188304 | 11.959285518304224 | 13.0364907791749 | 13.245386955599567 | 13.784151500033994 | 11.717906950972987 | 12.425166615339847 | 13.69843556683901 | 10.319465783816957 | 12.71992061284516 | 12.048739152087085 | 11.841777015378106 | 11.393884221176487 | 9.314973745558193 | 9.416235386460398 | 11.572169699972505 | 11.338544241179717 | 10.319824738385902 | 9.351399354767477 |
| GLA | 2.25975821488914 | 1.939952408793736 | 2.9061047718502135 | 2.2572417622253518 | 2.712804292999103 | 2.7254347426015135 | 2.4590932721737873 | 1.8290328709076045 | 2.345968825643077 | 3.3919883448834507 | 2.5905016390675923 | 2.0556639225495936 | 3.0351347405237874 | 2.3829688373598907 | 2.5488748343705914 | 2.2763850417058022 | 2.230967167226037 | 2.1334966458896196 | 2.8646568603627305 | 2.4871514520564597 | 2.5750712151893946 | 2.007622467195235 | 1.9332417086157432 | 2.0399116158056656 | 1.9792229406634692 | 1.9918738457432077 | 1.8973022735943126 |
| ALA | 16.53904054579354 | 13.96712226933278 | 10.586329007871386 | 10.360818073108248 | 12.698506764963103 | 12.054492583554998 | 12.151476023143507 | 9.754537499180998 | 10.724288601475378 | 11.63507165873736 | 10.396281491862817 | 13.33838019176781 | 10.642500232914642 | 10.310728095131982 | 10.703991780691245 | 10.777375304723678 | 9.770058659999814 | 16.821097077252865 | 12.649760165961203 | 11.691433421290904 | 11.38410397633112 | 14.897129545833955 | 14.441412859231034 | 11.603276807404548 | 14.164360693763186 | 16.697474865802576 | 12.813346781947054 |
| SDA | 3.302385479317667 | 2.3636304110238653 | 3.3857989496992897 | 2.377510590612771 | 3.0559245861157325 | 3.403234437146918 | 2.930771390076247 | 2.3060238621628653 | 2.788315504307658 | 4.140715855939653 | 2.9059371225038606 | 2.8549608760890353 | 2.825797617861491 | 2.9072368490967437 | 2.6824621795774632 | 2.428682128370208 | 2.9672070884536614 | 4.066524438185387 | 3.968984343219444 | 3.4930122862261097 | 3.3242566651491914 | 4.445148211495145 | 4.226965763854666 | 2.9797588290797985 | 3.7817923983260493 | 4.404176182352499 | 3.400512777488974 |
| C20:1 | 5.388212114826225 | 4.647169196291215 | 3.941319247151022 | 3.5740711711105235 | 4.085534275095332 | 3.5339811640898477 | 4.239046750353426 | 4.289950174452046 | 5.404241662892774 | 4.141570422013664 | 3.80477200839744 | 4.4558097929606335 | 3.7427701906362665 | 4.001625207750881 | 3.5965918821752947 | 3.491077885081816 | 3.609311934567014 | 4.300367725027982 | 3.6383124820104507 | 3.6344429544152477 | 3.6761315098144083 | 5.497319602428292 | 5.046645886147807 | 4.387919141889415 | 5.499657899762128 | 5.036519611327425 | 4.665334663639587 |
| DGLA | 1.1041434217860804 | 0.9883351472621049 | 1.99857202601143 | 1.692816602998041 | 1.6611528263858397 | 1.432312672542528 | 1.447430826294348 | 1.3865065479259682 | 1.8344869498779848 | 1.9045318181769948 | 1.6829610002550186 | 0.9167037441820759 | 1.5482783285299833 | 1.4323047601053889 | 1.4503363157700544 | 1.548881132747194 | 1.5425241650643986 | 0.9747737169003654 | 1.4624847179743103 | 1.5424617633938869 | 1.5164826844480441 | 1.0225011700365918 | 0.7879923981449364 | 0.9102569068519758 | 0.8867730705103211 | 1.028945018109544 | 0.8546415248508126 |
| ARA | 3.974808008947776 | 5.851408550471415 | 5.707185620105637 | 5.331459490161295 | 4.286576211879911 | 5.795084982902654 | 4.635668687419734 | 5.5890540994154625 | 4.926543641562013 | 5.14608126005945 | 6.400837051447082 | 5.433403777711806 | 6.539581231120179 | 5.482699539519205 | 5.581226422580222 | 6.036442384710852 | 4.459181639078054 | 4.143712743059385 | 5.419953243987448 | 5.338166488045189 | 5.754892324170741 | 4.703153316892732 | 4.043671079051582 | 5.203858303052584 | 4.297814319044314 | 3.577104018435424 | 4.822438737957993 |
| ETA | 1.9474644686643643 | 1.304923288978249 | 2.619455453421565 | 1.8756728945856587 | 2.712602602840525 | 2.006865272893453 | 2.2864233946118966 | 1.7624474981791936 | 2.62630374161176 | 2.757580044050686 | 2.0555708522837315 | 1.3642072673636259 | 1.9615817296742644 | 1.9219992274621447 | 1.95350152000821 | 2.0936082876252415 | 2.585322824416112 | 1.9355367229079157 | 2.2959389857809427 | 2.4007588905509736 | 2.230247626219845 | 2.1640755356142423 | 1.7349716844336964 | 1.4440715223430198 | 1.7537098323702296 | 2.473266517833913 | 1.6933533959298066 |
| EPA | 19.249135360531124 | 19.70185676407297 | 20.126176531320933 | 20.242248143031585 | 20.625533896803894 | 20.63485603840173 | 20.700794157178883 | 21.062487384081923 | 21.44953455769671 | 21.50744329215965 | 21.908625829811868 | 22.035240118041905 | 22.293128282327615 | 22.2966459474622 | 22.37862121969914 | 23.197365805014556 | 23.287754514462023 | 23.292033792053417 | 23.49125590671525 | 23.53433960338446 | 23.869191597414755 | 24.002732826139514 | 24.345864550721984 | 24.653721694922428 | 24.6766002572592 | 25.52132448916252 | 26.770672638182955 |
| DPA | 0.6060425881206982 | 0.604456659421103 | 0.6235400877230591 | 0.5563307730873205 | 0.5417701789473305 | 0.50514738050356 | 0.5553526243792655 | 0.715642664097614 | 0.8576185566033421 | 0.6907829001801581 | 0.6419501917318009 | 0.6673825824046922 | 0.6264418098956434 | 0.7326097530136927 | 0.6315621506621959 | 0.6696812390119032 | 0.9172565183721085 | 0.6285980348202341 | 0.6994933212213713 | 0.798101074983699 | 0.744632496189667 | 0.824661866868529 | 0.9699917401911332 | 0.9976954466588999 | 0.8606446372534353 | 0.7676875905989807 | 1.0668591861335799 |
| Others | 8.52453822305133 | 7.694289198689144 | 10.18187108306822 | 12.047061436779712 | 11.594482328546974 | 7.990340532801497 | 11.058707542304326 | 8.836980953967975 | 9.792163556501773 | 9.75410867157447 | 8.175941917174677 | 8.724471680482083 | 7.883585322638739 | 9.40675641784439 | 10.728893959839493 | 8.457471988886594 | 11.808890808926158 | 8.25113164968931 | 7.51699171010528 | 8.210778078463578 | 8.518168067362817 | 8.807192144217629 | 8.879881373552857 | 9.325756487527492 | 8.074451426111473 | 8.488224722314264 | 8.909495416060071 |
Figure S8 Single seed fatty acid composition analysis from the 2018 field trial EPA2016.1, EPA2015.4 and EPA2015.8 lines.
### Chart: EPA2015.8
| Category | E8.14 | E8.7 | E8.24 | E8.11 | E8.16 | E8.17 | E8.27 | E8.13 | E8.23 | E8.20 | E8.28 | E8.25 | E8.8 | E8.4 | E8.21 | E8.3 | E8.6 | E8.12 | E8.9 | E8.19 | E8.30 | E8.5 | E8.18 | E8.10 | E8.2 | E8.26 | E8.22 | E8.15 | E8.1 | E8.29 |
|---|---|---|---|---|---|---|---|---|---|---|---|---|---|---|---|---|---|---|---|---|---|---|---|---|---|---|---|---|---|---|
| C16:0 | 9.31939609538222 | 7.8745981071112015 | 7.826132759169259 | 8.898384374224158 | 8.51987605691535 | 7.932892336985868 | 8.809494145975936 | 8.413284898467216 | 8.500207972792422 | 8.20323657623557 | 8.446732548014309 | 7.830778636540384 | 8.433755315748932 | 10.481286771358741 | 8.8648099995196 | 8.30227394114613 | 8.974547662300754 | 9.256553761874825 | 8.834254016688051 | 9.704851271150883 | 9.149095837515764 | 8.781698733093233 | 8.856636244906879 | 8.70704695403187 | 8.176342639735793 | 8.512579880893671 | 9.332659025296529 | 8.356401742354514 | 9.448555187320636 | 9.105114864210922 |
| C18:0 | 3.6060866947295227 | 3.6588067470831973 | 3.4818513810007183 | 3.7804020650738166 | 3.9999127559903918 | 3.3316176546792224 | 3.8828186595913423 | 4.872265063587419 | 3.6706955157325383 | 4.009959735519847 | 3.2459326020490114 | 3.7766052608480236 | 3.6116190117416083 | 3.551628059417466 | 3.209511995645158 | 3.5721622502631765 | 3.8265622706695943 | 3.7925839714965663 | 3.633929860187166 | 4.24681162870019 | 3.3641992597470165 | 3.359228551203435 | 3.1557059042101443 | 4.250399363649471 | 3.761706803570665 | 3.4837008714667164 | 3.468423709089289 | 3.225798708070876 | 2.6815657266286177 | 3.337546023853526 |
| OA | 15.808191795582283 | 15.810881171913373 | 16.331618912044867 | 15.876881843230045 | 16.173331195750766 | 16.682900542128117 | 14.151671142796317 | 18.627410933769273 | 15.230418024308067 | 15.076177061061939 | 13.082727391722077 | 18.451379953604892 | 15.608819552994598 | 13.049253937251981 | 15.498399734055441 | 14.958410116812743 | 13.827897221269769 | 13.873662091010136 | 13.42571913440264 | 11.708197454371735 | 11.516119930699626 | 10.57463431689979 | 12.876061092605193 | 12.693965777867744 | 12.37230542395375 | 7.518348345877325 | 10.170389854537863 | 8.826267449980628 | 8.79118851150139 | 8.018337305516265 |
| LA | 14.19358632806549 | 15.668391891224454 | 14.32452177002962 | 14.018850910196742 | 12.286714449004592 | 12.676441506961403 | 12.879451398581775 | 9.52748776305828 | 12.04319309762319 | 12.69682204038235 | 14.870912988374746 | 11.878141275054578 | 12.861493867553085 | 11.64498980088206 | 12.483613136745912 | 12.05648953447259 | 12.445626058107075 | 11.003846710358506 | 11.350636790214176 | 11.131004675753823 | 12.371726569553735 | 11.170641446348954 | 10.736155003823331 | 10.576600705025694 | 10.244211547891974 | 12.745350220773444 | 8.441830831133906 | 9.228568499319051 | 11.1641071878562 | 9.189420946114009 |
| GLA | 2.1159815956638406 | 2.2082384946294624 | 2.029944972859912 | 2.074855001385618 | 2.355741622961615 | 2.1190915775602037 | 2.642920381479491 | 1.5165484376028202 | 2.3804101910757445 | 2.3649172012696487 | 2.790644912946584 | 1.9184968253245733 | 2.331547444480727 | 2.5215688806690455 | 2.569851993023285 | 2.3920831016177373 | 2.489080092262877 | 2.47600010537141 | 2.6472406584739985 | 2.783395955957251 | 2.636518407731503 | 3.2018038692410347 | 2.513779265994739 | 2.6155284290353933 | 2.6740147404714256 | 3.2693700557333867 | 2.4541111477982986 | 2.6315516589434083 | 2.7266704144443397 | 2.811993209468232 |
| ALA | 11.440483601987328 | 10.835915908032705 | 11.260482171177152 | 10.190313791799973 | 11.129310782018928 | 11.022337881388145 | 10.674175681493827 | 9.658686426833754 | 10.880425166371445 | 10.505604805194945 | 11.745898886978745 | 10.153168075967978 | 11.263183094243608 | 9.697069087211814 | 11.447385732390288 | 11.078192296915118 | 10.472608361380427 | 10.462025578578828 | 10.44012963778844 | 10.729571896975422 | 12.486824451455885 | 11.624935722888047 | 12.08050425918695 | 10.589774363042846 | 10.974792205874618 | 11.987753541500075 | 13.631927658205523 | 13.631379058525498 | 12.697646656033406 | 13.444169199416429 |
| SDA | 1.8394576302187162 | 2.2553913575314906 | 2.2367271839152707 | 2.1215606247421492 | 2.8009171330681797 | 2.5884476186703975 | 2.9326964585593025 | 2.120701565268797 | 3.0714519882897067 | 2.5201033314574386 | 2.870283548920495 | 2.3392395160581385 | 3.035429031254128 | 2.6554326306863136 | 3.278626421065383 | 3.0489093392588726 | 3.1690287025037023 | 3.33929288385166 | 3.5314279131762496 | 3.497581153506052 | 3.116948097048452 | 4.141289436311754 | 3.4730985193052364 | 3.7855943389054527 | 3.927194635325452 | 4.295291101073213 | 4.623784744282771 | 4.295640647835309 | 3.5775865180522186 | 4.384092124851711 |
| C20:1 | 4.808191923065935 | 4.9241858411893 | 4.503532249357354 | 4.394000118543675 | 4.2148053314453175 | 4.108721432742495 | 3.584308015357118 | 4.061018771481302 | 3.7132683064886254 | 4.244173936065727 | 4.54204504433302 | 4.261688129305153 | 3.9656113310799657 | 4.531718044659765 | 3.2591040788267605 | 4.072102920445254 | 3.4372330589204103 | 3.6289049674554836 | 3.714360575676163 | 3.6700815118627337 | 3.749374145706912 | 3.7528838260624626 | 3.7941415981524274 | 4.042913826810706 | 4.067692696074014 | 3.8932476367162288 | 3.6027095954145265 | 3.7653345819219033 | 3.5425755450036815 | 3.66258121486564 |
| DGLA | 1.651630385369183 | 1.7175355841045152 | 1.3804817405950902 | 1.546965692166175 | 1.2662028533933125 | 1.3658237222377296 | 1.7549343406755384 | 1.107460607863891 | 1.4731603192406215 | 1.5756151804970915 | 1.6642958839312119 | 1.396258972037684 | 1.4823832661045229 | 1.7325344529057272 | 1.4381326080960684 | 1.3779836839237785 | 1.685231791563348 | 1.4181835558383071 | 1.7222359003556185 | 1.69755126726904 | 1.5629710543036894 | 1.6978327641513644 | 1.2565913714220036 | 1.6358975520923058 | 1.570703625699448 | 1.669590166901276 | 1.313668892655194 | 1.4947448234261502 | 1.5078101040667258 | 1.6592941337717122 |
| ARA | 4.7405993465554 | 5.55492795933459 | 5.441976373078631 | 5.734133183524689 | 5.261399646015334 | 5.627620568701818 | 5.73443643683659 | 4.4484761156167085 | 5.2657980053873485 | 5.052834600145476 | 5.383022675416824 | 5.514474905576607 | 5.168741764242139 | 5.836450677509528 | 5.723260542075346 | 5.499277182207568 | 5.639621922792125 | 5.308151930137949 | 5.649853083145526 | 5.516544924920808 | 5.222550999634613 | 5.444484517467174 | 5.031706552124009 | 4.808646810407299 | 4.855726782177489 | 4.933031559636494 | 3.4014212672793374 | 3.7904704976486303 | 4.881077472680865 | 3.8843720078531847 |
| ETA | 2.075338982302341 | 1.9203722112854527 | 1.6808952428413955 | 1.7969800402593206 | 1.6330191065932078 | 1.780629130668663 | 2.1997088193467937 | 1.5543835007084996 | 2.049409604231725 | 1.9188898045125065 | 2.1105148331910426 | 1.8594930175955533 | 2.06260313703578 | 2.3787443742008545 | 2.047534278862829 | 1.8783603053395108 | 2.262003163500327 | 2.1370987696216712 | 2.4540114339436667 | 2.480187234949507 | 2.283939421548659 | 2.6347965362493624 | 2.1279543304075643 | 2.623880884616957 | 2.6011076924296708 | 2.588513466340315 | 3.2663435162129 | 3.2136006302124325 | 2.6985844424605885 | 3.6041759923754983 |
| EPA | 16.281206968622506 | 17.606101508072737 | 18.786640310522312 | 19.322529213257944 | 19.403513857534804 | 19.73085685449826 | 20.34722034627886 | 20.39915697966946 | 20.635091409366296 | 20.703037655508723 | 20.710640337939775 | 20.92277375311428 | 21.130674984101166 | 21.154456848705628 | 21.436143925768494 | 22.159008487477358 | 22.904703674049436 | 22.92102225203056 | 23.428040049610765 | 23.497033128736515 | 23.503751985664444 | 23.902293281253723 | 24.173920804244787 | 24.18079404514788 | 24.348087189091032 | 24.98015794983058 | 26.340964156662004 | 27.35438052431537 | 27.48025515640483 | 27.7739033714863 |
| DPA | 0.4439420000295093 | 0.42186243871591667 | 0.5056974112100333 | 0.5335383236961065 | 0.48791715217218834 | 0.4873735452337702 | 0.5923743614202771 | 0.6838507421163462 | 0.595304398039245 | 0.6207668914158841 | 0.6026589957307572 | 0.6235140143520276 | 0.6061227421820561 | 0.930664845400553 | 0.5795733191778152 | 0.6588680889250784 | 0.6942081426992533 | 0.7649122947476251 | 0.7065342517636014 | 0.803957446987178 | 0.6113917629606725 | 0.7648653993006855 | 0.7966313481429114 | 0.8636169637166023 | 0.9069964072036394 | 0.6373591542670468 | 0.8860546696366405 | 0.9424534120861938 | 0.805799522578935 | 0.8680003487782515 |
| Others | 7.9965775549675495 | 11.67590665242573 | 9.542790779771606 | 10.209497522198395 | 9.710604817899602 | 10.467338057135997 | 10.54524562754392 | 9.813789811606819 | 13.009268193956247 | 10.491166001053006 | 10.507861180732851 | 7.933689350451413 | 9.073987664620109 | 8.4380154572377 | 9.83420158914055 | 8.164052234747633 | 8.945878751195082 | 8.171647877980902 | 9.617761127626482 | 8.461626694573928 | 8.53323044885885 | 8.424588076429046 | 8.948611599528991 | 9.127113705473851 | 8.625339985649791 | 9.519117610501064 | 9.485706048990211 | 9.065710931795213 | 9.243407765360033 | 8.25699925743833 |

## Slide 10
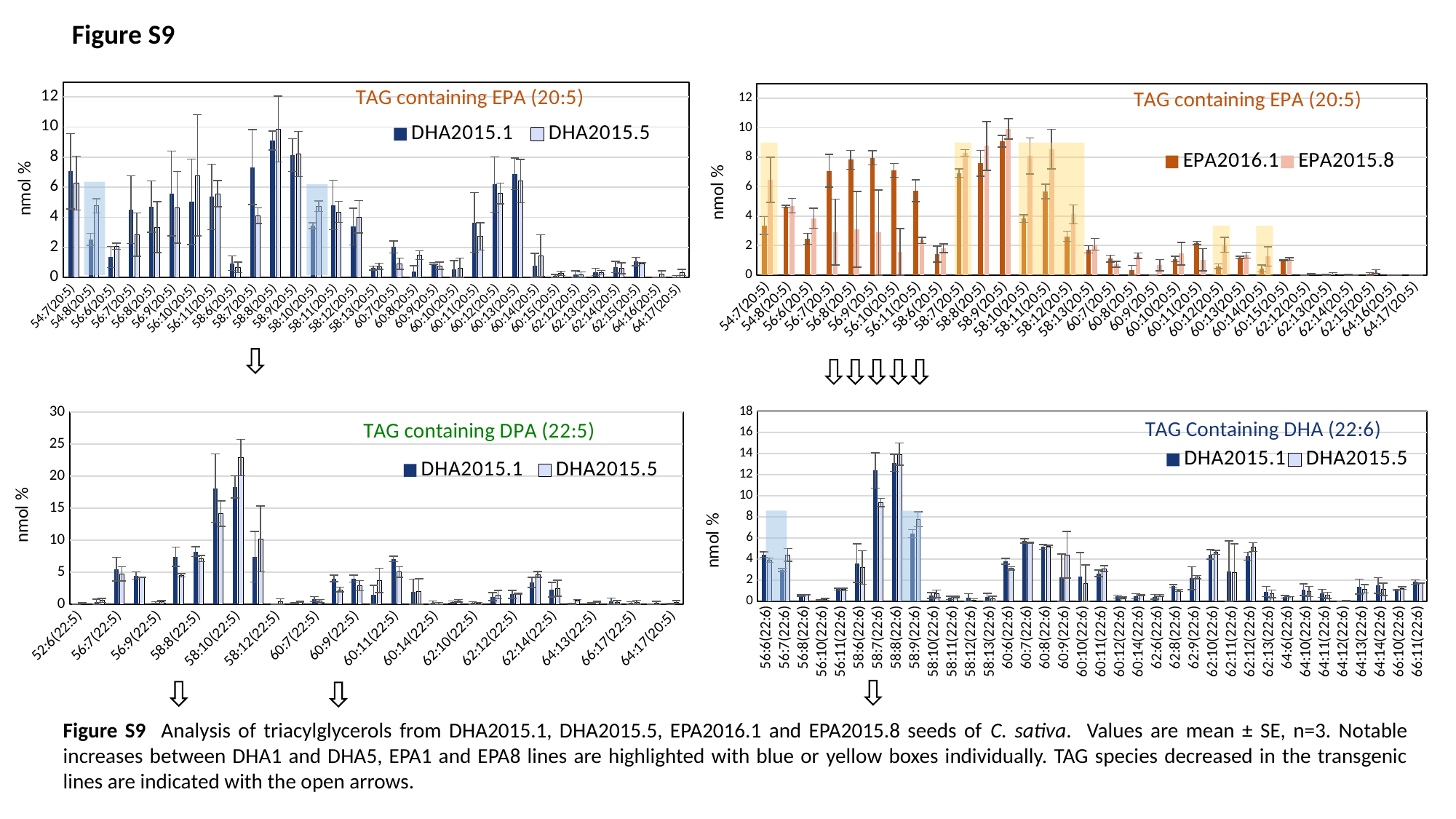

Figure S9
### Chart: TAG containing EPA (20:5)
| Category | EPA2016.1 | EPA2015.8 |
|---|---|---|
| 54:7(20:5) | 3.357480447121958 | 6.460481692179985 |
| 54:8(20:5) | 4.653210910139798 | 4.701511990471174 |
| 56:6(20:5) | 2.4602543813926174 | 3.8607174937215323 |
| 56:7(20:5) | 7.078129404047235 | 2.9208208348518525 |
| 56:8(20:5) | 7.825304507932452 | 3.107447188889427 |
| 56:9(20:5) | 7.959529483618385 | 2.8824367921696563 |
| 56:10(20:5) | 7.100414419539174 | 1.5773380432313913 |
| 56:11(20:5) | 5.727665207453046 | 2.343806830004962 |
| 58:6(20:5) | 1.419178794776994 | 1.8106495474332795 |
| 58:7(20:5) | 6.917211956878146 | 8.286845298305114 |
| 58:8(20:5) | 7.590806855243723 | 8.760580148691549 |
| 58:9(20:5) | 9.085147258731274 | 9.92772948137072 |
| 58:10(20:5) | 3.833044524810088 | 8.083362464355703 |
| 58:11(20:5) | 5.6615461869416706 | 8.560773991947833 |
| 58:12(20:5) | 2.6242827583689987 | 4.123021428667954 |
| 58:13(20:5) | 1.7359469300154207 | 2.084132556135308 |
| 60:7(20:5) | 1.1204669530110893 | 0.7295830456474697 |
| 60:8(20:5) | 0.32765649986339174 | 1.3112636945623883 |
| 60:9(20:5) | 0.0 | 0.6661360870170294 |
| 60:10(20:5) | 1.0884201581881296 | 1.4498309551461874 |
| 60:11(20:5) | 2.1777779453434074 | 1.0285904196908153 |
| 60:12(20:5) | 0.5943186295296905 | 2.061402888526254 |
| 60:13(20:5) | 1.1821070992351295 | 1.3524940311846068 |
| 60:14(20:5) | 0.4525360954365844 | 1.2618376427333735 |
| 60:15(20:5) | 0.9891367143563966 | 1.0888735823498739 |
| 62:12(20:5) | 0.0 | 0.0685559157144922 |
| 62:13(20:5) | 0.021485864007506832 | 0.10411471873001048 |
| 62:14(20:5) | 0.03332687207390578 | 0.0 |
| 62:15(20:5) | 0.0745667726539405 | 0.22464968881743133 |
| 64:16(20:5) | 0.0 | 0.0 |
| 64:17(20:5) | 0.0 | 0.0 |
### Chart: TAG containing EPA (20:5)
| Category | DHA2015.1 | DHA2015.5 |
|---|---|---|
| 54:7(20:5) | 7.056333715301275 | 6.278791618038813 |
| 54:8(20:5) | 2.5323846223082613 | 4.7651041636655735 |
| 56:6(20:5) | 1.3665624080187626 | 2.0762923305628447 |
| 56:7(20:5) | 4.504418869236288 | 2.8593023093936374 |
| 56:8(20:5) | 4.702823421209651 | 3.34232096524214 |
| 56:9(20:5) | 5.586531244577336 | 4.663971684736642 |
| 56:10(20:5) | 5.0231608466191675 | 6.788711248071135 |
| 56:11(20:5) | 5.35903197391769 | 5.563933234138449 |
| 58:6(20:5) | 0.9445399540512193 | 0.6653261170262059 |
| 58:7(20:5) | 7.334404658871573 | 4.099753198785231 |
| 58:8(20:5) | 9.1001805708677 | 9.861652785926145 |
| 58:9(20:5) | 8.14703041065961 | 8.21541259769507 |
| 58:10(20:5) | 3.4436878119956185 | 4.741964783873459 |
| 58:11(20:5) | 4.82310698419716 | 4.370600397886488 |
| 58:12(20:5) | 3.378358486489889 | 4.026982554236068 |
| 58:13(20:5) | 0.6201893112622454 | 0.7440627608943661 |
| 60:7(20:5) | 2.033822091750896 | 0.9171221916813715 |
| 60:8(20:5) | 0.39687994419103867 | 1.4849438621943758 |
| 60:9(20:5) | 0.9446163252474685 | 0.7916614799560869 |
| 60:10(20:5) | 0.565494458361533 | 0.6428308135887751 |
| 60:11(20:5) | 3.6586280750180156 | 2.729797861062495 |
| 60:12(20:5) | 6.183295647246491 | 5.588788393881682 |
| 60:13(20:5) | 6.889246030164956 | 6.408087206116981 |
| 60:14(20:5) | 0.803379470239909 | 1.4523918674692202 |
| 60:15(20:5) | 0.10128556935070353 | 0.28094763133797745 |
| 62:12(20:5) | 0.2184860665911529 | 0.19135470269807875 |
| 62:13(20:5) | 0.3709290669265526 | 0.3472302425082516 |
| 62:14(20:5) | 0.696646161199301 | 0.6207473873210368 |
| 62:15(20:5) | 1.0651902174226118 | 0.9563995468499806 |
| 64:16(20:5) | 0.0 | 0.2281925090376398 |
| 64:17(20:5) | 0.06785316874898029 | 0.3335830679783049 |
### Chart: TAG containing DPA (22:5)
| Category | DHA2015.1 | DHA2015.5 |
|---|---|---|
| 52:6(22:5) | 0.0 | 0.1664003136190068 |
| 56:6(22:5) | 0.38804284999662636 | 0.5904519688924327 |
| 56:7(22:5) | 5.485357594492374 | 4.727859214737718 |
| 56:8(22:5) | 4.461875749842744 | 4.222979479414611 |
| 56:9(22:5) | 0.239063270782238 | 0.36808638055525095 |
| 58:7(22:5) | 7.420131291284476 | 4.5061695037099305 |
| 58:8(22:5) | 8.205871139848256 | 7.110893498943856 |
| 58:9(22:5) | 18.09271571304232 | 14.134396733472363 |
| 58:10(22:5) | 18.303487633963158 | 22.879878418884726 |
| 58:11(22:5) | 7.410799892861654 | 10.183491036570842 |
| 58:12(22:5) | 0.0 | 0.41583967320722487 |
| 60:5(22:5) | 0.20070537944116396 | 0.3512770476727021 |
| 60:7(22:5) | 0.7742396477100039 | 0.4966768293738113 |
| 60:8(22:5) | 4.001735849002391 | 2.275004203706396 |
| 60:9(22:5) | 3.993799062555082 | 2.8963150496757684 |
| 60:10(22:5) | 1.4608394823262583 | 3.699204964357205 |
| 60:11(22:5) | 7.068045855333206 | 5.02620863478831 |
| 60:12(22:5) | 1.951698481473261 | 1.9726036077462605 |
| 60:14(22:5) | 0.24252668670530764 | 0.13363859270207826 |
| 62:6(22:5) | 0.3390814480153603 | 0.5332147530472121 |
| 62:10(22:5) | 0.1864750359588936 | 0.14564549599142826 |
| 62:11(22:5) | 1.169875794376531 | 1.4830552321683304 |
| 62:12(22:5) | 1.5410752354324533 | 1.6846906026637367 |
| 62:13(22:5) | 3.399327240626364 | 4.642440045876991 |
| 62:14(22:5) | 2.2554422512978602 | 2.4718889682509855 |
| 62:15(22:5) | 0.07320091910820808 | 0.6232111964189068 |
| 64:13(22:5) | 0.1812969576391887 | 0.34781806472379007 |
| 64:15(22:5) | 0.4878616663199979 | 0.2704809573815244 |
| 66:17(22:5) | 0.19106675742718582 | 0.2957815672908144 |
| 64:16(20:5) | 0.0 | 0.2281925090376398 |
| 64:17(20:5) | 0.06785316874898029 | 0.3335830679783049 |
### Chart: TAG Containing DHA (22:6)
| Category | DHA2015.1 | DHA2015.5 |
|---|---|---|
| 56:6(22:6) | 4.4439812181845655 | 3.8907602414602542 |
| 56:7(22:6) | 2.9712475309209254 | 4.391504526862024 |
| 56:8(22:6) | 0.539644107922604 | 0.5961288723195296 |
| 56:10(22:6) | 0.07090353761610084 | 0.16810352221366187 |
| 56:11(22:6) | 1.1564575924405018 | 1.1206203239841124 |
| 58:6(22:6) | 3.621398301127099 | 3.2084011434250272 |
| 58:7(22:6) | 12.405735338382257 | 9.367364509875058 |
| 58:8(22:6) | 13.119411322550194 | 13.952640198204945 |
| 58:9(22:6) | 6.416226834689645 | 7.776452470463696 |
| 58:10(22:6) | 0.5468281822545917 | 0.7138169136761204 |
| 58:11(22:6) | 0.3373675316615441 | 0.4125686393087539 |
| 58:12(22:6) | 0.35756531957620297 | 0.12223842155075061 |
| 58:13(22:6) | 0.38095352828701134 | 0.24756380689577587 |
| 60:6(22:6) | 3.794817891252293 | 3.1292147858186805 |
| 60:7(22:6) | 5.717375712256737 | 5.570821311812068 |
| 60:8(22:6) | 5.157660933633278 | 5.2220456561161805 |
| 60:9(22:6) | 2.244056277222726 | 4.408602214344331 |
| 60:10(22:6) | 2.3287264943889134 | 1.7227390353565915 |
| 60:11(22:6) | 2.651964790040237 | 3.109942047160208 |
| 60:12(22:6) | 0.44930966897853214 | 0.31574694899020334 |
| 60:14(22:6) | 0.4840953177399225 | 0.6086077465749327 |
| 62:6(22:6) | 0.4404195620114791 | 0.5592116861322302 |
| 62:8(22:6) | 1.43162407522867 | 1.0321303055466846 |
| 62:9(22:6) | 2.1894373560083324 | 2.2913219050813427 |
| 62:10(22:6) | 4.436462668954131 | 4.639698620577115 |
| 62:11(22:6) | 2.8588592409870217 | 2.7428131952018364 |
| 62:12(22:6) | 4.276502478956497 | 5.153300239148428 |
| 62:13(22:6) | 0.9151998685456754 | 0.7043826005699962 |
| 64:6(22:6) | 0.4213681600779418 | 0.4330668089616522 |
| 64:10(22:6) | 1.102699034280938 | 0.9248155385046277 |
| 64:11(22:6) | 0.7626174241988583 | 0.5721452785595091 |
| 64:12(22:6) | 0.0 | 0.02583476085559122 |
| 64:13(22:6) | 1.3846754924115026 | 1.1785505648771997 |
| 64:14(22:6) | 1.498211059843073 | 1.1339367113271457 |
| 66:10(22:6) | 1.0760571789861768 | 1.2470705847287602 |
| 66:11(22:6) | 1.8323536463410115 | 1.7314652086206463 |
Figure S9 Analysis of triacylglycerols from DHA2015.1, DHA2015.5, EPA2016.1 and EPA2015.8 seeds of C. sativa. Values are mean ± SE, n=3. Notable increases between DHA1 and DHA5, EPA1 and EPA8 lines are highlighted with blue or yellow boxes individually. TAG species decreased in the transgenic lines are indicated with the open arrows.

## Slide 11
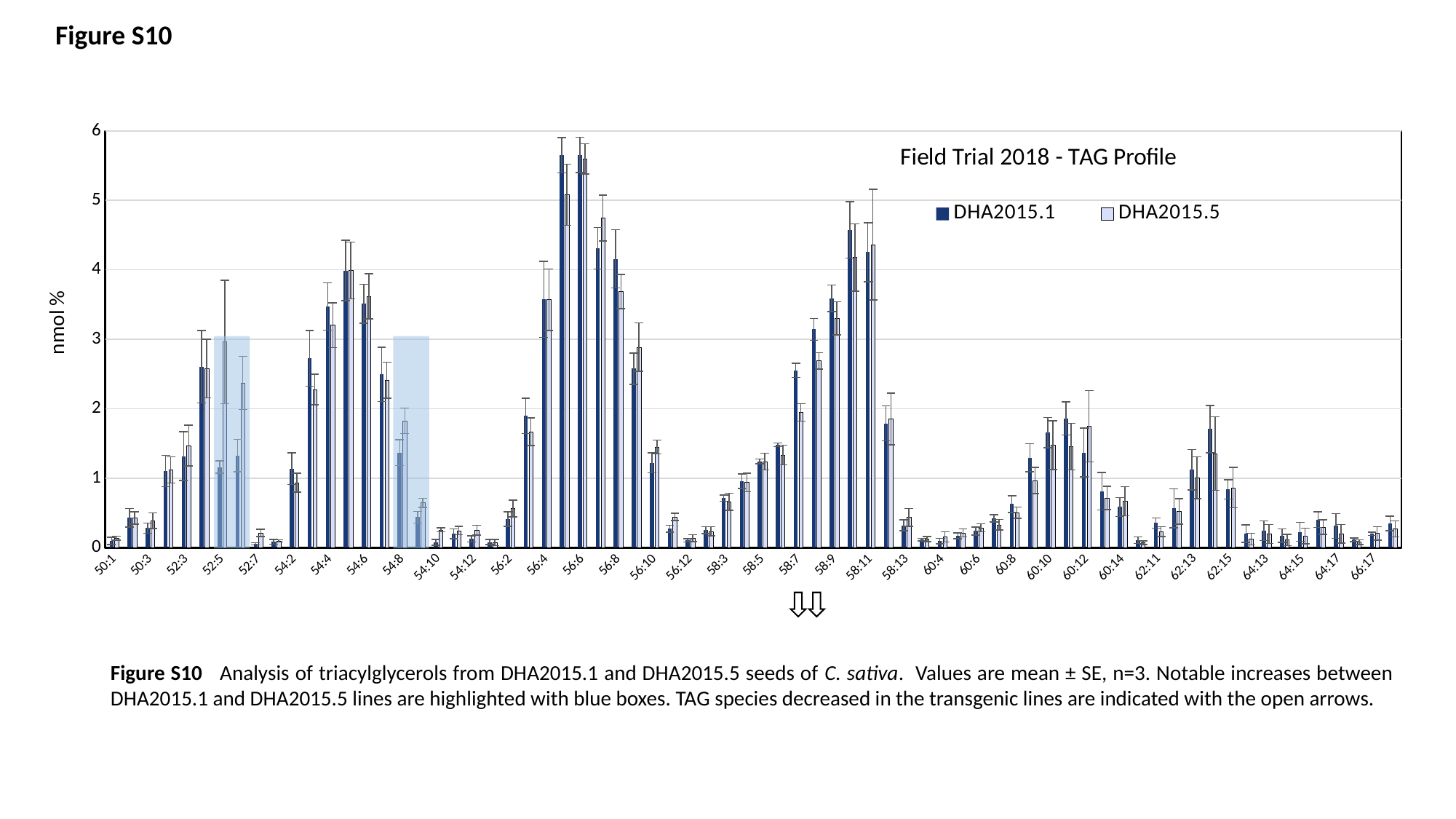

Figure S10
### Chart: Field Trial 2018 - TAG Profile
| Category | DHA2015.1 | DHA2015.5 |
|---|---|---|
| 50:1 | 0.10017326944068773 | 0.13806419947580464 |
| 50:2 | 0.43204070556597357 | 0.4300114129190566 |
| 50:3 | 0.282261333571465 | 0.39100710269635935 |
| 52:2 | 1.1062550896296808 | 1.121350820418921 |
| 52:3 | 1.3194641756072267 | 1.470587525429729 |
| 52:4 | 2.6053811523434116 | 2.578717939335139 |
| 52:5 | 1.1607626555812247 | 2.9637976631990344 |
| 52:6 | 1.3255510030332374 | 2.372852061535538 |
| 52:7 | 0.05721455616439741 | 0.21597096971019203 |
| 54:1 | 0.0858150160458846 | 0.09866175373761188 |
| 54:2 | 1.1401699614813123 | 0.936263627186278 |
| 54:3 | 2.726210368341856 | 2.278860620335137 |
| 54:4 | 3.474219086341846 | 3.2032796982840943 |
| 54:5 | 3.99165694439862 | 3.9916505764891705 |
| 54:6 | 3.512454635536273 | 3.61991207187558 |
| 54:7 | 2.494857527032355 | 2.4121940099192645 |
| 54:8 | 1.3680382646154159 | 1.827538428476398 |
| 54:9 | 0.44050705401321544 | 0.6484019435119123 |
| 54:10 | 0.07812816382179964 | 0.25760330937369447 |
| 54:11 | 0.1997074798330242 | 0.2479313104933637 |
| 54:12 | 0.12784554064651496 | 0.2539016393638335 |
| 56:1 | 0.08130566971186305 | 0.07540491279824188 |
| 56:2 | 0.41514632280207436 | 0.5650402604303278 |
| 56:3 | 1.8984402516437555 | 1.6689489883017783 |
| 56:4 | 3.575837453425102 | 3.5700300608421145 |
| 56:5 | 5.649697999043709 | 5.081396380760715 |
| 56:6 | 5.655648299132253 | 5.595323302884375 |
| 56:7 | 4.3090382501531215 | 4.745796628305599 |
| 56:8 | 4.158030849699048 | 3.6879835354700155 |
| 56:9 | 2.578207172620438 | 2.8883176283747587 |
| 56:10 | 1.2213470329162572 | 1.4485625916273908 |
| 56:11 | 0.2771374453065527 | 0.4451606287856646 |
| 56:12 | 0.10532681504640327 | 0.1387137065833591 |
| 58:2 | 0.2540314520914156 | 0.2363688044216142 |
| 58:3 | 0.7142917410694293 | 0.6620189518122794 |
| 58:4 | 0.9578702723329332 | 0.9409273808543294 |
| 58:5 | 1.2413834921246236 | 1.2412500248151705 |
| 58:6 | 1.4835789869146392 | 1.3362385353858848 |
| 58:7 | 2.5540529541189136 | 1.9466066750211892 |
| 58:8 | 3.1434694345716454 | 2.690869272734433 |
| 58:9 | 3.59116500343786 | 3.3013820451927898 |
| 58:10 | 4.5746224326374065 | 4.179910360639017 |
| 58:11 | 4.253554671629423 | 4.36212334037124 |
| 58:12 | 1.790286079288225 | 1.8548658836871086 |
| 58:13 | 0.32330018095021934 | 0.43741602546809205 |
| 60:3 | 0.11567696333753202 | 0.12360189404556292 |
| 60:4 | 0.09758748932901806 | 0.15753629218796952 |
| 60:5 | 0.1712552354766048 | 0.21398283160835363 |
| 60:6 | 0.242417606254552 | 0.2869819677928635 |
| 60:7 | 0.423504278921282 | 0.3313023622047151 |
| 60:8 | 0.6282053345961449 | 0.5056233458084627 |
| 60:9 | 1.29672368650533 | 0.9679978151973433 |
| 60:10 | 1.6573604925163232 | 1.4768331769594152 |
| 60:11 | 1.8609840074242305 | 1.456919862292528 |
| 60:12 | 1.3696555460784008 | 1.7490994804810238 |
| 60:13 | 0.813346758694332 | 0.71630586346282 |
| 60:14 | 0.5872338397274198 | 0.6707831230051687 |
| 60:15 | 0.1072561091257959 | 0.07340600198293185 |
| 62:11 | 0.35639521366871724 | 0.23400152768800594 |
| 62:12 | 0.5678905465697178 | 0.5220907307169195 |
| 62:13 | 1.1223978599648696 | 1.0056785620529423 |
| 62:14 | 1.7076425858162796 | 1.356735265945127 |
| 62:15 | 0.8384864367442848 | 0.8657954443667131 |
| 62:16 | 0.20433362487436021 | 0.124297065334804 |
| 64:13 | 0.24394778617692295 | 0.20013774275539595 |
| 64:14 | 0.1762444769545084 | 0.1123700400893682 |
| 64:15 | 0.2300854958260481 | 0.16822162200902868 |
| 64:16 | 0.4068686353771774 | 0.2989414209333748 |
| 64:17 | 0.31485247725699966 | 0.20134378202142147 |
| 66:16 | 0.11452235237884323 | 0.08066398854537349 |
| 66:17 | 0.2018016050530305 | 0.2083262777189158 |
| 66:18 | 0.35227287024820625 | 0.2710477738052796 |
Figure S10 Analysis of triacylglycerols from DHA2015.1 and DHA2015.5 seeds of C. sativa. Values are mean ± SE, n=3. Notable increases between DHA2015.1 and DHA2015.5 lines are highlighted with blue boxes. TAG species decreased in the transgenic lines are indicated with the open arrows.

## Slide 12
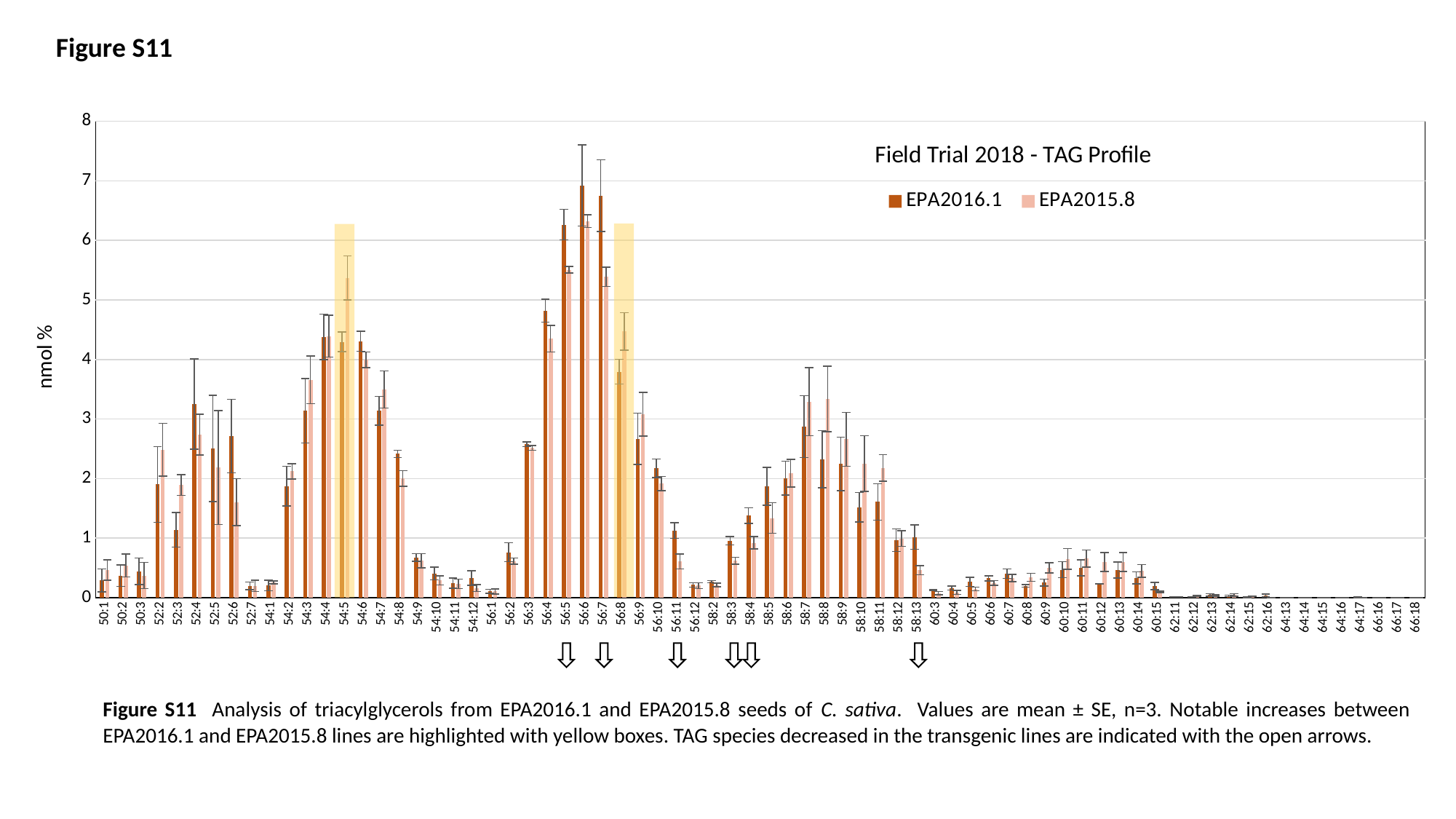

Figure S11
#
### Chart: Field Trial 2018 - TAG Profile
| Category | EPA2016.1 | EPA2015.8 |
|---|---|---|
| 50:1 | 0.2881271182042812 | 0.4648029264877662 |
| 50:2 | 0.3681513479812017 | 0.540586219447299 |
| 50:3 | 0.4431056955197443 | 0.37132910171227484 |
| 52:2 | 1.900898928717181 | 2.483959190590182 |
| 52:3 | 1.1386559757034564 | 1.8920205101887426 |
| 52:4 | 3.252680985474521 | 2.7353365001864005 |
| 52:5 | 2.505883894350784 | 2.182845120460001 |
| 52:6 | 2.7129196368181447 | 1.6031773049890994 |
| 52:7 | 0.1990172926861108 | 0.19982407785811926 |
| 54:1 | 0.20394623437849488 | 0.256447857654294 |
| 54:2 | 1.8698259564352433 | 2.120796195096562 |
| 54:3 | 3.137848793344933 | 3.6576465085273795 |
| 54:4 | 4.378155129794908 | 4.390916472922119 |
| 54:5 | 4.29567142208117 | 5.367990817063663 |
| 54:6 | 4.306798418879144 | 3.993309169866334 |
| 54:7 | 3.1390284904595807 | 3.4927433531333905 |
| 54:8 | 2.4145467421042217 | 2.000270026844288 |
| 54:9 | 0.6751998288357134 | 0.6191273074332909 |
| 54:10 | 0.4050829713443895 | 0.2917352821435029 |
| 54:11 | 0.245662937970439 | 0.23253144244261334 |
| 54:12 | 0.3310005857269692 | 0.16253366944343817 |
| 56:1 | 0.10704680689425494 | 0.1053294773650185 |
| 56:2 | 0.7618412480526157 | 0.6144668264287168 |
| 56:3 | 2.5750372499140664 | 2.5124969781980457 |
| 56:4 | 4.8166453750517375 | 4.349037765391855 |
| 56:5 | 6.263328023162689 | 5.505527799407983 |
| 56:6 | 6.919755405363304 | 6.323532555648231 |
| 56:7 | 6.751378944927953 | 5.3884541189271085 |
| 56:8 | 3.7919988790495682 | 4.4692981265201155 |
| 56:9 | 2.6674720806386265 | 3.0797628310827783 |
| 56:10 | 2.1718293085838964 | 1.9150512197183858 |
| 56:11 | 1.1281431845218335 | 0.6087320241240308 |
| 56:12 | 0.21491474836585547 | 0.20223381579728783 |
| 58:2 | 0.26772233345516777 | 0.21318082321759757 |
| 58:3 | 0.9556462424533679 | 0.6198235683718504 |
| 58:4 | 1.3747012105856704 | 0.9210292092212674 |
| 58:5 | 1.871926602197785 | 1.3364329858272435 |
| 58:6 | 2.0045145319949755 | 2.09065071084103 |
| 58:7 | 2.8718492183374624 | 3.292695962588205 |
| 58:8 | 2.3230053692742882 | 3.3374544238575976 |
| 58:9 | 2.246905538412996 | 2.6580597344068693 |
| 58:10 | 1.516841283043323 | 2.2528719859275514 |
| 58:11 | 1.6068693858664156 | 2.1779627332632874 |
| 58:12 | 0.9645569514022864 | 0.9919420663209578 |
| 58:13 | 1.0180472602368509 | 0.45902101940088946 |
| 60:3 | 0.12762358027516288 | 0.07446613701800858 |
| 60:4 | 0.161266663853921 | 0.08929709903203471 |
| 60:5 | 0.2655961055046269 | 0.14352422118094824 |
| 60:6 | 0.3235770816767292 | 0.24545689393857173 |
| 60:7 | 0.4004103202919882 | 0.3301847402578003 |
| 60:8 | 0.19761204404198832 | 0.33981273290960096 |
| 60:9 | 0.2550807743430496 | 0.499059650816104 |
| 60:10 | 0.46976228923750546 | 0.6506433685067655 |
| 60:11 | 0.49988132907151134 | 0.6572083131523018 |
| 60:12 | 0.2329298432471597 | 0.596510279154427 |
| 60:13 | 0.4628616814966695 | 0.5980750895750649 |
| 60:14 | 0.33268918028893746 | 0.4490948128215832 |
| 60:15 | 0.19325547201078655 | 0.09601792725178242 |
| 62:11 | 0.012032728935737735 | 0.016564479965623907 |
| 62:12 | 0.009933850411371263 | 0.029894660802600295 |
| 62:13 | 0.04428312675507703 | 0.03556033468718941 |
| 62:14 | 0.02965875398306622 | 0.04603962978954252 |
| 62:15 | 0.005904633860589079 | 0.024703125345055046 |
| 62:16 | 0.03498399641550887 | 0.0 |
| 64:13 | 0.0 | 0.0 |
| 64:14 | 0.0015539679839563778 | 0.0 |
| 64:15 | 0.0016519829882606788 | 0.0005914678863917234 |
| 64:16 | 0.00162342852025276 | 0.0021689623449719472 |
| 64:17 | 0.005970367561184347 | 0.0 |
| 66:16 | 0.0 | 0.0 |
| 66:17 | 0.0 | 0.002263919874382832 |
| 66:18 | 0.0 | 0.0 |
Figure S11 Analysis of triacylglycerols from EPA2016.1 and EPA2015.8 seeds of C. sativa. Values are mean ± SE, n=3. Notable increases between EPA2016.1 and EPA2015.8 lines are highlighted with yellow boxes. TAG species decreased in the transgenic lines are indicated with the open arrows.
